# Covalent Organic Framework Nanoplates Enable Solution-Processed Crystalline Nanofilms for Photoelectrochemical Hydrogen Evolution

**DOI:** 10.1021/jacs.2c01433

**Published:** 2022-06-03

**Authors:** Liang Yao, Andrés Rodríguez-Camargo, Meng Xia, David Mücke, Roman Guntermann, Yongpeng Liu, Lars Grunenberg, Alberto Jiménez-Solano, Sebastian T. Emmerling, Viola Duppel, Kevin Sivula, Thomas Bein, Haoyuan Qi, Ute Kaiser, Michael Grätzel, Bettina V. Lotsch

**Affiliations:** † Nanochemistry Department, Max Planck Institute for Solid State Research, Heisenbergstraße 1, 70569 Stuttgart, Germany; ‡ Laboratory of Photonics and Interfaces, École Polytechnique Fédérale de Lausanne, Station 6, 1015 Lausanne, Switzerland; § Central Facility for Materials Science Electron Microscopy, Ulm University, 89081 Ulm, Germany; ∥ Department of Chemistry and Center for NanoScience (CeNS), Ludwig-Maximilians-Universität München, Butenandtstraße 5-13 (E), 81377 Munich, Germany; ⊥ Laboratory for Molecular Engineering of Optoelectronic Nanomaterials, École Polytechnique Fédérale de Lausanne (EPFL), Station 6, 1015 Lausanne, Switzerland; # Department of Chemistry, University of Stuttgart, Pfaffenwaldring 55, 70569 Stuttgart, Germany; ∇ Department of Chemistry, Ludwig-Maximilians-Universität München, Butenandtstraße 5-13, 81377 Munich, Germany; ○ E-Conversion and Center for Nanoscience, Lichtenbergstraße 4a, Garching bei München, 85748 Munich, Germany; ◆ Center for Advancing Electronics Dresden (CFAED) and Faculty of Chemistry and Food Chemistry, Technische Universität Dresden, 01062 Dresden, Germany

## Abstract

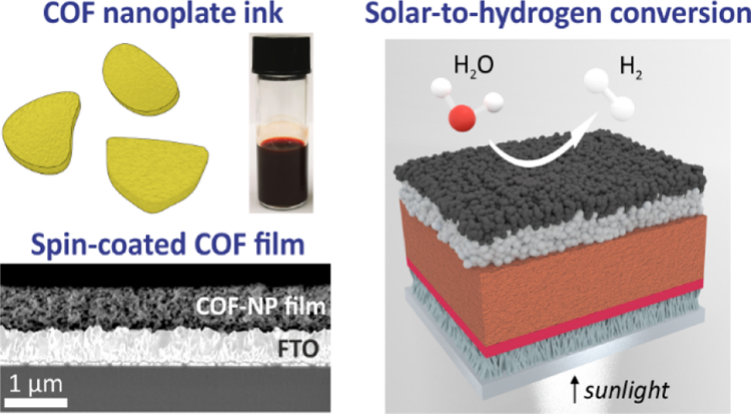

As covalent organic frameworks (COFs) are coming of age, the lack of effective approaches to achieve crystalline and centimeter-scale-homogeneous COF films remains a significant bottleneck toward advancing the application of COFs in optoelectronic devices. Here, we present the synthesis of colloidal COF nanoplates, with lateral sizes of ∼200 nm and average heights of 35 nm, and their utilization as photocathodes for solar hydrogen evolution. The resulting COF nanoplate colloid exhibits a unimodal particle-size distribution and an exceptional colloidal stability without showing agglomeration after storage for 10 months and enables smooth, homogeneous, and thickness-tunable COF nanofilms via spin coating. Photoelectrodes comprising COF nanofilms were fabricated for photoelectrochemical (PEC) solar-to-hydrogen conversion. By rationally designing multicomponent photoelectrode architectures including a polymer donor/COF heterojunction and a hole-transport layer, charge recombination in COFs is mitigated, resulting in a significantly increased photocurrent density and an extremely positive onset potential for PEC hydrogen evolution (over +1 V against the reversible hydrogen electrode), among the best of classical semiconductor-based photocathodes. This work thus paves the way toward fabricating solution-processed large-scale COF nanofilms and heterojunction architectures and their use in solar-energy-conversion devices.

## Introduction

Since the first successful realization of covalent organic frameworks (COFs) in 2005, COFs have matured as a platform for designing a new generation of porous polymers, combining superior crystallinity, adjustable pore metrics, and tolerance to functionalization.^1^ The physicochemical and optoelectronic properties of COFs can be easily tailored by condensation of customized molecular building blocks, which therefore opens the door to employ COFs in various practical applications.^2^ However, despite the fact that the long-range order potentially favors charge carrier transport, the success of applying COFs in optoelectronic devices is still far from established compared to conjugated molecular systems and linear polymers.^3,4^ Promoting this progress is currently limited by the poor processability of COFs since COFs are generally obtained as insoluble solids. Although various strategies toward preparing COF thin films have been developed in recent years,^5−8^ achieving centimeter-scale, homogeneous, and nanometer-thick COF films remains a formidable challenge.

COFs have recently been recognized as a novel class of photoabsorber candidates for solar-driven H_2_ evolution.^9^ Following the seminal report on COFs for photocatalytic H_2_ evolution by our group, various COFs have proven active as the photocatalyst for H_2_ evolution in the presence of a sacrificial donor and a H_2_ evolution co-catalyst.^10−13^ Alternatively, solar-driven H_2_ evolution is also attainable through a photoelectrochemical (PEC) approach, for which photogenerated electrons from the photoabsorber drive the H_2_ evolution reaction (HER) at a semiconductor film–electrolyte interface.^14,15^ While photoelectrochemistry could provide a profound fundamental understanding for charge separation in photoabsorbers as well as charge extraction for HER, the current bottleneck of developing PEC H_2_ evolution using COFs as the photoabsorber is the lack of effective solutions to fabricate COF thin films or heterojunction architectures that allow for efficiently harvesting the photogenerated charges in COFs.^16−20^ Although solvothermal synthesis^16^ and electrophoretic deposition^17^ have been used for preparing crystalline COF films and photoelectrodes, controlling film morphology and thickness with these methods is still challenging. Therefore, novel film fabrication approaches as well as associated strategies for mitigating photogenerated carrier recombination are needed.

Fabricating solution-processed thin films with colloidal semiconductor inks has proven to be one of the most competitive approaches for manufacturing optoelectronic semiconductor devices.^21−24^ Accordingly, developing colloidal COF nanoparticles holds the promise to overcome the difficulties associated with processing COFs. While exfoliating bulk COF powder into nanoparticles has been extensively explored, such an approach does not offer a concentration tunable colloid ink with unimodal particle-size distribution and therefore can hardly meet the requirements for preparing optoelectronic devices.^25−27^ An alternative emerging strategy for obtaining colloidal COF particles is the bottom–up synthesis, avoiding crystallite precipitation by tuning reaction conditions. Dichtel and co-workers developed an approach to synthesize stable colloidal suspensions of a series of COFs by using nitrile-containing solvents in the reaction.^28−30^ Taking advantage of this approach, single-crystalline boronate ester COF particles have been synthesized^31^ and oriented boronate ester COF thin films^32^ can be grown on graphene and monolayer MoS_2_. Nevertheless, the lack of conjugation and low hydrolytic stability of boronate ester and boroxine COFs hinder their application in optoelectronic devices.^4^ Colloidal COF particles based on the imine or ketoenamine linkage have also been reported by several groups.^33−35^ However, the reported colloidal COFs typically have particle sizes on the scale of several hundred nanometers to micrometers, which are too large for preparing smooth and homogeneous thin films with the thickness of a few hundred nanometers. Indeed, particle size and morphology control of colloidal COFs toward application in optoelectronic devices have not been reported so far.

**Figure 1:**
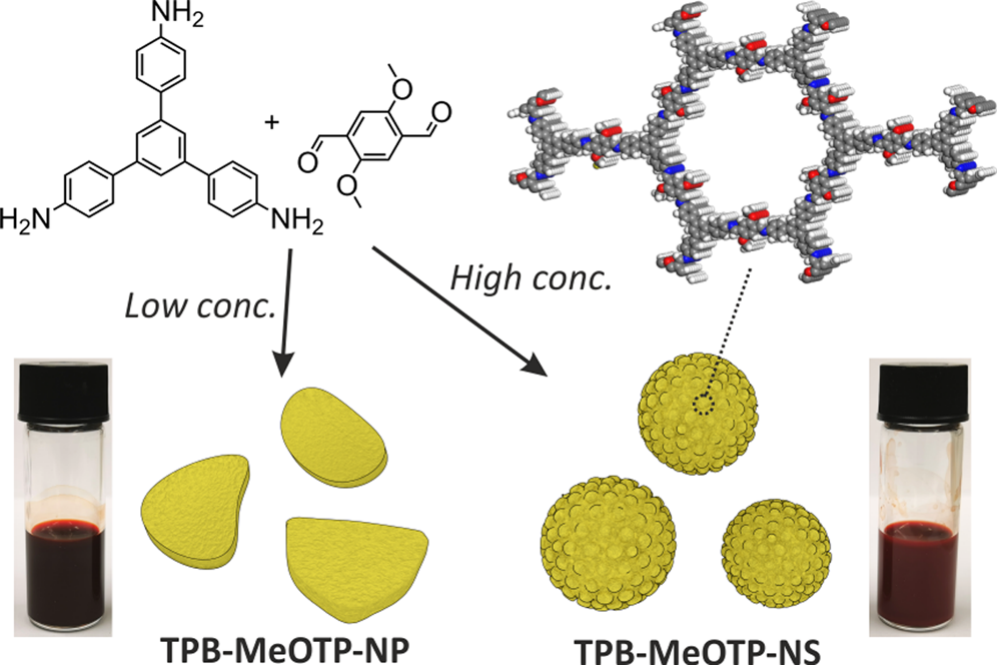
Schematic illustration of the synthesis of TPB-MeOTP nanoplates (TPB-MeOTP-NP) and nanospheres (TPB-MeOTP-NS). Structural representation of TPB-MeOTP COF and photographs of the TPB-MeOTP-NP and TPB-MeOTP-NS colloids with a concentration of 4.5 mg mL^–1^ in acetonitrile.

Herein, we reveal anisotropic particle growth for a colloidal imine COF through the rational choice of building blocks and reaction conditions (Figure 1). Different from the reported modulator approach for boronate ester COF,^36^ we achieve colloidal imine COF nanoplates, showing preferential growth along the COF interlayer stacking direction, driven by the self-assembly of the linker in solution. Spherical nanoparticles using the same linkers are also obtained by varying the precursor concentration for colloid synthesis. The resulting colloidal nanoplates and nanospheres are applied as inks for preparing solution-processed thin films and COF-based hydrogen evolution photoelectrodes. Compared to the reported COF photoelectrodes, our colloidal nanoplates enable the preparation of smooth and homogeneous films with a controllable thickness. More importantly, heterojunction films are successfully constructed, showing mitigated charge recombination in COFs. It is worth mentioning that this represents a significant advantage over other COF film fabrication schemes such as the solvothermal approach, as it is much more tolerant to delicate underlayers that are prone to getting destroyed under solvothermal conditions.

## Results and Discussion

**Figure 2:**
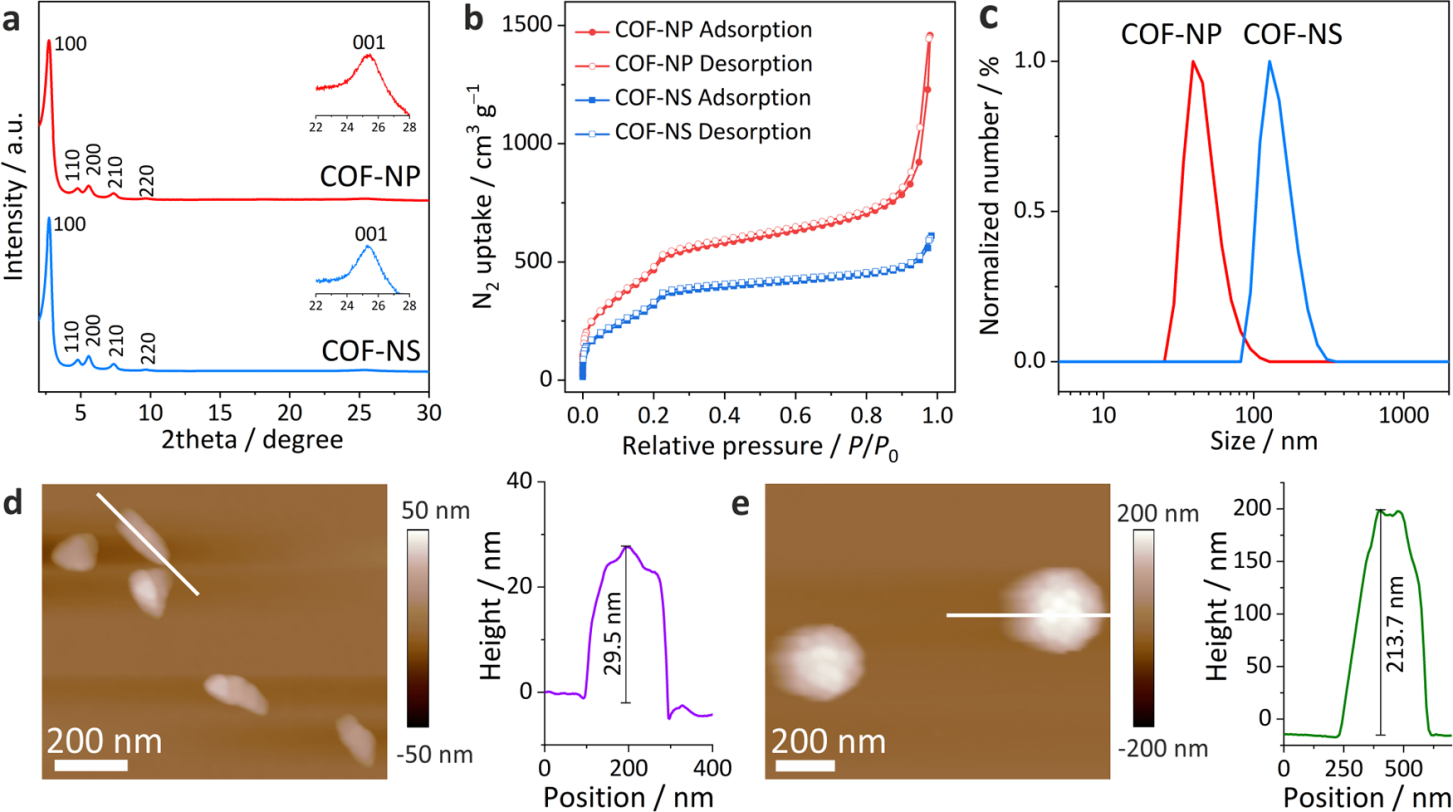
Crystallinity, porosity, and particle-size characterizations of TPB-MeOTP-NP and TPB-MeOTP-NS. (a) PXRD patterns (Cu Kα_1_). (b) N_2_ adsorption (filled) and desorption (empty) isotherm profiles at 77 K. (c) DLS number distributions of radius particle size. (d, e) Atomic force microscopy (AFM) height images of TPB-MeOTP-NP (d) and TPB-MeOTP-NS (e), respectively. The height profiles of representative particles are displayed along the line in the height images. COF-NP and COF-NS denote TPB-MeOTP-NP and TPB-MeOTP-NS, respectively.

As we aim toward employing COF photoelectrodes in the aqueous medium favorable for H_2_ evolution, i.e., in acidic or mildly acidic electrolytes, we sought to choose a robust COF that could withstand the challenging PEC operation conditions. Methoxy groups tend to reinforce COF interlayer interactions through noncovalent bonding, providing high robustness to the framework with respect to various pH treatments.^37,38^ Accordingly, we synthesized a TPB-MeOTP COF colloid with 1,3,5-tris(4-aminophenyl)benzene (TAPB) and 2,5-dimethoxyterephthalaldehyde (MeOTP) as building blocks (ratio of amine groups and aldehyde groups = 1:1), acetonitrile as the primary solvent, and Sc(OTf)_3_ as the catalyst. Contrary to the classical solvothermal approach, this reaction was conducted at room temperature. Interestingly, the particle shape of TPB-MeOTP is strongly related to the linker concentration. Nanoplates and nanospheres were obtained with [TAPB] less than 1.97 mM and larger than 3.69 mM, respectively, while intermediate [TAPB] resulted in a mixture of them (Supporting Figure S1). Representative nanoplates and nanospheres were synthesized using [TAPB] of 1.97 and 5.02 mM with 0.08 equiv Sc(OTf)_3_, respectively, to investigate the particle shape effect on COF properties, and the resulting products are coded as TPB-MeOTP-NP and TPB-MeOTP-NS. Powder X-ray diffraction (PXRD) measurements reveal that both TPB-MeOTP-NP and TPB-MeOTP-NS show six prominent diffraction peaks (Figure 2a), assigned to the 100, 110, 200, 210, 220, and 001 facets, and in agreement with TPB-MeOTP COF synthesized solvothermally (Supporting Figure S2). Pawley refinement was performed for the experimental PXRDs, indicating that TPB-MeOTP-NP and TPB-MeOTP-NS possess nearly identical unit cell parameters (Supporting Figure S3). In Fourier transform infrared (FT-IR) spectra of TPB-MeOTP-NP and TPB-MeOTP-NS, a C=N stretching vibration band at 1592 cm^–1^ is observed, confirming the imine formation (Supporting Figure S4). The porosity of TPB-MeOTP-NP and TPB-MeOTP-NS was measured by nitrogen adsorption (Figure 2b). Hysteresis-free type-IV isotherms indicative of mesoporous materials were obtained in both cases, and calculated pore-size distributions show identical pore sizes of 3.1 nm for both morphologies (Supporting Figure S5). The Brunauer–Emmett–Teller surface area (S_BET_) is 1688 and 1165 m^2^ g^–1^ for TPB-MeOTP-NP and TPB-MeOTP-NS, respectively (Supporting Figure S5). Dynamic light scattering (DLS) of TPB-MeOTP-NP and TPB-MeOTP-NS displays a unimodal size distribution (Figure 2c and multibatches shown in Supporting Figure S7), indicating both TPB-MeOTP-NP and TPB-MeOTP-NS possess a very homogeneous particle-size distribution. More importantly, TPB-MeOTP-NP and TPB-MeOTP-NS show a remarkable colloidal stability, without particle agglomeration upon increasing colloid concentration and long-term storage (10 months for TPB-MeOTP-NP, 6 months for TPB-MeOTP-NS, Supporting Figure S8) and without amorphization upon long-term storage (Supporting Figure S9).

**Figure 3:**
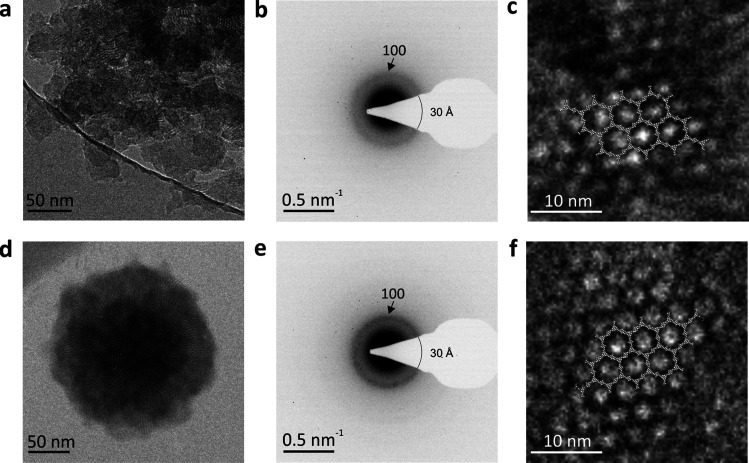
TEM characterizations of TPB-MeOTP-NP (a, b, c) and TPB-MeOTP-NS (d, e, f). (a, d) TEM images. (b, e) Selected-area electron diffraction (SAED) patterns. (c, f) HRTEM images. The Pawley refined structure models are overlaid with the HRTEM images.

Atomic force microscopy (AFM) and transmission electron microscopy (TEM) were employed to investigate the particle morphology difference. In the AFM topographical images of TPB-MeOTP-NP (Figure 2d), the particle height (29.5 nm) is significantly smaller than its lateral size (∼200 nm). Moreover, the TPB-MeOTP-NP particle height has a narrow distribution with an average value of ∼35 nm, calculated from 103 particles (Supporting Figure S10), thus hinting at an anisotropic particle growth. In comparison, TPB-MeOTP-NS clearly shows a spherical shape (Figure 2e), and the average height of TPB-MeOTP-NS of 87 particles is determined to be ∼160 nm (Supporting Figure S11). TEM characterization furnishes consistent information on the particle morphology: While TPB-MeOTP-NP forms plate-like nanoparticles, TPB-MeOTP-NS is spherical (Figure 3a,d, Supporting Figure S14). Selected-area electron diffractions (SAED) of both TPB-MeOTP-NP and TPB-MeOTP-NS show first-order reflections (100) at 0.33 nm^–1^, corresponding to a d-spacing of ∼3.0 nm (Figure 3b,e, Supporting Figure S12). High-resolution TEM (HRTEM) imaging further reveals that, despite their different morphology, both TPB-MeOTP-NP and TPB-MeOTP-NS consist of covalent honeycomb networks with identical lattice parameters, i.e., *a* = *b* = 3.5 nm, γ = 120° (Figure 3c,f, Supporting Figure S13). The TEM results are in agreement with the Pawley refined structure models derived by PXRD.

**Figure 4:**
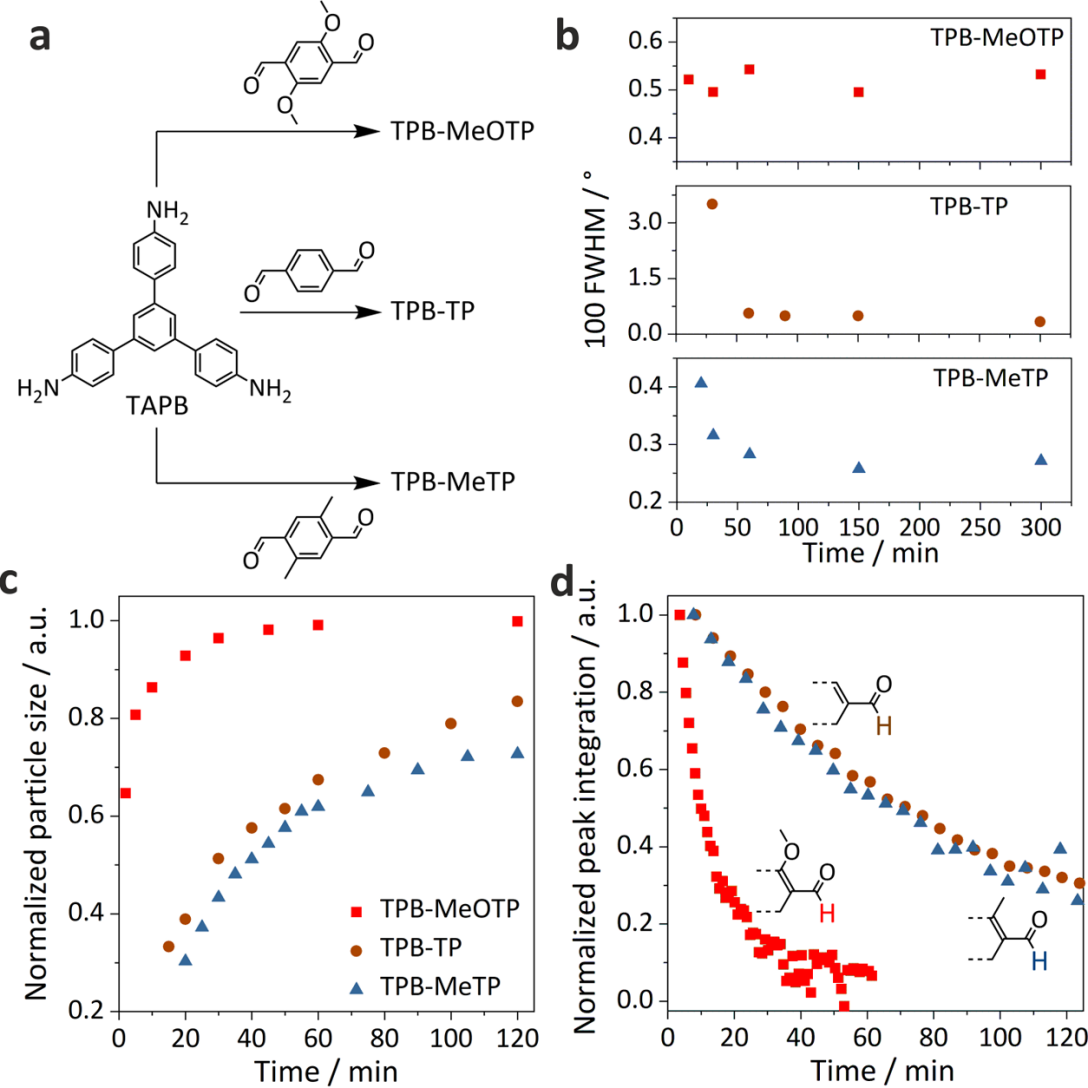
Kinetic study of the colloid reactions. (a) Building blocks of the TPB-MeOTP, TPB-TP, and TPB-MeTP condensation reactions. (b) FWHM of the 100 peak in the time-dependent XRD patterns. (c) Particle-size (radius) variation versus reaction time. The particle size is normalized with the size after 3 days (Supporting Figure S23). (d) Normalized aldehyde proton peak integration of in situ ^1^H NMR measurements as a function of reaction time.

It is noted that the methoxy substitution on terephthalaldehyde is crucial for the changes in particle morphology with respect to the reaction conditions. As shown in Figure 4a and Supporting Figures S15–S17, we employed a series of terephthalaldehyde monomers, i.e., without substitution (code as TP), and substituted with methyl and hydroxyl groups (MeTP and HTP). While TPB-TP and TPB-MeTP colloids were successfully obtained, TPB-TP and TPB-MeTP reactions result in spherical particles irrespective of the [TAPB] ranging between 3.69 and 0.52 mM. To shed light on the nanoplate formation of colloidal TPB-MeOTP COF, we investigated and compared the kinetics of TPB-MeOTP, TPB-TP, and TPB-MeTP colloid reactions under the synthesis condition of representative TPB-MeOTP-NP. PXRD patterns recorded at different reaction times and analysis of the FWHM of the 100 reflections indicate that the TPB-MeOTP colloid crystallizes fastest with the highest reaction yield (Figure 4b and Supporting Figures S18 and S19): Crystalline COF is formed after 10 min with a yield of 86%, high S_BET_ of 1357 m^2^ g^–1^, and visible 100 lattice fringes in the TEM image (Supporting Figures S20 and S21), and no obvious crystallinity enhancement is observed with extending reaction time. In comparison, TPB-TP and TPB-MeTP colloids show an apparently slower crystallization, as evidenced by the diffraction peak sharpening with respect to reaction time, and a lower reaction yield of ∼50% (Supporting Figure S19). Meanwhile, dynamic light scattering (DLS) and in situ proton nuclear magnetic resonance (^1^H NMR) were undertaken to inspect the particle growth and polymerization process (Figure 4c,d and Supporting Figures S22–S28). DLS measurements suggest that TPB-TP and TPB-MeTP particles keep growing during the first day, while TPB-MeOTP particle growth ceases after 1 h, indicative of a significantly faster particle growth process. As TAPB and terephthalaldehyde monomers polymerize into nanoparticles, it is expected that the proton peaks of the monomers as measured by in situ ^1^H NMR diminish over time. We note that the aldehyde proton consumption rate can be fitted by a first-order kinetic model (Supporting Figure S29), i.e., *I* = *I*_0_ e^–*kt*^, and the polymerization rate constant (*k*_p_) of TPB-MeOTP, TPB-TP, and TPB-MeTP colloid reactions is estimated to be 1.2 × 10^–3^ s^–1^, 1.5 × 10^–4^ s^–1^, and 1.4 × 10^–4^ s^–1^, respectively. Therefore, in situ ^1^H NMR suggests that the *k*_p_ of the TPB-MeOTP colloid reaction is one order of magnitude higher. Overall, the kinetic studies suggest that the TPB-MeOTP colloid reaction combines the features of larger *k*_p_, faster crystallization, more rapid particle growth, and higher reaction yield, compared to TPB-TP and TPB-MeTP reactions. This is likely correlated to the reinforced interlayer interaction of TPB-MeOTP COF retarding the hydrolysis of the imine bond, i.e., the reverse reaction, which is supported by the well-established enhanced stability of TPB-MeOTP in acidic media.^37^ The larger *k*_p_ of TPB-MeOTP therefore could result in the faster particle nucleation and growth as well as the faster consumption of TAPB and MeOTP linkers, as observed in DLS and in situ ^1^H NMR. In fact, a fast reaction rate and low reversibility of the Schiff base reaction are detrimental to obtaining crystalline COFs, which is why TPB-MeOTP colloids show comparatively smaller particle sizes and lower crystallinity than the TP and MeTP counterparts. Nevertheless, the TPB-MeOTP system combines the merits of fast crystallization and high yield. We hypothesized that this is a result of MeOTP building block self-assembly, driven by its noncovalent interactions in the reaction solution.

**Figure 5:**
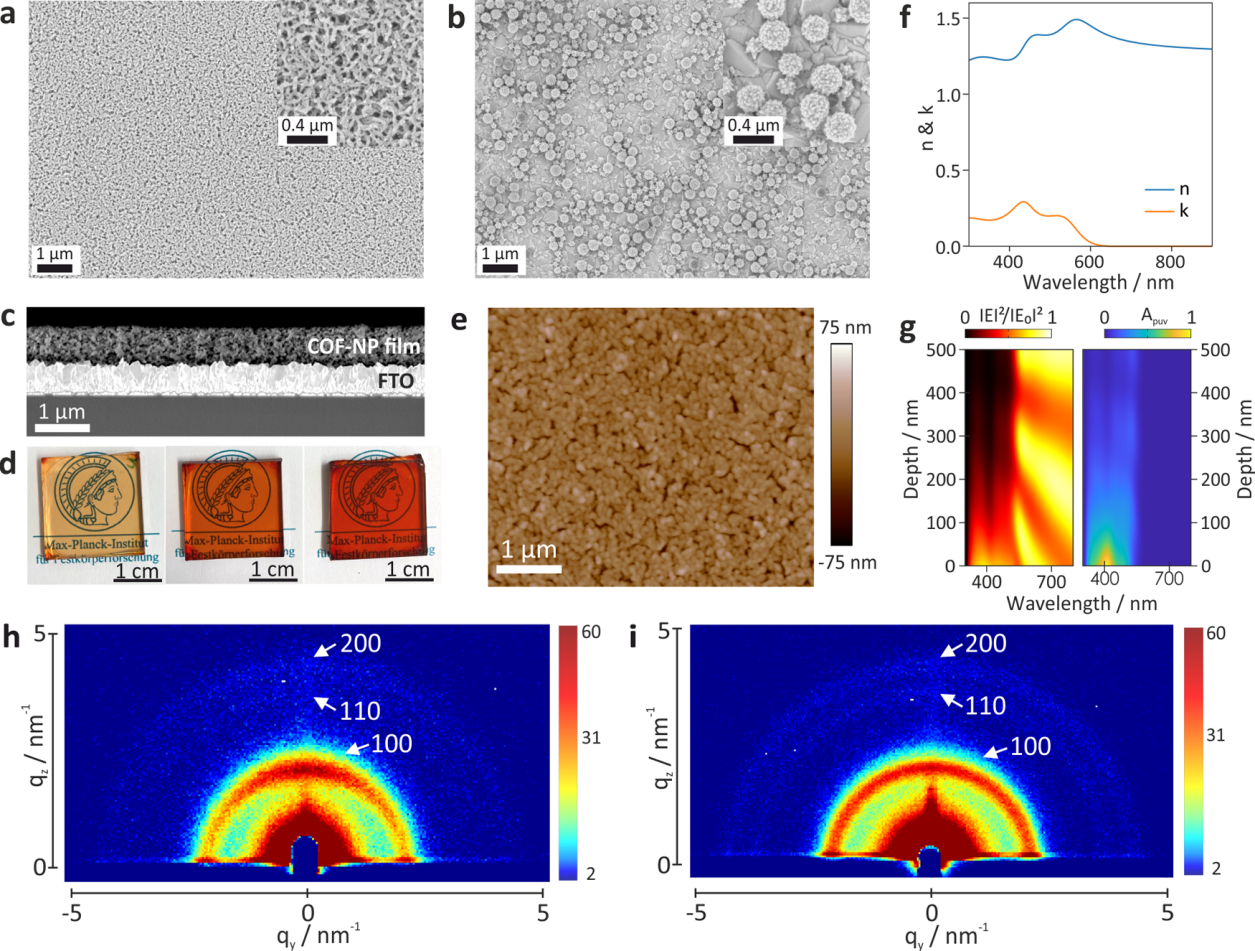
Morphology of the solution-processed films from TPB-MeOTP-NP and TPB-MeOTP-NS. (a, b) Top–down SEM morphology of spin-coated TPB-MeOTP-NP (a) and TPB-MeOTP-NS (b) COF films. (c) Cross sectional SEM image of FTO/TPB-MeOTP-NP with 20 spin-coating cycles. COF-NP denotes TPB-MeOTP-NP. (d) Photographs of FTO/TPB-MeOTP-NP with 2 cycles (left), 10 cycles (middle), and 20 cycles (right). The substrates are placed on top of the logo of the Max Planck Institute for Solid State Research (MPI-FKF) to demonstrate the transparency of the films. Permission granted by MPI-FKF. (e) AFM height image of TPB-MeOTP-NP films (20 cycles). (f) Real (*n*) and imaginary (*k*) parts of the refractive index found for TPB-MeOTP-NP films with the structure of air/glass/indium tin oxide (ITO)/COF/air, retrieved by ellipsometry modeling. (g) Calculated spatial and spectral distribution of the normalized electric field intensity (left) and normalized absorption per unit volume map (*A*_puv_, right) across the section of a 500-nm-thick TPB-MeOTP-NP film. The ITO/TPB-MeOTP-NP interface is situated at 0 nm on the vertical axis. The system is illuminated from the glass substrate side. (h, i) Grazing-incidence wide-angle X-ray scattering two-dimensional (GIWAXS 2D) patterns of spin-coated TPB-MeOTP-NP (h) and TPB-MeOTP-NS (i) films (20 cycles) on a SiO_2_/Si wafer.

To further understand the self-assembly behavior difference of the terephthalaldehyde linkers, pulsed-field gradient NMR (PFG NMR) measurements were performed for MeOTP and TP at various concentrations in acetonitrile, providing the diffusion coefficients of MeOTP and TP in acetonitrile (Supporting method, Figure S30). According to the Stokes–Einstein equation, changes in the diffusion coefficient can be used to study the molecular aggregation, causing an increase in the apparent hydrodynamic radius.^39^ The results reveal that although both MeOTP and TP self-assemble in concentrated solution (∼6–12 mM), indicated by the increasing diffusion coefficients with decreasing concentration, the MeOTP linker remains self-assembled even in more dilute solution (∼1.5 mM), demonstrating a stronger tendency to self-assemble in acetonitrile. Therefore, in the crystallization process of TPB-MeOTP, the MeOTP linker self-assembly could offer a template effect, leading to faster crystallization in line with the larger *k*_p_ for TPB-MeOTP. Besides, we also note that MeOTP has a significantly lower solubility in acetonitrile (∼2 mg mL^–1^) than TP and MeTP (>80 mg mL^–1^), further implying the strong self-assembly of MeOTP in acetonitrile. Considering that the TPB-MeOTP colloid reaction combines linker template-induced crystallization with fast reaction kinetics, it is plausible to speculate that TPB-MeOTP nanoplates are formed by the preferential particle growth along the interlayer stacking direction, which is also inferred by the film crystallinity property below (Figure 5h). On the other hand, the formation of TPB-MeOTP-NS could originate from nonoriented agglomeration of crystallites occurring during the polymerization at high linker concentrations (Supporting Figure S31), as an increased linker concentration leads to a larger *k*_p_ (Supporting Figure S32).

As solution-processed nanoparticle thin films have been extensively applied in solar-energy-conversion devices,^21,22,24^ we envisaged the application of TPB-MeOTP nanoparticles in nanofilm-based devices. To this end, we first investigated and compared the film morphology prepared by the classic spin-coating technique for TPB-MeOTP-NP and TPB-MeOTP-NS. We observed that TPB-MeOTP-NP furnishes continuous and homogeneous nanofilms on the scale of centimeters on fluorine-doped tin oxide (FTO) substrates (Figure 5a,d, and Supporting Figure S33). The film thickness can be easily tuned by adjusting the spin-coating cycles from ∼120 nm with 4 cycles to ∼660 nm with 20 cycles (Figure 5c, and Supporting Figure S34). Meanwhile, based on the AFM images in Figure 5e and Supporting Figure S35, the roughness of the spin-coated films from 2, 4, and 20 cycles was determined to be 14.6, 14.5, and 13.1 nm, respectively, indicating no obvious morphology change with respect to spin-coating cycles. We also note that the TPB-MeOTP-NP film has excellent mechanical properties on the FTO substrate, suggested by very limited delamination after sonicating the film in aqueous electrolytes (Supporting Figure S37). Besides using FTO as the substrate, high-quality TPB-MeOTP-NP spin-coated films can also be obtained on other substrates, such as glass or SiO_2_/Si wafers (Supporting Figure S36). In comparison, spin coating of TPB-MeOTP-NS leads to discontinuous coagulates, and the film quality could not be improved by increasing coating cycles (Figure 5b and Supporting Figure S38). The results imply that TPB-MeOTP-NP is superior for the preparation of nanofilms compared to TPB-MeOTP-NS, likely due to the nanoplate shape offering enhanced interaction and contact area between particles and the substrate. Favored by the high-film quality, the optical constants of the TPB-MeOTP-NP film, important for the design of optoelectronic devices to maximize light harvesting and charge collection, were obtained by ellipsometry. The complex refractive index spectra, comprised of the real (*n*) and imaginary (*k*) parts, are displayed in Figure 5f. The spectral features in the imaginary part of the refractive index are in good agreement with the absorption spectra (Supporting Figure S43). This result allows us to further calculate the spatial distribution of the square magnitude of the electric field and consequently the absorption per unit volume (*A*_puv_) along the cross section of the TPB-MeOTP-NP film (Figure 5g). It can be seen that the light absorption of the TPB-MeOTP-NP film is intensive in the thickness range from 0 to ∼150 nm, while it quickly vanishes with further increasing film thickness (Supporting Figure S39). Combining the ellipsometry data and the fact that charge collection efficiency could decrease significantly with increasing thickness, the optimal film thickness for solar energy conversion is likely to be around 150 nm. The crystallinity of TPB-MeOTP-NP and TPB-MeOTP-NS films was measured by grazing-incidence wide-angle X-ray scattering (GIWAXS, Figure 5h,i), where both films show reflection peaks with *q* values of 2.1, 3.7, and 4.3 nm^–1^ corresponding to 100, 110, and 200 facets, respectively. Nevertheless, TPB-MeOTP-NP films clearly exhibit stronger reflection intensity in the out-of-plane direction (*q*_z_), revealing that a large fraction of TPB-MeOTP-NP shows a pore channel parallel to the substrate plane (Supporting Figure S41). Considering that SEM of the TPB-MeOTP-NP film (Figure 5a) indicates a preferential growth direction of the nanoplates parallel to the substrate plane, it can be inferred that TPB-MeOTP interlayer π–π interactions direct the particle growth, supporting our hypothesis of nanoplate formation discussed above. In contrast, TPB-MeOTP-NS films show no preferential intensity with respect to the reflection direction, consistent with spherical nanoparticle shape.

**Figure 6:**
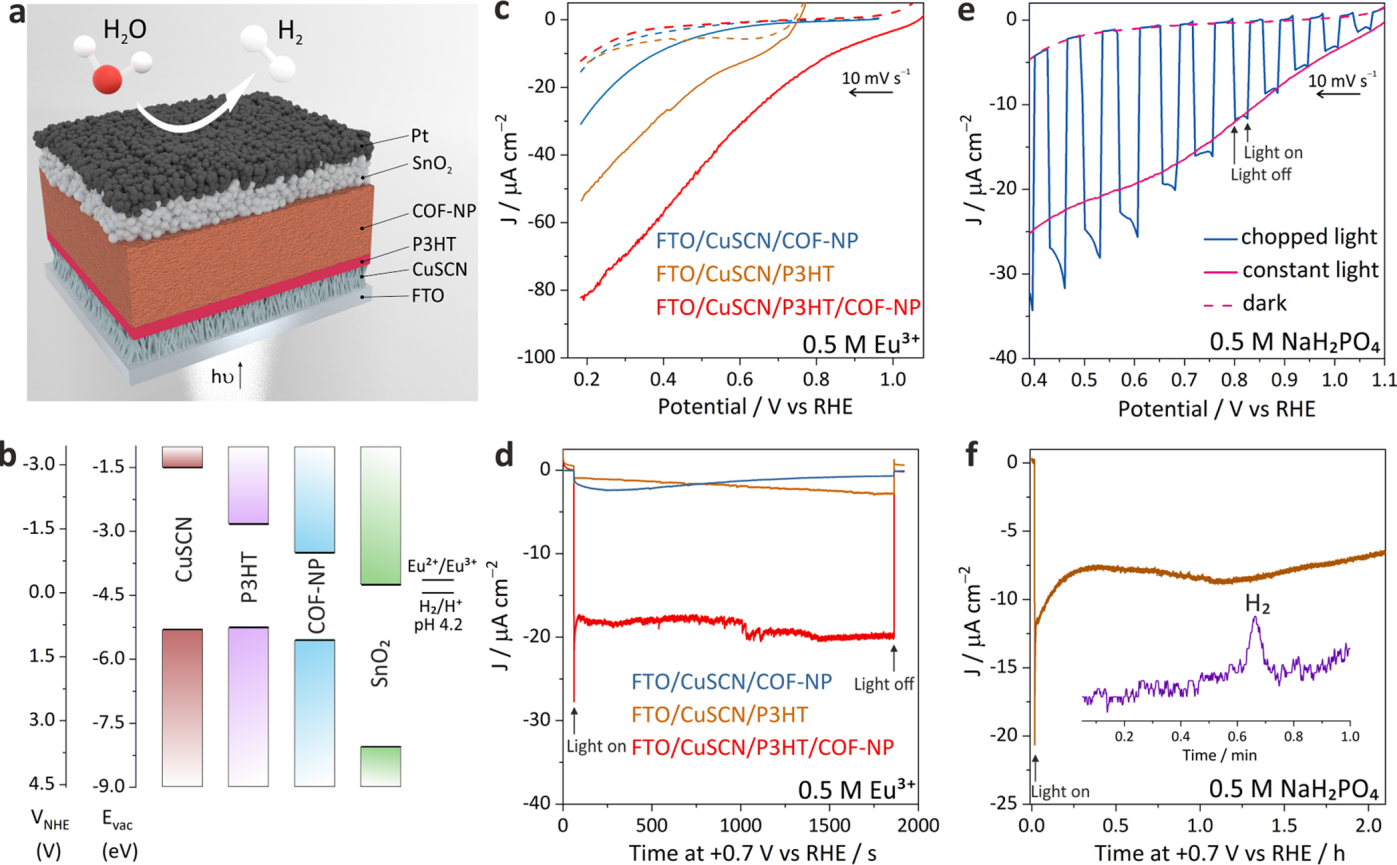
Photoelectrochemical characterization. (a) Schematic of the optimized hydrogen evolution COF photocathode layer arrangement. (b) Energy levels of the components in the photocathode vs vacuum and normal hydrogen electrode (NHE), including CuSCN, P3HT, TPB-MeOTP, and SnO_2_. The energy levels of SnO_2_ and H_2_/H^+^ were adapted at pH 4.2 given their Nernstian behavior.^40^ (c, d) LSV (c) and CA (d) of TPB-MeOTP-NP, P3HT, and P3HT/TPB-MeOTP-NP photocathodes in 0.5 M Eu^3+^ aqueous electrolytes. (e, f) LSV (e) and CA (f) of the optimized COF-based photocathode with the structure of CuSCN/P3HT/TPB-MeOTP-NP/SnO_2_/Pt in the 0.5 M NaH_2_PO_4_ aqueous electrolyte. A representative GC trace of evolved hydrogen is shown in the inset graph of (f). COF-NP denotes TPB-MeOTP-NP.

Having investigated the morphology of TPB-MeOTP nanoparticle films, we next sought to apply them as photocathodes in PEC hydrogen evolution. The highest occupied molecular orbital (HOMO) and the lowest unoccupied molecular orbital (LUMO) of TPB-MeOTP are estimated to be −5.6 and −3.5 eV, respectively, vs vacuum by electrochemical cyclic voltammograms (Supporting Figure S42), which are thermodynamically suitable for the hydrogen evolution reaction. The electrochemical band gap (2.1 eV) is in good agreement with that obtained from Tauc plot analysis (2.1 eV, Supporting Figure S43). TPB-MeOTP film-based photocathodes were first evaluated in conjunction with a sacrificial agent whose reduction is kinetically facile, such that the photocurrent loss due to the sluggish kinetics of the hydrogen evolution and interfacial charge extraction between the COF film and the hydrogen evolution reaction (HER) catalyst layer can thus be ignored. In this work, we employed the classic Eu^2+/3+^ couple as the sacrificial agent since the Eu^2+/3+^ redox potential is located at a more negative position relative to hydrogen evolution, representing a thermodynamically less favorable process.^41,42^ Therefore, the photogenerated electrons enabling the reduction of Eu^3+^ are also thermodynamically capable to reduce protons and produce hydrogen. As shown by linear sweep voltammetry (LSV) (Supporting Figure S44), the photocathodes prepared by directly spin coating 4 cycles TPB-MeOTP-NP on FTO glass clearly exhibit photocathodic current under 1 Sun illumination, starting from around +0.9 V against the reversible hydrogen electrode (*V*_RHE_), establishing the viability to generate solar-driven electrons by TPB-MeOTP-NP films. Nevertheless, the photocathodic current of the TPB-MeOTP-NP photocathode increases sluggishly with scanning to more negative potentials and reaches a photocurrent density (*J*_ph_) of 2 μA cm^–2^ only at +0.59 V_RHE_ (the potential required to obtain 2 μA cm^–2^ is defined as onset potential for comparison, *V*_on_), implying severe photogenerated carrier recombination. To provide solutions to improve charge separation and advance the application of COFs in photoelectrochemistry, the effects of introducing a hole-transport layer (HTL) and constructing a heterojunction on photocurrent response were investigated, as shown in the device structure (Figure 6a,b). Electrochemically deposited CuSCN nanowires^43,44^ (Supporting Figure S45) were used as HTL due to their facile preparation, high hole conductivity, and suitable energy level alignment with TPB-MeOTP. Full details and discussions related to device structure optimization are shown in Supporting Figures S46–S48. It can be seen that constructing a heterojunction consisting of TPB-MeOTP-NP (∼120 nm) and a 10-nm-thick donor polymer, poly(3-hexylthiophene-2,5-diyl) (P3HT),^45^ positively shifts the *V*_on_ in the presence or absence of CuSCN HTL (Supporting Figure S47), indicating that P3HT/TPB-MeOTP-NP heterojunction enhances charge separation. In the optimal condition with CuSCN as HTL (CuSCN/P3HT/TPB-MeOTP-NP), the *V*_on_ is positively shifted to +1.03 V_RHE_ (Figure 6c). The performance enhancement is also reflected by chronoamperometry (CA) measurement. CuSCN/P3HT or CuSCN/TPB-MeOTP-NP only shows a negligible *J*_ph_ of 2 and 3 μA cm^–2^ at +0.7 V_RHE_ (Figure 6d), while CuSCN/P3HT/TPB-MeOTP-NP exhibits a *J*_ph_ of 21 μA cm^–2^ (17 times higher than FTO/TPB-MeOTP-NP) with an excellent stability over 30 min. It is noteworthy that the performance of CuSCN/P3HT/TPB-MeOTP-NP heterojunction photocathodes is reproducible as shown by LSV and CA of multiple samples (Supporting Figure S49), suggesting that the photocathode architecture presented here is an effective approach to enhance charge separation in COFs. The origin of the performance improvement is the thermodynamically more favorable transfer of photogenerated holes to the P3HT donor polymer layer at the interface of the P3HT/TPB-MeOTP-NP heterojunction, which helps to suppress charge recombination in the COF. We also prepared a photocathode based on TPB-MeOTP-NS with the optimized structure (CuSCN/P3HT/TPB-MeOTP-NS), which shows a lower *J*_ph_ of 12 μA cm^–2^ at +0.7 V_RHE_ and a negatively shifted *V*_on_ of ∼+0.89 V_RHE_ (Supporting Figure S50). The lower performance of the TPB-MeOTP-NS photocathode is mainly attributed to the poor film quality of TPB-MeOTP-NS.

Further studies toward implementing solar-driven hydrogen evolution were carried out. To provide catalytically active sites for the hydrogen evolution reaction, Pt nanoparticles as HER catalyst overlayer were integrated with TPB-MeOTP photocathodes (Supporting Figure S51). The solar-driven hydrogen evolution of TPB-MeOTP-NP photocathodes was measured under 1 Sun illumination in 0.5 M NaH_2_PO_4_ as electrolyte, which provides the same pH as 0.5 M Eu^3+^ aqueous electrolyte and therefore a comparable condition to analyze the carrier recombination. LSV measurements indicate that the Pt overlayer remarkably shifts the *V*_on_ from +0.93 V to +1.07 V_RHE_ (Supporting Figure S52), offering an almost identical *V*_on_ with Eu^3+^ reduction. Since the Pt overlayer does not change the hole extraction at the FTO side and charge transport in the P3HT/TPB-MeOTP-NP heterojunction, the positive *V*_on_ shift suggests a reduced photogenerated electron recombination at the photoelectrode surface. While a noticeably enhanced dark current can be observed at +0.4 V_RHE_ for CuSCN/P3HT/TPB-MeOTP-NP/Pt in 0.5 M NaH_2_PO_4_, introducing a SnO_2_ layer on top of TPB-MeOTP-NP as the electron-collecting layer^46,47^ reduces the dark current and further increases the *J*_ph_ (Figure 6e). The optimum photocathode with the structure of CuSCN/P3HT/TPB-MeOTP-NP/SnO_2_/Pt results in a *V*_on_ of +1.06 V_RHE_ and a *J*_ph_ of 17 μA cm^–2^ at +0.7 V_RHE_, representing a new benchmark for reported COF photocathodes (Supporting Table S5). CA characterization demonstrates that the optimum photocathode can be continuously operated at +0.7 V_RHE_ for over 2 h, indicating a fairly good stability (Figure 6f and multiple samples shown in Supporting Figure S53). Meanwhile, the production of hydrogen was detected during a CA test by using gas chromatography (Figure 6f and Supporting Figure S54). The P3HT/TPB-MeOTP-NP heterostructure, enabled by spin coating of colloidal COFs, demonstrates the feasibility to improve the performance of COF photoelectrodes by engineering multilayer photoelectrode structures, thus highlighting the promise of colloidal COFs for PEC applications. It can be envisaged that using bulk-heterojunction structures to afford an increased number of interfaces for charge separation compared to the bilayer structure shown here could further boost *J*_ph_. Indeed, developing donor polymer:TPB-MeOTP-NP or TPB-MeOTP-NP:inorganic nanoparticle bulk-heterojunction structure-based COF photoelectrodes^40,48^ is currently ongoing in our laboratory. Nevertheless, it is noteworthy that only a few photocathodes, including both organic and inorganic semiconductor-based ones, have so far been reported with a similarly positive onset potential (>+1 V_RHE_).^49−52^ Our results thus indicate that COF photoelectrodes hold great promise for tandem devices^53^ to achieve unbiased solar-to-fuel conversion, especially after gaining a higher *J*_ph_ with further optimization.

## Conclusions

In summary, we have demonstrated the fabrication of COF photoelectrodes for solar hydrogen evolution by solution processing of COF colloids. By analyzing the structure, morphology, nucleation behavior, and growth kinetics of the COF colloids, we draw a comprehensive picture of the shape-anisotropic growth of the COF nanoparticles, which is attributed to a preferential growth along the interlayer stacking direction. The as-obtained crystalline colloidal COF nanoplates TPB-MeOTP-NP show excellent colloidal stability of up to 10 months. Kinetic studies of the crystallization process and particle growth for a series of terephthalaldehyde linkers suggest that methoxy functionalization and the associated self-assembly behavior plays a key role in the formation of nanoplate particles. Indeed, PFG NMR analysis illustrates that the MeOTP linker shows a stronger tendency to self-assemble in acetonitrile than TP, enabling a template-induced crystallization for TPB-MeOTP as well as fast and high-yield colloid formation. Moreover, TPB-MeOTP-NP exhibits a significant advantage in preparing smooth, centimeter-scale homogeneous, and thickness-controlled nanofilms, compared to TPB-MeOTP-NS. The photoelectrodes fabricated from TPB-MeOTP colloids show photocathodic current under illumination for PEC Eu^3+^ reduction. By introducing CuSCN nanowires as HTL and a P3HT/TPB-MeOTP-NP heterojunction to suppress charge carrier recombination, the *V*_on_ of COF photocathodes was positively shifted from +0.59 V to +1.03 V_RHE_. Moreover, the *J*_ph_ at +0.7 *V*_RHE_ reached 21 μA cm^–2^ with an excellent stability over 30 min continuous illumination. Coupled with an electron-collecting SnO_2_ layer and a Pt HER catalyst layer, an optimized COF photocathode furnished an exceptionally positive *V*_on_ of +1.06 *V*_RHE_ for PEC hydrogen evolution, among the best results of classical semiconductor-based photocathodes, and a stable *J*_ph_ over 2 h. Given the fact that only a limited number of semiconductor materials have been realized so far with a *V*_on_ > +1 *V*_RHE_, our results bode well for the use of COFs as a new generation of polymeric semiconductors for photoelectrodes. More generally, the high-quality nanofilm preparation and photoelectrode design presented herein advance our understanding of the subtleties of photoelectrode preparation and the functional interplay between the individual components, thus paving the way for the application of COFs in next-generation semiconductor devices.

## Supporting Information Available

Full description of the experimental methods, COF synthesis, and supplementary methods, all of the supporting Figures S1–S55, and the supporting Tables S1–S5 mentioned in the main text (PDF)
