# Supplementary material for: Covalent Organic Framework Nanoplates Enable Solution-Processed Crystalline Nanofilms for Photoelectrochemical Hydrogen Evolution
Source: J Am Chem Soc. 2022 Jun 3;144(23):10291–300. doi: 10.1021/jacs.2c01433 (PMC9204765; doi:10.1021/jacs.2c01433)
Supplement: Supplementary file 1 — ja2c01433_si_001.pdf [file ja2c01433_si_001.pdf]

Supplementary Information

Covalent Organic Framework Nanoplates Enable Solution Processed Crystalline Nanofilms for Photoelectrochemical Hydrogen Evolution

Liang Yao[1], Andrés Rodríguez-Camargo[1,6], Meng Xia[2], David Mücke[3], Roman Guntermann[4], Yongpeng Liu[5], Lars Grunenberg[1,7], Alberto Jiménez-Solano[1], Sebastian T. Emmerling[1,7], Viola Duppel[1], Kevin Sivula[5], Thomas Bein[4,8], Haoyuan Qi[3,9], Ute Kaiser[3], Michael Grätzel[2], Bettina V. Lotsch[1,6,7,8*]*

[1]Nanochemistry Department, Max Planck Institute for Solid State Research, Heisenbergstraße 1, 70569 Stuttgart, Germany

[2]Laboratory of Photonics and Interfaces, Station 6, École Polytechnique Fédérale de Lausanne, 1015 Lausanne, Switzerland

[3]Central Facility for Materials Science Electron Microscopy, Ulm University, 89081 Ulm, Germany

[4]Department of Chemistry and Center for NanoScience (CeNS), Ludwig-Maximilians-Universität München, Butenandtstraße 5-13 (E), 81377 Munich, Germany

[5]Laboratory for Molecular Engineering of Optoelectronic Nanomaterials, Station 6, École Polytechnique Fédérale de Lausanne (EPFL), 1015 Lausanne, Switzerland

[6]Department of Chemistry, University of Stuttgart, Pfaffenwaldring 55, 70569 Stuttgart, Germany

[7]Department of Chemistry, Ludwig-Maximilians-Universität München, Butenandtstraße 5-13, 81377 Munich, Germany

[8]E-conversion and Center for Nanoscience, Lichtenbergstraße 4a, 85748 Garching bei München, Germany

[9]Center for Advancing Electronics Dresden (cfaed) and Faculty of Chemistry and Food Chemistry, Technische Universität Dresden, 01062 Dresden, Germany

[*]Corresponding authors

E-mail: l.yao@fkf.mpg.de; b.lotsch@fkf.mpg.de

Contents

1. Experimental methods 3

Chemicals 3

Characterization 3

2. Synthesis 6

Procedures for COF particle Synthesis..6

Supplementary Table 1. The chemical amount for the reactions with varying [TAPB] 7

Reactions for time-dependent PXRD 7

Supplementary Table 2. The chemical amount of the reactions for time-dependent PXRD.................8

In-situ **^{1}H-NMR characterizations**..8

Supplementary Table 3. The stock solutions for *in-situ* ^{1}H-NMR characterizations 8

Solvothermally synthesized TPB-MeOTP 8

3. Supplementary Methods 9

Ellipsometry modeling 9

Supplementary Table 4. Dielectric function parameters of the best fitting..10

Solubility estimation 10

PFG NMR characterization..10

4. Supplementary Figures 12

Supplementary Table 5. Comparison of reported COF photocathodes. 61

5. Supplementary References 62

1. Experimental methods

Chemicals

2,5-dimethoxyterephthalaldehyde (MeOTP, Sigma-Aldrich, 97%), terephthalaldehyde (TP, Sigma-Aldrich, 99%), 2,5-dimethylterephthalaldehyde (MeTP, Sigma-Aldrich, 95%), 2,5-Dihydroxyterephthalaldehyde (HTP, Sigma-Aldrich, 95%), 1,3,5-tris(4-aminophenyl)benzene (TAPB, TCI, >93%), scandium(III) triflate ($Sc(OTf)_3$, Sigma-Aldrich, 99%), acetonitrile (Carl Roth, ≥99.5%), dichloromethane (Merck, ≥99.8%), poly(3-hexylthiophene-2,5-diyl) (P3HT, Sigma-Aldrich, regioregular, average Mw 20k-45k), Nafion 117 solution (Sigma-Aldrich, 5% in a mixture of lower aliphatic alcohols and water), Pt nanoparticle dispersion (3 nm particle size, Sigma-Aldrich), CD_3CN (Sigma-Aldrich, ≥99.8 atom % D), $CDCl_3$ (Sigma-Aldrich, ≥99.8 atom % D), $CuSO_4$ (Carl Roth, ≥99%), KSCN (Carl Roth, ≥98.5%), Ethylenedinitrilotetraacetic acid (EDTA, AppliChem, ≥99.4%), SnO_2 (Alfa Aesar, 15% in H_2O colloidal dispersion) were used as received.

Characterization

Atomic force microscopy. Atomic force microscopy (AFM) was performed using Asylum Research MFP-3D, equipped with a micro cantilever (OMCL-AC160TS-R3). TPB-MeOTP-NP and TPB-MeOTP-NS were deposited on SiO_2/Si wafers by drop cast, and TPB-MeOTP nanofilms were prepared by spin coating as described in the film preparation part.

Nuclear magnetic resonance. ^{1}H-NMR spectra of the COF linkers and *in-situ* ^{1}H-NMR spectra of the colloid reactions were recorded on a JEOL ECZ 400S 400 MHz spectrometer. ^{1}H-PFG NMR diffusion measurements were performed in 5 mm tubes on a Bruker Avance III 400 MHz spectrometer at 300 K with a stimulated-echo sequence and default spoiler gradient (*diffSte* program, Bruker TopSpin).

Scanning electron microscopy. SEM images were measured with a Zeiss Merlin under the electron high tension voltage of 1.5 kV. 3 nm of Ir was sputtered on the samples in **Fig. 5a,b**, **and Supplementary Fig. 33** and **Fig. 36** by Leica ACE600.

FT-IR spectroscopy. FT-IR measurements were carried out on a PerkinElmer Spectrum Two in attenuated total reflection (ATR) geometry equipped with a diamond crystal.

Super critical CO_2 activation. Super critical CO_2 activation was performed on a Leica EM CPD300 critical point dryer. Prior to super critical CO_2 activation, the COF samples were soaked in methanol.

Transmission electron microscopy. HRTEM characterizations were performed on an image-side spherical-aberration-corrected FEI Titan 80-300 operated under 300 kV. In order to reduce electron irradiation damage, low-dose conditions have been applied. SAED patterns were recorded with a typical dose of c.a. 2 $e^-/Å^2$, whereas the total dose for HRTEM imaging was limited to c.a. 70 $e^-/Å^2$.

Sorption Measurements. N_2 sorption were measured with a Quantachrome Instruments Autosorb iQ 3 at 77 K. The pore size distribution (PSD) was determined with the calculation model of N_2 at 77 K on carbon using the QSDFT (cylindrical pores, adsorption branch) in ASiQwin software v 3.01. The COF samples were activated in high vacuum at 120 °C for 12 h before measurement. A suitable pressure region (P/P_0 = 0.05–0.2) was chosen for the BET surface area determination.

Grazing-incidence wide angle X-ray scattering. Two-dimensional grazing-incidence wide angle X-ray scattering (GIWAXS) data were recorded with an Anton Paar SAXSpoint 2.0 system equipped with a

Primux 100 micro Cu K_α source and a Dectris EIGER R 1M 2D detector. The COF films were positioned at a sample-detector distance of 140 mm and were measured with an incidence angle of 0.2°.

Ellipsometry. The complex refractive index of the TPB-MeOTP-NP film was obtained from ellipsometry measurements at room temperature (Sopra PS-1000 SAM).

Dynamic Light Scattering analysis. DLS measurements were carried out on a Malvern Zetasizer Nano ZS. The COF colloid was diluted with pure acetonitrile prior to DLS measurements.

Cyclic voltammetry measurements. The cyclic voltammetry measurements for determining HOMO and LUMO energy levels of P3HT and TPB-MeOTP COF were carried out on either an Autolab potentiostat or a WaveDriver 200 EIS Bipotentiostat. A three-electrode configuration was employed, with Ag/Ag^+ as reference electrode, Pt wire as counter electrode, and glassy carbon or carbon paper as working electrode. Anhydrous acetonitrile, containing 0.1 M tetrabutylammonium hexafluorophosphate, was used as supporting electrolyte. All the measurements were performed with the scan rate of 50 mV s^{-1}, under Ar atmosphere. The potential was calibrated against ferrocene/ferrocenium redox couple (−5.10 eV versus vacuum level).

UV-vis spectroscopy. UV-vis spectra of P3HT and TPB-MeOTP films were recorded on a Cary 60 UV-Vis Spectrophotometer.

Powder X-ray diffraction. Powder X-ray diffraction (PXRD) patterns were recorded in Debye-Scherrer geometry at room temperature, on a Stoe Stadi P diffractometer (Cu-$K_{\alpha 1}$) equipped with a Ge(111) primary monochromator. All the samples were sealed in 1.0 mm glass capillaries, and measured with spinning for improving particle statistics.

Structure modeling. The modeling of TPB-MeOTP COFs, as well as the Pawley refinement of the COF structures, was performed with BIOVA Materials Studio 2017 (17.1.0.48. Copyright © 2016 Dassault Systèmes) suite. The geometry and the unit cell were optimized using force fields (Forcite, universal force fields with Ewald electrostatic and van der Waals summations method). To obtain the FWHM values of 100 peaks, the experimental PXRD profiles were fitted with a Gaussian function for 100 diffraction and a cubic polynomial function for subtracting the background. Representative fittings are shown in **Supplementary Fig. 18**.

TPB-MeOTP film fabrication. TPB-MeOTP-NP and TPB-MeOTP-NS inks used for spin-coating were prepared by concentrating TPB-MeOTP-NP or TPB-MeOTP-NS reaction solution with a Heidolph rotary evaporator, under 115 mbar at 35 °C. No particle agglomeration or obvious crystallinity change was observed during this process. The mass concentration of the inks was 4.5 mg mL^{-1} and the digital images were displayed in **Fig. 1**. TPB-MeOTP-NP and TPB-MeOTP-NS were typically deposited on FTO substrates or other substrates by spin-coating the inks at 1500 rpm for 60 s (unless specifically stated otherwise). Multi coating cycles were performed with the same procedure. The resulting films were then post-annealed at 80 °C for 15 min in the air.

Photoelectrode fabrication. The FTO-coated glass substrates (Sigma-Aldrich, ~7 Ω sq^{-1}) were subjected to a sequential sonication with deionized water (20 min, 5 times), acetone (20 min) and isopropanol (20 min), followed by drying with compressed air. CuSCN nanowire electrodeposition was carried out following to the procedure in the literature.[1] The precursor solution was prepared by first stirring the mixture of $CuSO_4$ (12 mM) and EDTA (12 mM) in deionized water for 10 min, and then adding KSCN (12 mM) and stir the resulting solution for overnight. The electrodeposition was performed using a three-electrode configuration with FTO substrate as working electrode, Pt as counter electrode and Ag/AgCl as reference

electrode. The deposition potential was –0.3 V vs Ag/AgCl and the deposition time was 60 s. P3HT layer was prepared by spin coating 1 mg mL^{-1} P3HT solution in chloroform at 1500 rpm for 60 s, followed by a thermal annealing at 80 °C for 10 min in the air. Due to the hydrophobic surfaces of P3HT film, the first coating of TPB-MeOTP-NP or TPB-MeOTP-NS was at 6500 rpm, which is useful for achieving smooth films in the following process. This high spin-rate coating was not counted in the cycles as it only provides a low particle coverage on P3HT surface. TPB-MeOTP-NP or TPB-MeOTP-NS was then deposited as described above. A diluted SnO_2 solution (2.5 wt%) was used for SnO_2 layer fabrication[2], prepared by adding water in the pristine SnO_2 colloid (15 wt%). The diluted SnO_2 was spin-coated at 1500 rpm for 60 s onto TPB-MeOTP-NP. The resulting films were transferred into a vacuum oven at 60 °C and annealed for 30 min. Pt catalyst overlayer was prepared by adding 16 µL Nafion 117 solution in 1 mL Pt nanoparticle dispersion as binder, and then spin-coated the solution onto COF films or SnO_2 at 1500 rom for 60 s. After the coating, the films were annealed at 80 °C for 15 min to remove the solvent residual.

Photoelectrochemical measurement. Photoelectrochemical measurements were carried out in a conventional three-electrode configuration powered by either an Autolab potentiostat or a WaveDriver 200 EIS Bipotentiostat. The COF photocathode was used as the working electrode with an active area of 0.283 cm^2, and an Ag/AgCl (saturated KCl) electrode and a Pt wire were employed as reference electrode and counter electrode, respectively. Simulated 1 sun illumination (AM 1.5G, 100 mW cm^{-2}) was provided by a Sciencetech solar simulator (XLH-E-500X) and calibrated by a Thorlabs photodiode (PM100D). PEC performance evaluation was performed by illuminating from the substrate side. Electrolyte (0.5 M) was prepared by dissolving $Eu(NO_3)_3$ or NaH_2PO_4 in Nano-pure system purified water with the resistivity of 18.2 MΩ cm. The electrolyte pH was determined by a pH meter, calibrated with standard pH buffers. All experiments were carried out at ambient temperature and electrode potentials were converted to RHE scale using $E_{RHE} = E_{Ag/AgCl} + 0.059 \times pH + 0.197$. Hydrogen evolution photocathodes was measured under Ar atmosphere, and the electrolyte was purged with Ar for 15 min prior to the measurements.

Solar hydrogen evolution detection. A gas-tight single compartment polyether ether ketone (PEEK) electrochemical cell with 3-electrode configuration was used for gas chromatography (GC) measurements. Carrier gas Helium (99.9999%, Carbagas) was continuously infused into the cell with the flow rate of 10 mL min^{-1} (controlled by a digital thermal mass flow controller, Bronkhorst High-Tech, F201CV). The COF photocathode was illuminated from the substrate side with an applied potential of +0.7 V_{RHE} for solar hydrogen production. A Newport LCS-100 solar simulator with AM1.5G filter was used as light source. Gas product was separated by a micropacked ShinCarbon column (Restek) and was analyzed by a pulse discharge detector (PDD, Vici) in an online GC (Trace ULTRA, Thermo). The control GC test without performing PEC measurement was taken after purging the electrochemical cell with Helium for 3 hours. The hydrogen retention time was calibrated using standard calibration gas (Carbagas AG), as shown in **Supplementary Fig. 55**. The COF photocathode with the working area of 0.6 cm^2 was operated at +0.7 V_{RHE} and illuminated for 1697 s (a 10 s light chopping was performed at 14 s). The gas in headspace was sampled and analyzed for three times (**Supplementary Fig. 54**) to determine the hydrogen evolution from the COF photocathode.

2. Synthesis

Procedures for COF particle Synthesis

General procedures for COF synthesis. COF colloids including TPB-MeOTP, TPB-MeTP, TPB-TP were synthesized via a modified reported procedure.[3] In brief, MeOTP (or other terephthalaldehyde linkers) was firstly dissolved in dichloromethane and acetonitrile mixture. TAPB, with the molar ratio of 1:1 between amine groups in TAPB and aldehyde groups in terephthalaldehyde linkers, was then added in the solution. $Sc(OTf)_3$ with the amount of 0.08 equivalent of amine groups in TAPB, dissolved in acetonitrile, was injected into the reaction mixture via a syringe in 5 min. All the colloid reactions were performed with stirring at room temperature. The representative TPB-MeOTP-NP and TPB-MeOTP-NS were synthesized with [TAPB] of 1.97 mM and 5.02 mM, respectively. Solvothermally synthesized TPB-MeOTP was prepared according to the reported procedure[4].

TPB-MeOTP-NP. 2,5-dimethoxyterephthalaldehyde (MeOTP, 17.2 mg, 88.6 μmol) and dichloromethane (1 mL) were placed in a Biotage 20 mL high precision glass vial and sonicated for 10 s. Acetonitrile (27 mL) was added in the vial, and the resulting solution was stirred at room temperature until MeOTP was dissolved. Then 1,3,5-tris(4-aminophenyl)benzene (TAPB, 20.8 mg, 59.2 μmol) was added in the vial. The vial was sealed with a cap and stirred at room temperature. $Sc(OTf)_3$ (7 mg, 14.2 μmol), 0.08 equivalent per amine functional group in TAPB, was dissolved in 2 mL acetonitrile and injected in the reaction tube within 5 min. The reaction was stirred at room temperature for 20 hours. For the reaction kinetic study, the reaction time was specified and controlled by a timer. The resulting particle solution was directly used for DLS and SEM characterization after dilution. To collect the particle powder for PXRD, BET, TEM and FT-IR measurements, 1 M NaCl aqueous solution (200 μL) was added in the vial to precipitate out the particles. The precipitates were collected with a filter paper, washed with acetone, dimethylformamide, chloroform and methanol, and subjected to Soxhlet extraction with methanol for 12 h. The final product (30.6 mg, yield: 87.9%) was obtained after activating with supercritical CO_2. In the whole workup process, the precipitates remained wet prior to the super critical CO_2 activation.

TPB-MeOTP-NS. Following the procedure of TPB-MeOTP-NP, MeOTP (17.2 mg, 88.6 μmol) dichloromethane (0.35 mL), acetonitrile (9.45 mL), TAPB (20.8 mg, 59.2 μmol), $Sc(OTf)_3$ (7 mg, dissolved in 2 mL) were used for the synthesis. The reaction yield was 90.2% (31.4 mg).

TPB-MeOTP, TPB-MeTP and TPB-TP colloid reactions with varying [TAPB]. Following the procedure of TPB-MeOTP-NP, [TAPB] was varied from 5.02 mM to 0.52 mM, while the ratio of amine groups in TAPB and aldehyde groups in terephthalaldehyde linkers and $Sc(OTf)_3$ amount were fixed at 1:1 and 0.08 equivalent, respectively. After the reaction, the colloids were precipitated out with 1 M NaCl aqueous solution. The precipitates were collected via filtration, washed with acetone, dimethylformamide, chloroform and methanol, and activated with supercritical CO_2. Soxhlet purification was not performed in this series of synthesis, as it only provides a minor sharpness for 100 peak in PXRD and does not affect the comparison. The amount of the chemicals used for the colloid reactions are summarized in **Supplementary Table 1**. The reaction yield of the reactions was summarized in **Supplementary Fig. 19**.

TPB-HTP reaction. Following the procedure of TPB-MeOTP-NP, TPB-HTP was synthesized under [TAPB] of 2.81 mM and 1.97 mM, with the ratio of amine groups in TAPB and aldehyde groups in HTP and $Sc(OTf)_3$ amount fixed at 1:1 and 0.08 equivalent, respectively. Higher [TAPB] condition cannot be performed due to the limited solubility of HTP in acetonitrile. Different from TPB-MeOTP, TPB-MeTP and TPB-TP, the TPB-HTP product was precipitated out from the solution and no colloid solution was obtained. The obtained TPB-HTP has a very low crystallinity, suggested by the nearly invisible 100 diffraction peak (**Supplementary Fig. 15**).

Supplementary Table 1. The chemical amount for the reactions with varying [TAPB]

Reactions	[TAPB]	TAPB	MeOTP	Dichloromethane/acetonitrile	$Sc(OTf)_3$/acetonitrile
TPB-MeOTP	5.02 mM	20.8 mg	17.2 mg	0.35 mL/9.45 mL	7 mg/2 mL
	3.69 mM	20.8 mg	17.2 mg	0.5 mL/13.5 mL	7 mg/2 mL
	2.56 mM	20.8 mg	17.2 mg	0.75 mL/20.3 mL	7 mg/2 mL
	1.97 mM	20.8 mg	17.2 mg	1 mL/27 mL	7 mg/2 mL
	1.02 mM	20.8 mg	17.2 mg	2 mL/54 mL	7 mg/2 mL
	0.52 mM	20.8 mg	17.2 mg	4 mL/108 mL	7 mg/2 mL
TPB-MeTP	3.69 mM	20.8 mg	14.4 mg	0.5 mL/13.5 mL	7 mg/2 mL
	1.97 mM	20.8 mg	14.4 mg	1 mL/27 mL	7 mg/2 mL
	1.02 mM	20.8 mg	14.4 mg	2 mL/54 mL	7 mg/2 mL
	0.52 mM	20.8 mg	14.4 mg	4 mL/108 mL	7 mg/2 mL
TPB-TP	3.69 mM	20.8 mg	11.9 mg	0.5 mL/13.5 mL	7 mg/2 mL
	1.97 mM	20.8 mg	11.9 mg	1 mL/27 mL	7 mg/2 mL
	1.02 mM	20.8 mg	11.9 mg	2 mL/54 mL	7 mg/2 mL
	0.52 mM	20.8 mg	11.9 mg	4 mL/108 mL	7 mg/2 mL
TPB-HTP	2.81 mM	20.8 mg	14.7 mg	0.5 mL/18.5 mL	7 mg/ 2 mL
	1.97 mM	20.8 mg	14.7 mg	1 mL/ 27 mL	7 mg/ 2 mL

Reactions for time-dependent PXRD

The molar concentration of the linkers for time-dependent PXRD characterizations followed with the TPB-MeOTP-NP synthesis. Following the procedure of TPB-MeOTP-NP, TAPB (59.2 μmol), aldehyde linkers (88.6 μmol), and $Sc(OTf)_3$ (14.2 μmol), were used for the synthesis, and the amount of each chemical was summarized in the **Supplementary Table 2**. The reaction was stirred at room temperature for the specific time. After that, the colloid was precipitated out by 1 M NaCl aqueous solution. The precipitates were collected by filtration, washed with acetone, dimethylformamide, chloroform and methanol, and activated with supercritical CO_2. Soxhlet purification was not performed in this series of synthesis.

Supplementary Table 2. The chemical amount of the reactions for time-dependent PXRD

Reactions	TAPB	Aldehyde linker	Dichloromethane/acetonitrile	$Sc(OTf)_3$/acetonitrile
TPB-MeOTP	20.8 mg	17.2 mg	1 mL/27 mL	7 mg/2 mL
TPB-MeTP	20.8 mg	14.4 mg	1 mL/27 mL	7 mg/2 mL
TPB-TP	20.8 mg	11.9 mg	1 mL/27 mL	7 mg/2 mL

In-situ ^{1}H-NMR characterizations

The molar concentration of the linkers for *in-situ* ^{1}H-NMR characterizations followed with the TPB-MeOTP-NP synthesis. $CDCl_3$ was used as the co-solvent instead of CD_2Cl_2, due to its lower cost and very similar property with CD_2Cl_2. Here, the *in-situ* ^{1}H-NMR characterizations of TPB-MeOTP-NP was taken as an example to describe the procedure. TAPB (1.46 mg) and MeOTP (1.20 mg) was separately dissolved in two vials, containing a mixture of 35 μL $CDCl_3$ and 945 μL CD_3CN. $Sc(OTf)_3$ stock solution was prepared by dissolving 2.1 mg $Sc(OTf)_3$ in 560 μL CD_3CN. To start the colloid reaction, 490 μL of TAPB stock solution, 490 μL MeOTP stock solution and 70 μL $Sc(OTf)_3$ stock solution were mixed in a vial, defined as t=0 for the experiment. The resulting solution was transferred to a NMR tube and loaded in the NMR magnet. 54 continuous tests were carried out for TPB-MeOTP colloid reaction and the average time for each test was 54.8 s. For TPB-TP and TPB-MeTP colloid reactions, as their k_p is slower than that of TPB-MeOTP, 73 continuous tests were performed, and the average time for each test was 315.03 s.

Supplementary Table 3. The stock solutions for *in-situ* ^{1}H-NMR characterizations

Reactions	TAPB stock solution	MeOTP stock solution	$Sc(OTf)_3$ stock solution
TPB-MeOTP	1.46 mg/35 μL $CDCl_3$+945 μL CD_3CN	1.20 mg/35 μL $CDCl_3$+945 μL CD_3CN	2.1 mg /560 μL CD_3CN
TPB-MeTP	1.46 mg/35 μL $CDCl_3$+945 μL CD_3CN	1.00 mg/35 μL $CDCl_3$+945 μL CD_3CN	2.1 mg /560 μL CD_3CN
TPB-TP	1.46 mg/35 μL $CDCl_3$+945 μL CD_3CN	0.83 mg/35 μL $CDCl_3$+945 μL CD_3CN	2.1 mg /560 μL CD_3CN

Solvothermally synthesized TPB-MeOTP

A Biotage 2 mL high precision glass vial was charged with TAPB (35.1 mg, 100 μmol), MeOTP (29.1 mg, 150 μmol), acetic acid (6 M, 125 μL), dichlorobenzene (625 μL) and butanol (625 μL), and degassed by purging Ar for 10 min. Then the vial was heated at 120 °C for three days. After cooling to room temperature, the product was collected with a filter paper, washed with acetone, dimethylformamide, chloroform and methanol, and purified with methanol in a Soxhlet extractor for 12 h. After activating with supercritical CO_2, 52.2 mg of TPB-MeOTP was obtained, offering a reaction yield of 88.8%.

3. Supplementary Methods

Ellipsometry modeling

The complex refractive index of the TPB-MeOTP-NP film, Fig. 5f ($\sqrt{\epsilon_b\,(E)} = n(E) + ik(E)$), was obtained from ellipsometry measurements at room temperature (Sopra PS-1000 SAM). We employed a genetic algorithm to fit the experimental data assuming a Forouhi-Bloomer model for three oscillators[5-6]:

$$n(E) = n_\infty + \sum_{j=1}^{3} \frac{B_j(E - E_j) + C_j}{(E - E_j)^2 + \Gamma_j^2}; \quad k(\omega) = \begin{cases} \sum_{j=1}^{3} \frac{f_i(E - E_g)^2}{(E - E_j)^2 + \Gamma_j^2}, & E > E_g \\ 0, & E \leq E_g \end{cases} \quad (1)$$

where

$$B_j = \frac{f_j}{\Gamma_j}\left(\Gamma_j^2 - (E_j - E_g)^2\right) \text{ and } C_j = 2 f_j \Gamma_j (E_j - E_g) \quad (2)$$

here E is the photon energy, E_g is the bandgap energy, and E_j, f_j, and Γ_j are the position, strength, and width of one oscillator. In order to make the method more accurate and reliable, two films with different thicknesses at three different incident angle (60°, 65° and 70°) measurements were fitted simultaneously.

A dielectric function ϵ_p models the porosity of the film assuming a Bruggeman effective medium approximation:

$$\frac{1 - \epsilon_\mathrm{p}(E)}{1 - 2\epsilon_\mathrm{p}(E)} p + \frac{\epsilon(E) - \epsilon_\mathrm{p}(E)}{\epsilon(E) + 2\epsilon_\mathrm{p}(E)}(1 - p) = 0 \quad (3)$$

where p is the porosity, i.e. the air to COF ratio, of the layer. A layer of arbitrary thickness (d_r, 0 nm - 40 nm and a porosity of p_r) and dielectric function ϵ_r models the surface roughness of the film assuming also Bruggeman effective medium approximation. To reduce the impact of randomness on the initial fitting parameters, some of the parameters were appropriately bounded according to the experimental values of the layer thickness (from SEM analysis) and the bandgap (from Tauc plot). Dielectric function parameters of the best fitting was summarized in **Supplementary Table 4**. Once the refractive index is obtained, we used it to calculate the electric field intensity distribution and absorption maps following the transfer matrix method (TMM) and the following equation[7] for a sample immersed in water:

$$A_j = 2\pi \int \frac{I(x_j, \lambda)}{I_0(\lambda)} \frac{2 n_j k_j}{\lambda} dx_j \quad (4)$$

where the j refers to each layer in the sample and λ is the wavelength. The integral extends over the volume occupied by each medium, which can be reduced to one dimension (x_j) due to the symmetry of the system.

Supplementary Table 4. Dielectric function parameters of the best fitting

parameter	value
p_r	0.51
p_p	0.36
d_r (nm)	10
d_1 (nm)	320
d_2 (nm)	846
n_∞	1.344
E_g (eV)	1.882
f_1	0.089
Γ_1 (eV)	0.222
E_1 (eV)	2.227
f_2	0.017
Γ_2 (eV)	0.238
E_2 (eV)	2.806
f_3	0.015
Γ_3 (eV)	0.747
E_3 (eV)	3.930

Solubility estimation

The solubility of TP, MeOTP, MeTP and HTP in acetonitrile at room temperature was estimated by adding the minimal volume of acetonitrile in a known amount of linker and making the linker fully dissolve. Sonication and stirring were performed for assisting the dissolution. No heating treatment was used, as the colloid reaction was performed at room temperature.

PFG NMR characterization

The pulsed field gradient NMR technique (PFG NMR) was applied to determine the diffusion coefficient (D) of TP and MeOTP at different concentrations between 12 mM and 0.75 mM (TP) or 0.19 mM (MeOTP) in acetonitrile-d_3. To dissolve MeOTP at high concentrations, heating was performed for the solution. While the high concentration MeOTP solution remained clear during the PFG NMR test at 300 K, MeOTP precipitated out after 1 day.

The D of molecules in solution can be described by the Stokes-Einstein equation (1), containing the Boltzmann constant (k_B), the temperature (T), the viscosity (η) and the hydrodynamic radius of the diffusing species. The diffusion is inversely proportional to the hydrodynamic radius if the temperature and solution viscosity remain constant. As a consequence, changes in D at different concentrations can be used to study the self-assembly behavior of the solute, as the self-assembly of multiple molecules increases the apparent hydrodynamic radius[8-10] of the diffusing species.

$$D = \frac{k_B T}{6\pi\eta r_H} \quad (1)$$

Tetramethylsilane was added as an internal standard for the solvent viscosity, which remained constant at the measured solute concentrations. The measurements were performed in 5 mm tubes on a Bruker Avance III 400 MHz spectrometer at 300 K with a stimulated-echo sequence[11] and default spoiler gradient (*diffSte* program, Bruker TopSpin). A gradient pulse with a length δ = 1 ms (*opt* shape) and a diffusion time Δ = 50 ms were applied. The gradient was varied linearly in 16 steps between 2.33 and 46.55 Gs/cm. Data processing was performed with the Bruker TopSpin 3.5 *Dynamics* module using automated peak picking, integration and fitting of the signal area *S* vs. gradient *B* according to the Stejskal-Tanner equation (Eq. 2). Experiments were performed multiple times with an appropriate number of scans for the individual sample concentration (standard deviation is shown as error bars).

$$\frac{S}{S_0} = e^{-BD} \quad (2)$$

The resulting *D* of TP and MeOTP at different concentrations is shown in **Supplementary Figure 30**. The diffusion coefficient of Tp and MeOTP in deuterated acetonitrile increases with decreasing concentration, indicating that both building blocks form molecular aggregates in solution. However, TP molecules interact less strongly compared to MeOTP, visible from the early increase of the diffusion coefficient with decreasing concentration, which correlates well with the fast crystallization rate of TPB-MeOTP as well as the preferred particle growth of nanoplates along the interlayer direction. We can infer that the crystallite formation in this system is directed by the template effect of the self-assembly in solution.

4. Supplementary Figures

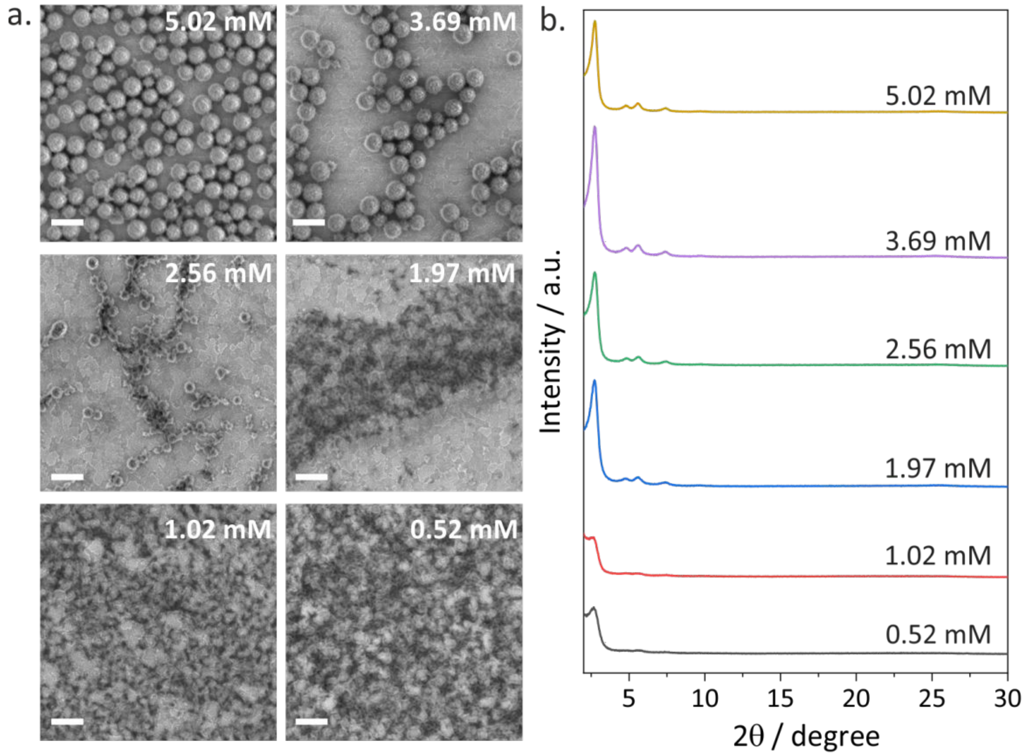

Supplementary Figure 1. SEM images (a) and PXRD patterns (Cu-$K_{\alpha 1}$) (b) of colloidal TPB-MeOTP COF synthesized with various [TAPB]. The scale bar in the SEM images is 500 nm. The images were taken at 1.5 kV with an InLens detector. SEM images indicate that with the [TAPB] of 5.02 mM and 3.69 mM nanospheres were obtained, and the [TAPB] of 2.56 mM resulted in a mixture of nanospheres and nanoplates. Although SEM image could not clearly show the nanoparticle shape with the [TAPB] of 1.97 mM, 1.02 mM and 0.52 mM, the nanoparticle morphology change can be observed. Further characterizations regarding the shape of TPB-MeOTP-NP were performed by AFM (Fig. 2d in the main text) and TEM (Fig. 3a in the main text and Supplementary Fig. 14).

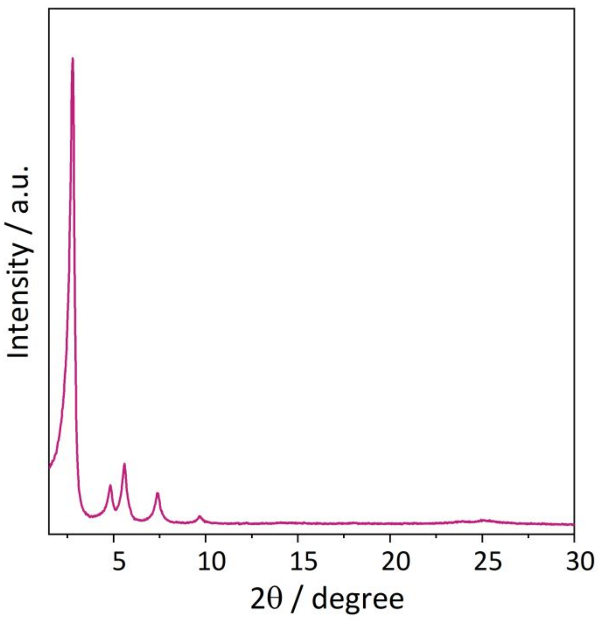

Supplementary Figure 2. PXRD patterns (Cu-K$_{\alpha1}$) of TPB-MeOTP COF synthesized via the solvothermal route.

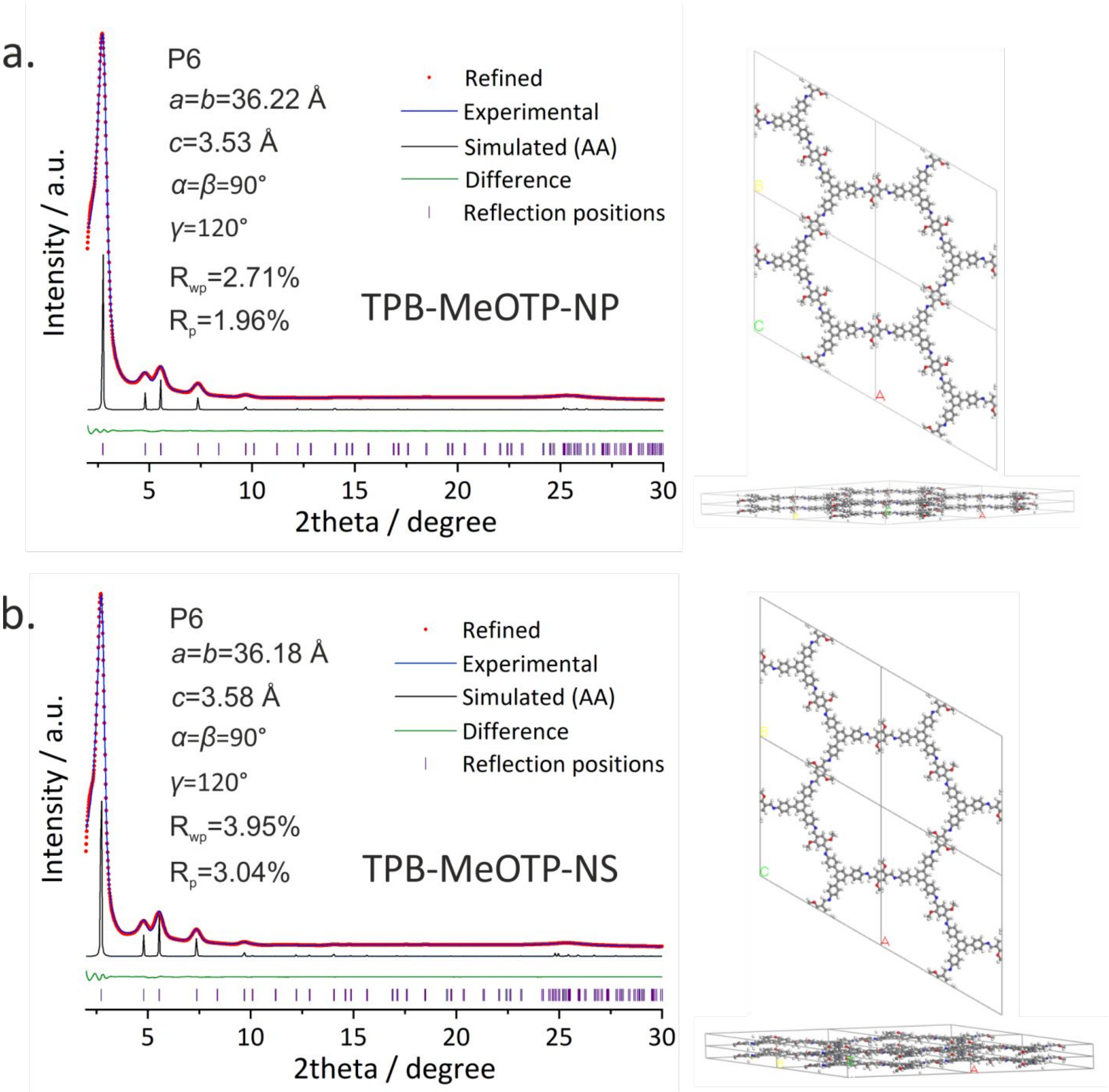

Supplementary Figure 3. PXRD profiles (Cu-$K_{\alpha 1}$) of TPB-MeOTP-NP (a) and TPB-MeOTP-NS (b). Experimental observed (blue), Pawley refined (red), and simulated PXRD with AA stacking mode (black). Simulated COF structures are also displayed on the right side.

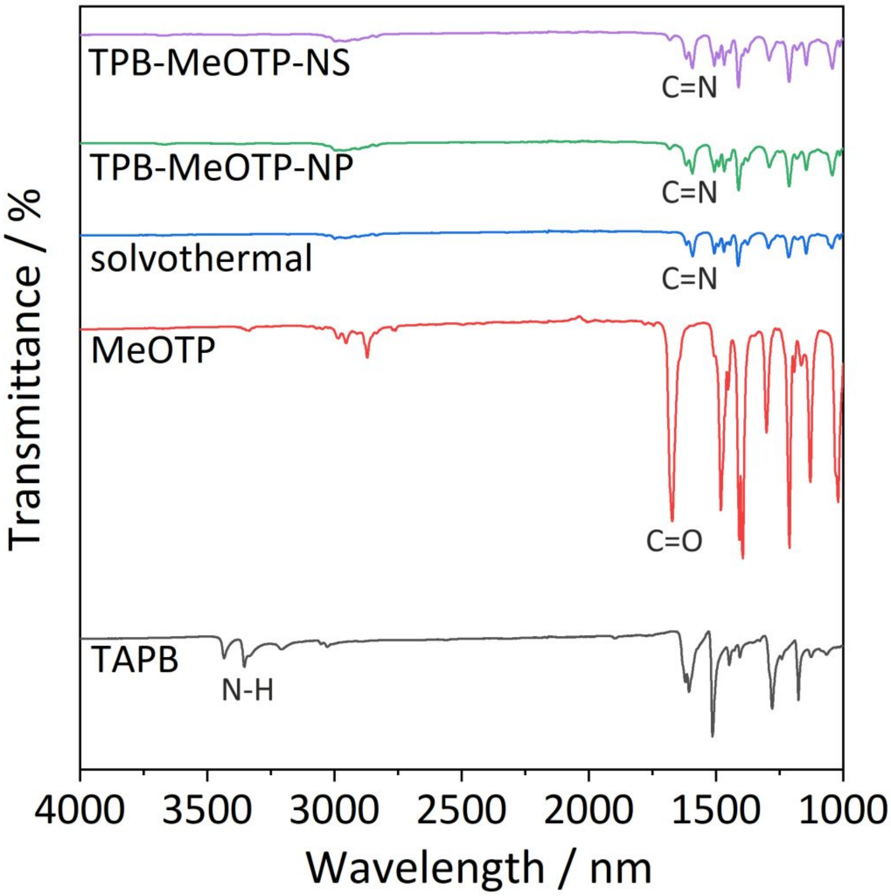

Supplementary Figure 4. FT-IR spectra of TPB-MeOTP-NP, TPB-MeOTP-NS and TPB-MeOTP synthesized by solvothermal approach.

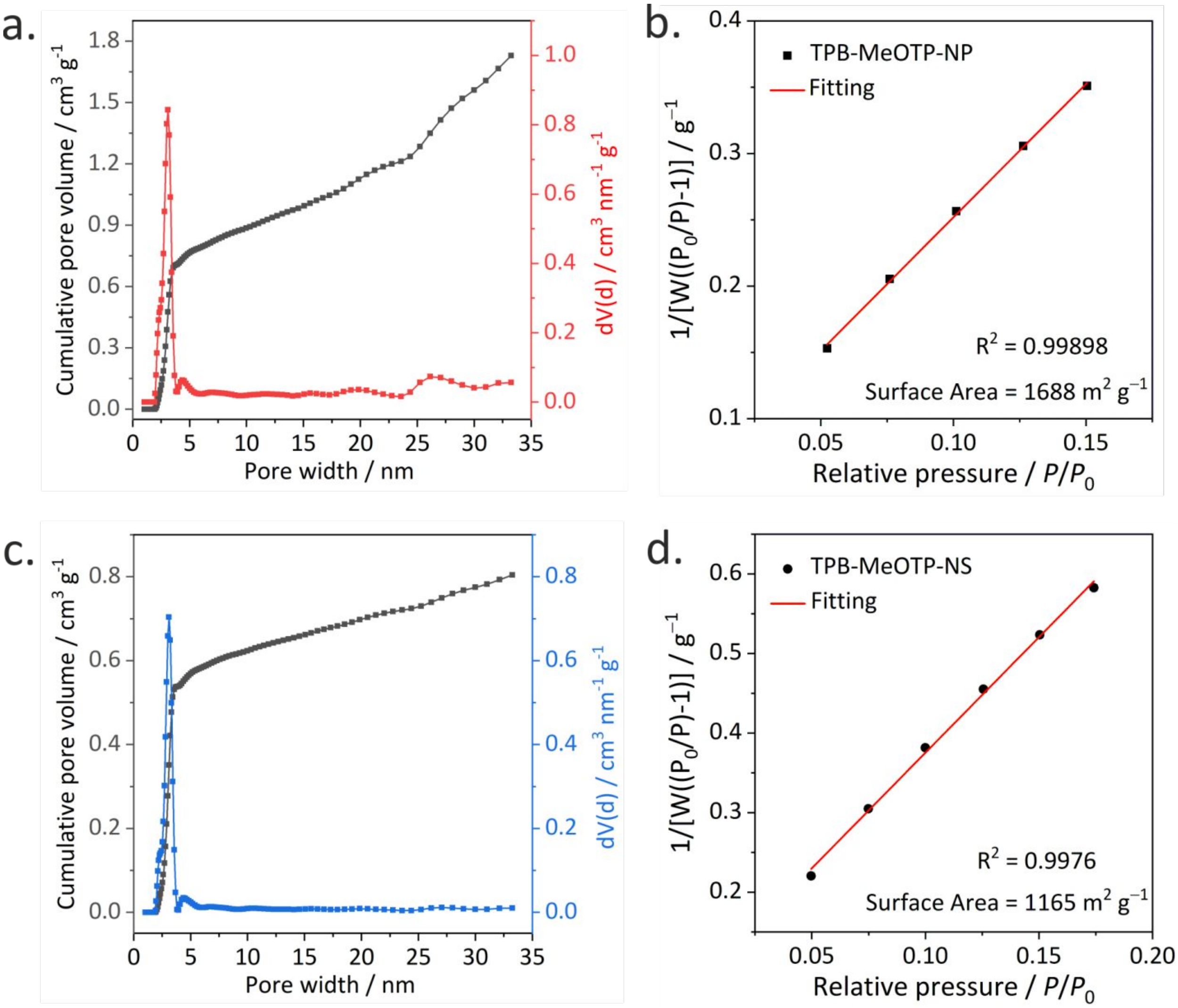

Supplementary Figure 5. Pore size distribution (a, c) and BET plot (b, d) of TPB-MeOTP-NP (a, b) and TPB-MeOTP-NS (c, d).

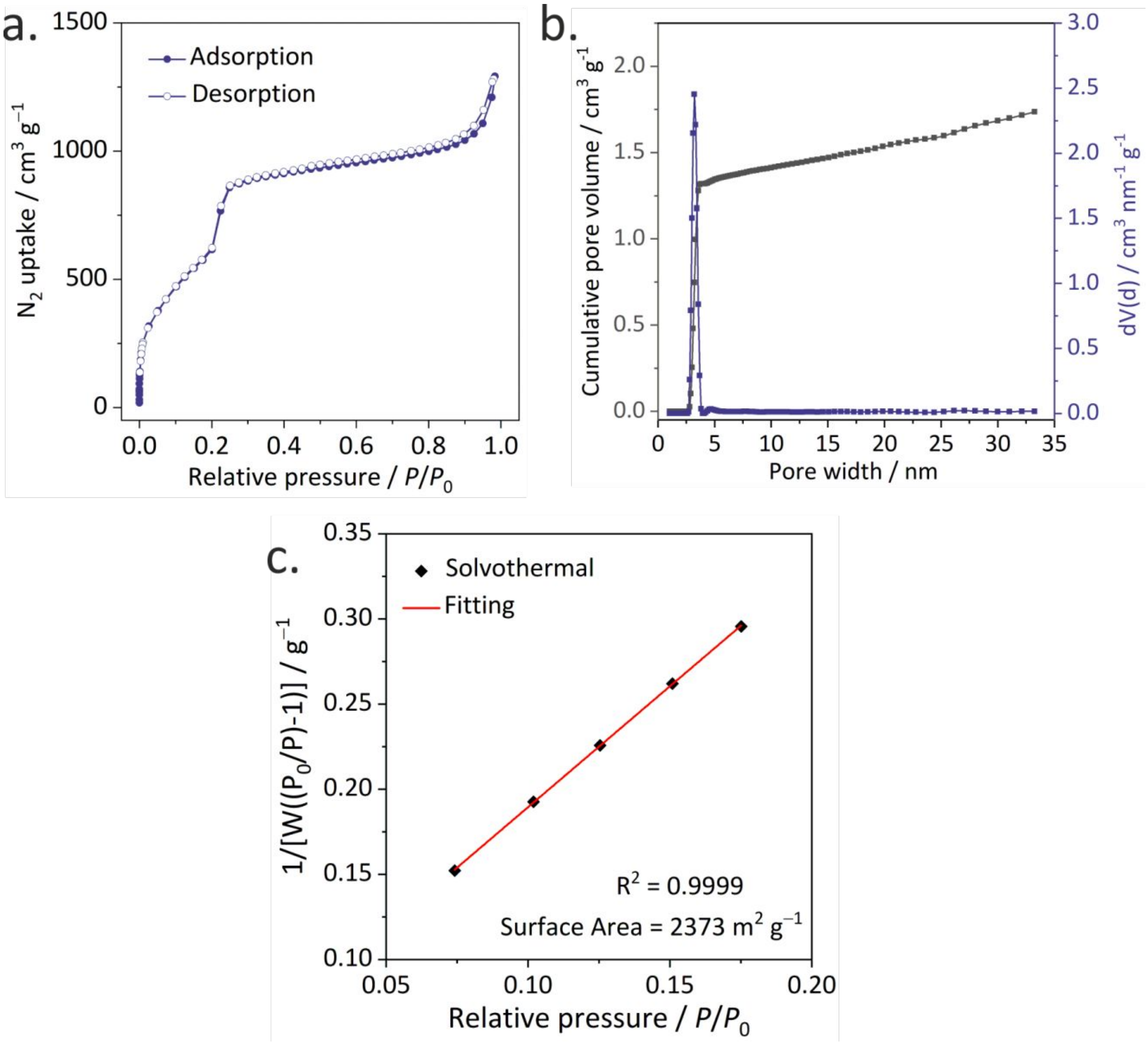

Supplementary Figure 6. Nitrogen adsorption at 77 K of solvothermally prepared TPB-MeOTP. N_2 adsorption (filled) and desorption (empty) isotherm profiles (a), pore size distribution (b) and BET plot (c).

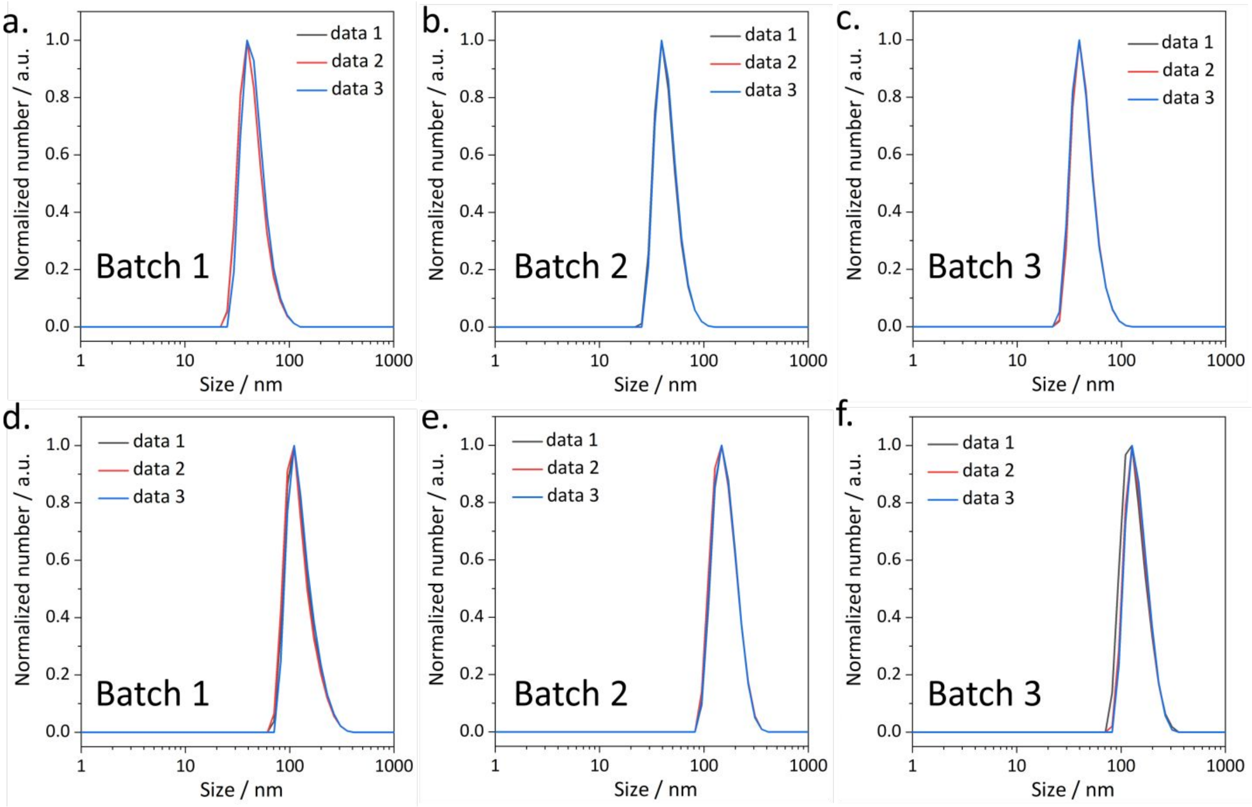

Supplementary Figure 7. DLS particle radius size distribution of TPB-MeOTP-NP (a-c) and TPB-MeOTP-NS (d-f). Three independent batches are shown and each batch was measured three times, validating the reproducibility of TPB-MeOTP-NP and TPB-MeOTP-NS synthesis.

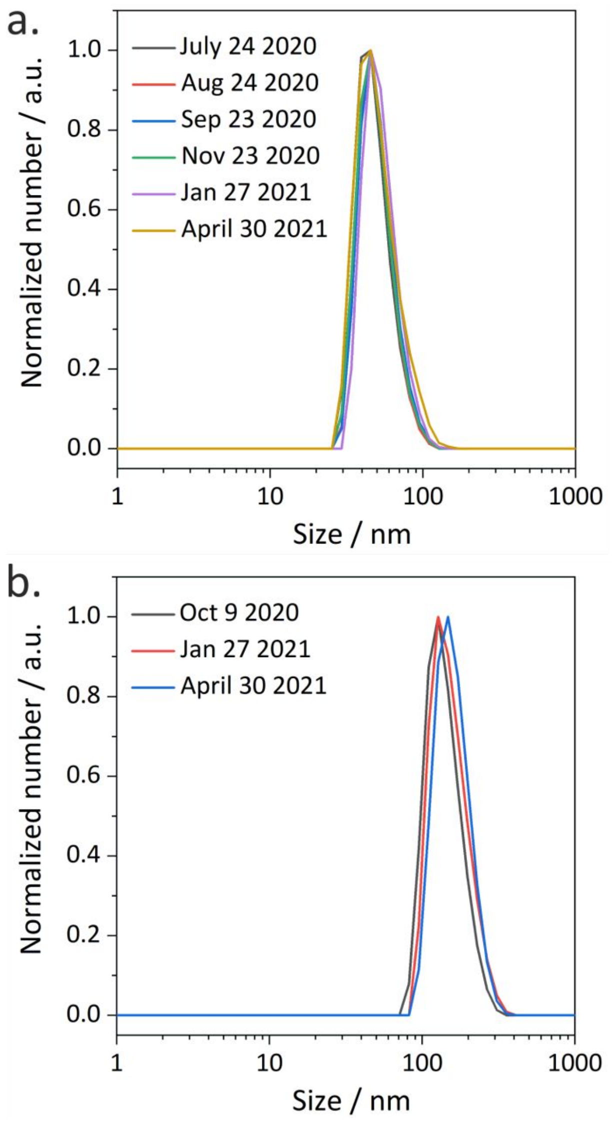

Supplementary Figure 8. Stability of TPB-MeOTP-NP (a) and TPB-MeOTP-NS (b). TPB-MeOTP-NP and TPB-MeOTP-NS with the concentration of 4.5 mg mL^{-1} were prepared on June 24, 2020 and Oct 9, 2020, respectively. DLS particle radius size distribution was measured on the day specified in the graph.

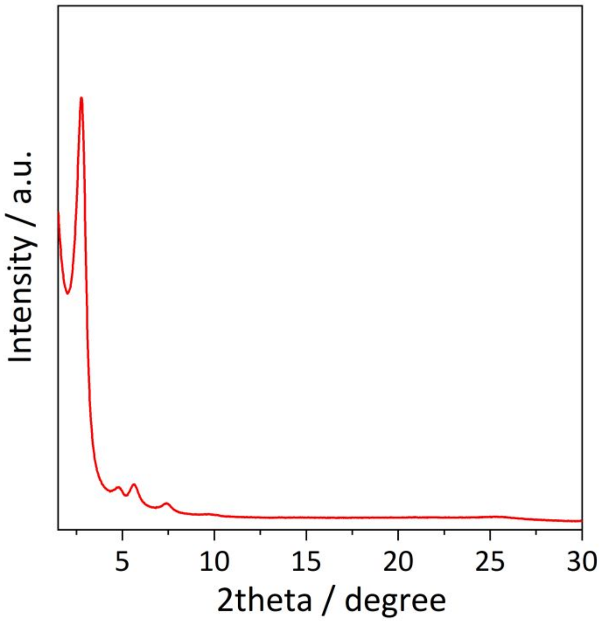

Supplementary Figure 9. PXRD pattern (Cu-$K_{\alpha 1}$) of TPB-MeOTP-NP with storing the colloid solution for 4 months. The colloids were precipitated with 1 M NaCl aqueous solution, washed with acetone, dimethylformamide, chloroform and methanol, and subjected to Soxhlet extraction with methanol for 12 h. After that, TPB-MeOTP-NP was activated with supercritical CO_2.

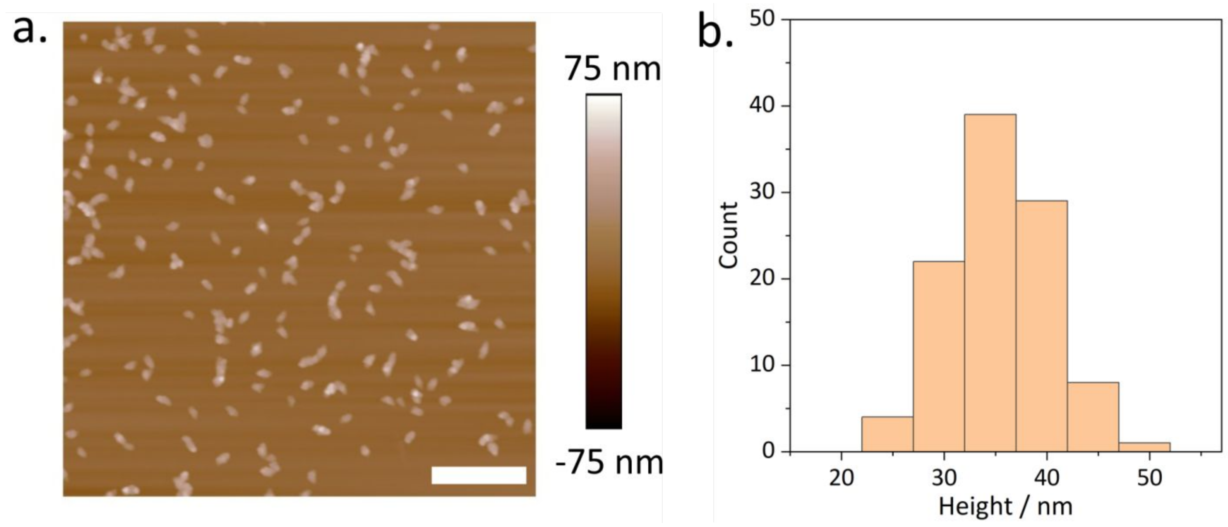

Supplementary Figure 10. AFM height image (a) of TPB-MeOTP-NP and height distribution histogram (b) of 103 particles. The scale bar in the AFM image is 1 μm.

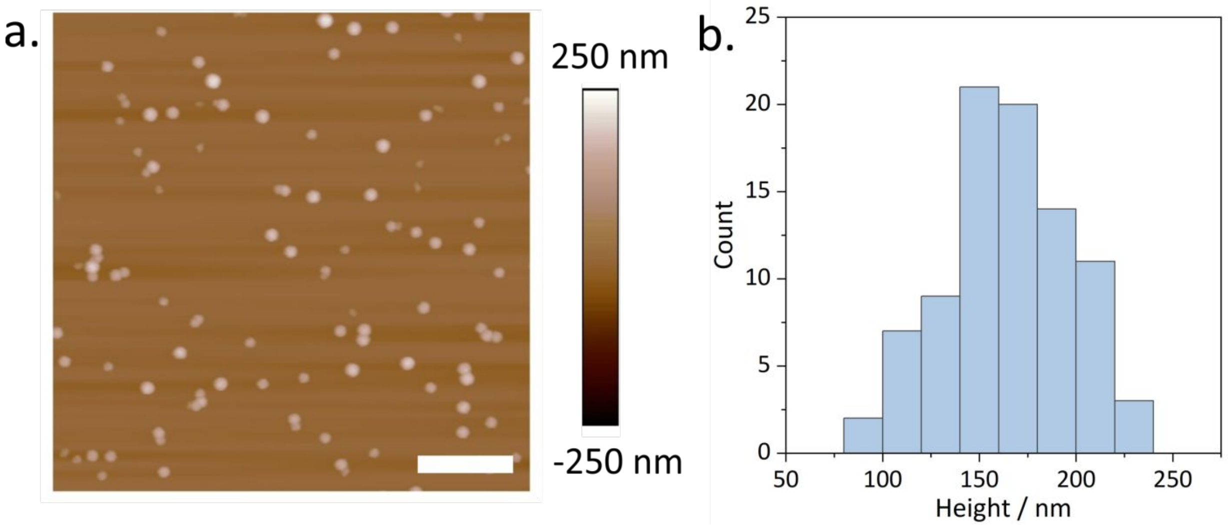

Supplementary Figure 11. AFM height image (a) of TPB-MeOTP-NS and height distribution analysis (b) of 87 particles. The scale bar in the AFM image is 2 μm.

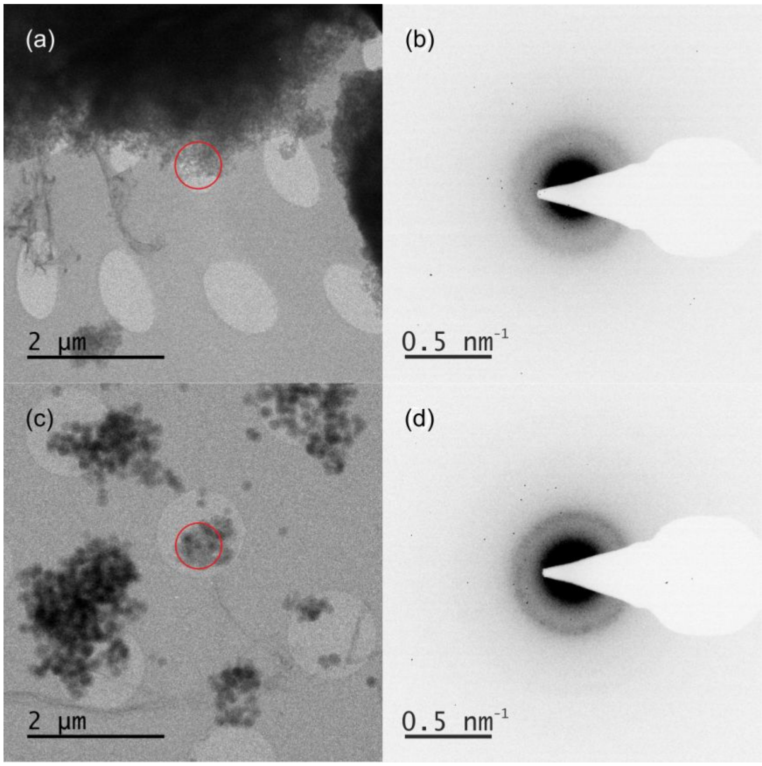

Supplementary Figure 12. (a) and (c) Bright-field TEM images of TPB-MeOTP-NP and TPB-MeOTP-NS, respectively. The red circles mark the positions of the selected-area aperture. (b) and (d) Low-dose SAED patterns obtained from the circular regions in (a) and (c), respectively. Total electron dose: 2 $e^{-}/Å^{2}$.

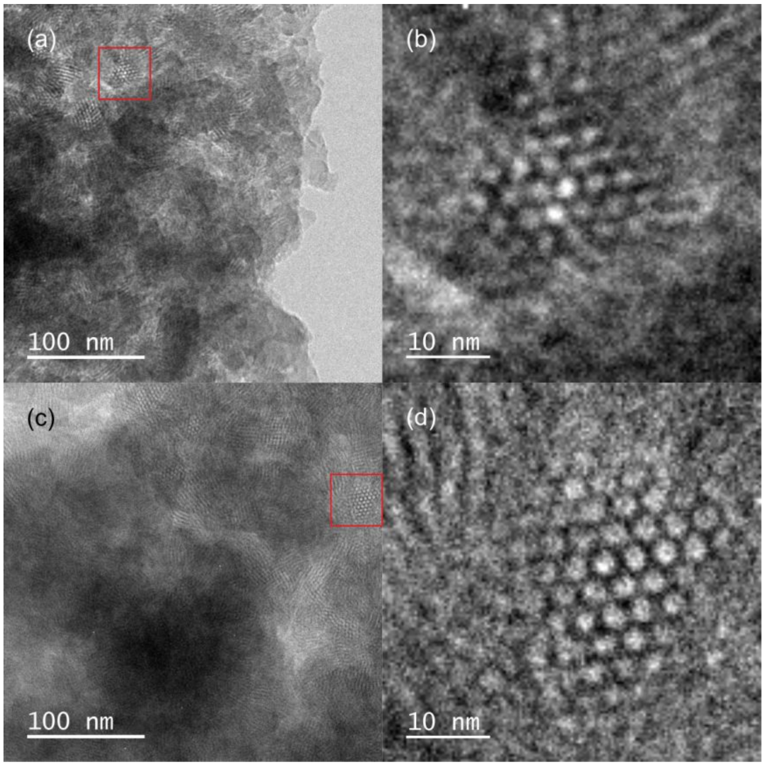

Supplementary Figure 13. (a) and (c) Low-dose HRTEM images of TPB-MeOTP-NP and TPB-MeOTP-NS, respectively. Total electron dose: 70 $e^{-}/Å^{2}$. (b) and (d) Magnified images from the boxed regions in (a) and (c), respectively.

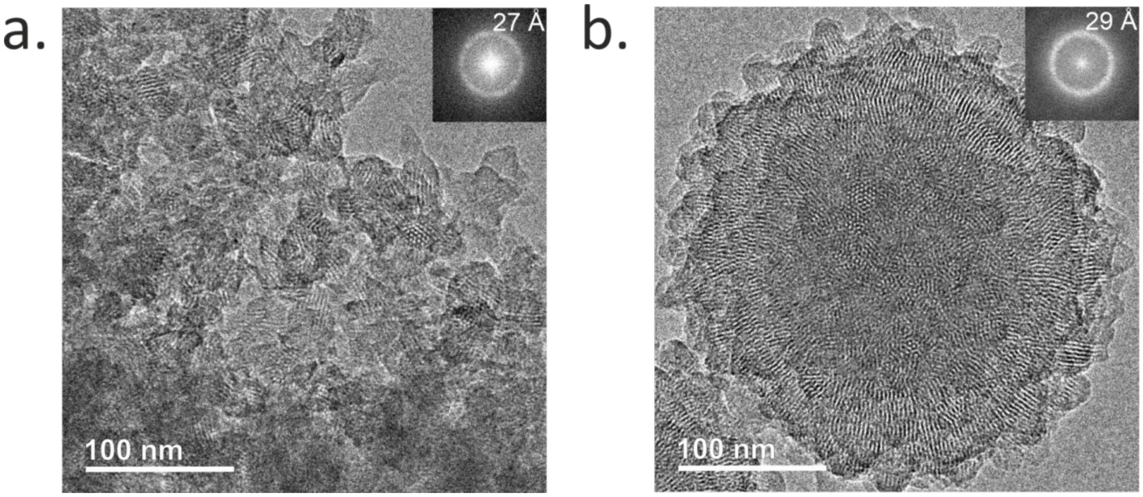

Supplementary Figure 14. TEM images of TPB-MeOTP-NP and TPB-MeOTP-NS. The images were obtained with a Philips CM30 ST (300kV, LaB6 cathode). The 100 reflection ring can be clearly observed in the FFT pattern (inset graph). The cross-check for the samples with two different machine systems in different labs confirms the reproducibility and reliability of the TEM results.

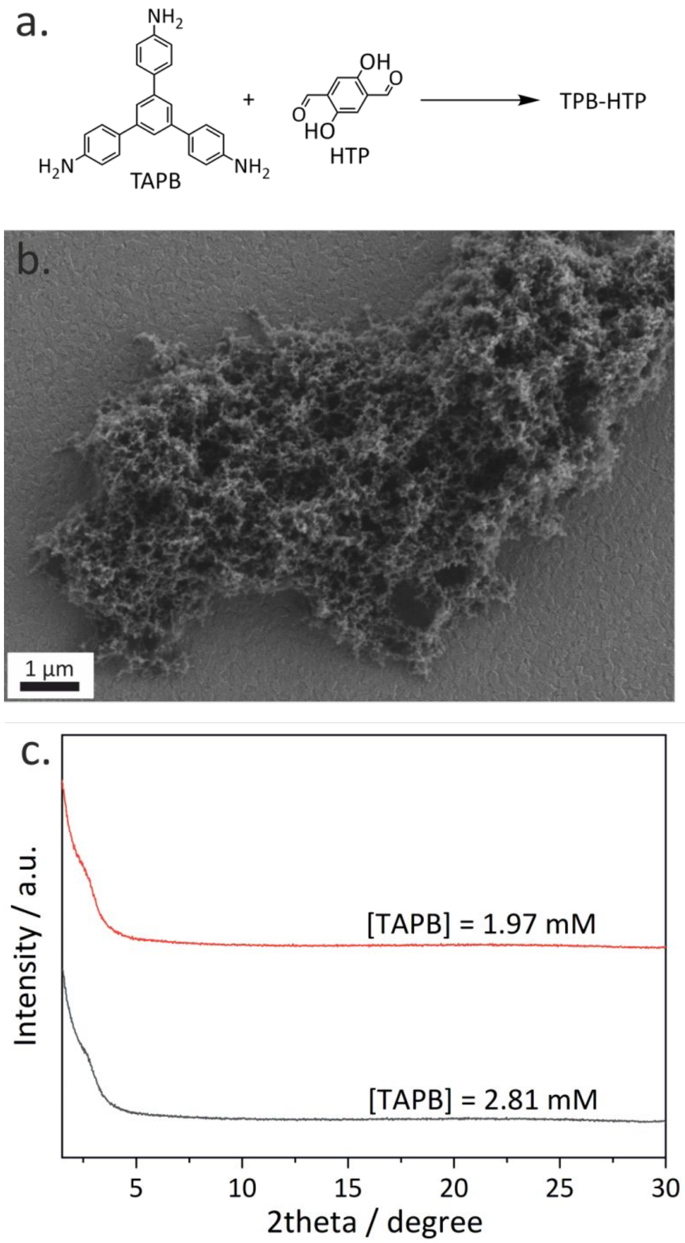

Supplementary Figure 15. a. Synthesis of TPB-HTP with DCM/acetonitrile as solvent and $Sc(OTf)_3$ as catalyst, details shown in Supplementary Synthesis Method. b. SEM image of TPB-HTP product. The SEM image was taken at 1.5 kV with a SE2 detector. c. PXRD patterns (Cu-$K_{\alpha 1}$). The results indicate TPB-HTP COF did not form colloids, likely related to the unsuccessful stabilization of TPB-HTP COF particles in acetonitrile.

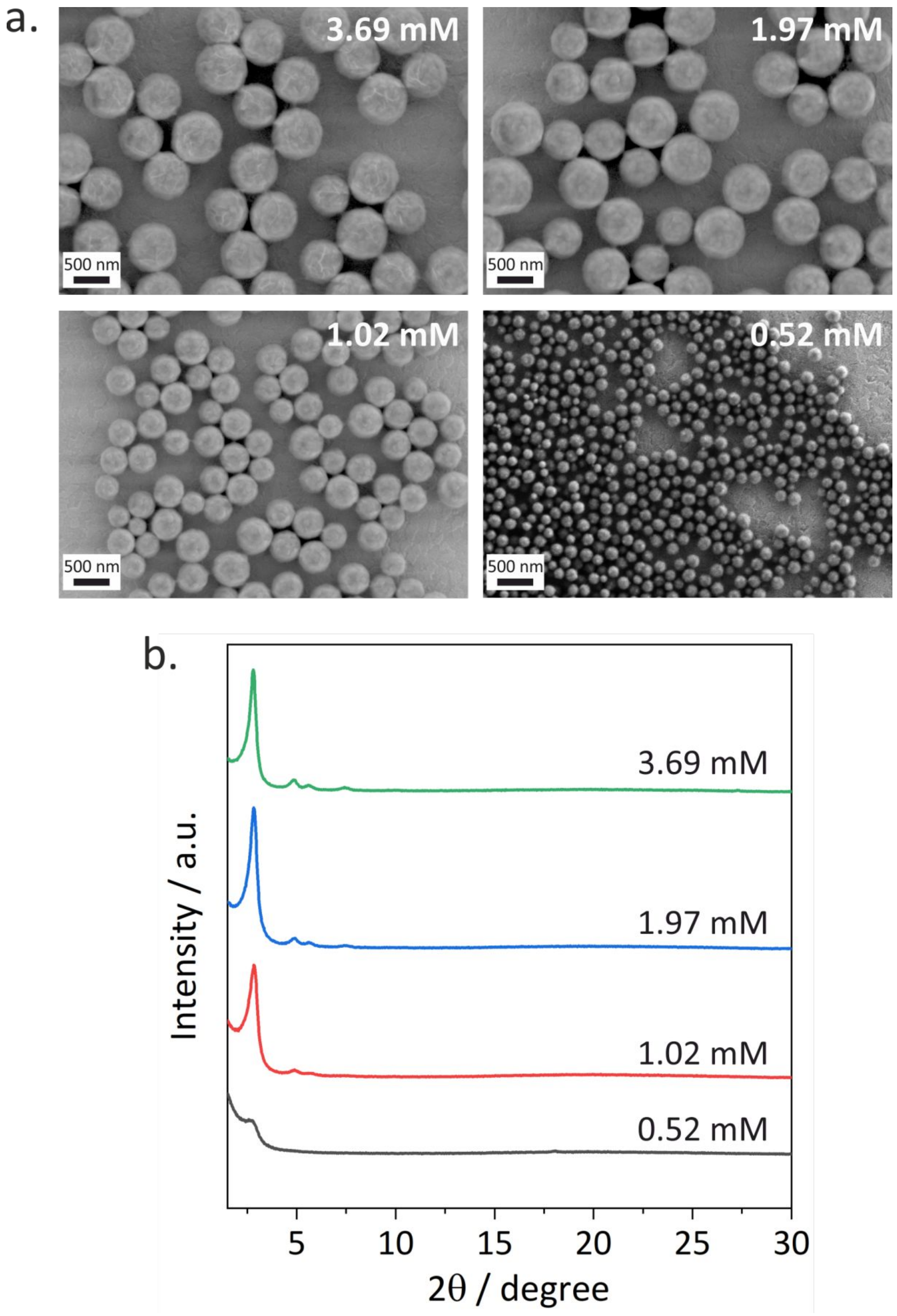

Supplementary Figure 16. SEM images (a) and PXRD patterns (b) of colloidal TPB-TP synthesized with various [TAPB]. The images were taken at 1.5 kV with a SE2 detector.

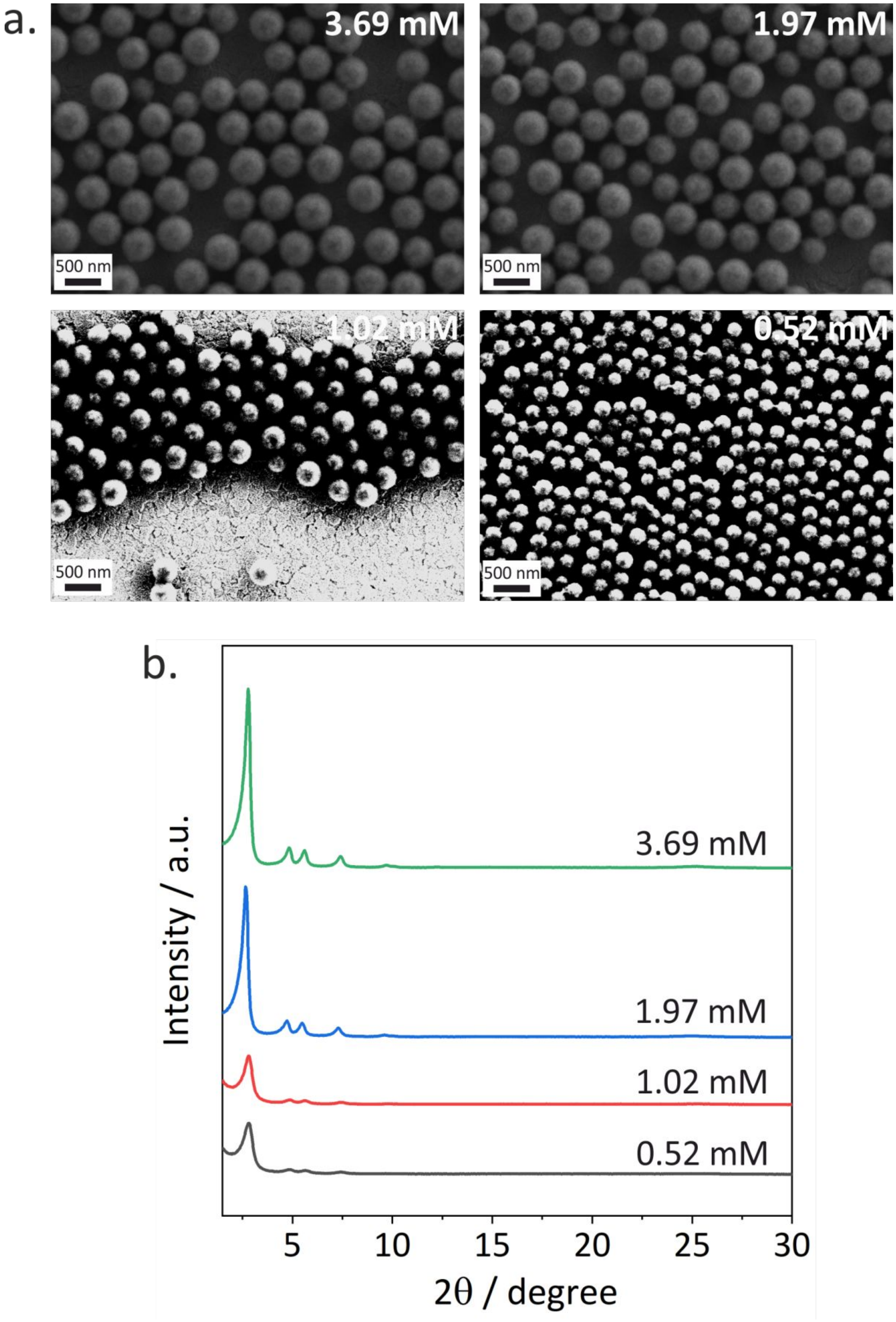

Supplementary Figure 17. SEM image (a) and PXRD patterns (b) of colloidal TPB-MeTP synthesized with various [TAPB]. The images were taken at 1.5 kV with a SE2 detector.

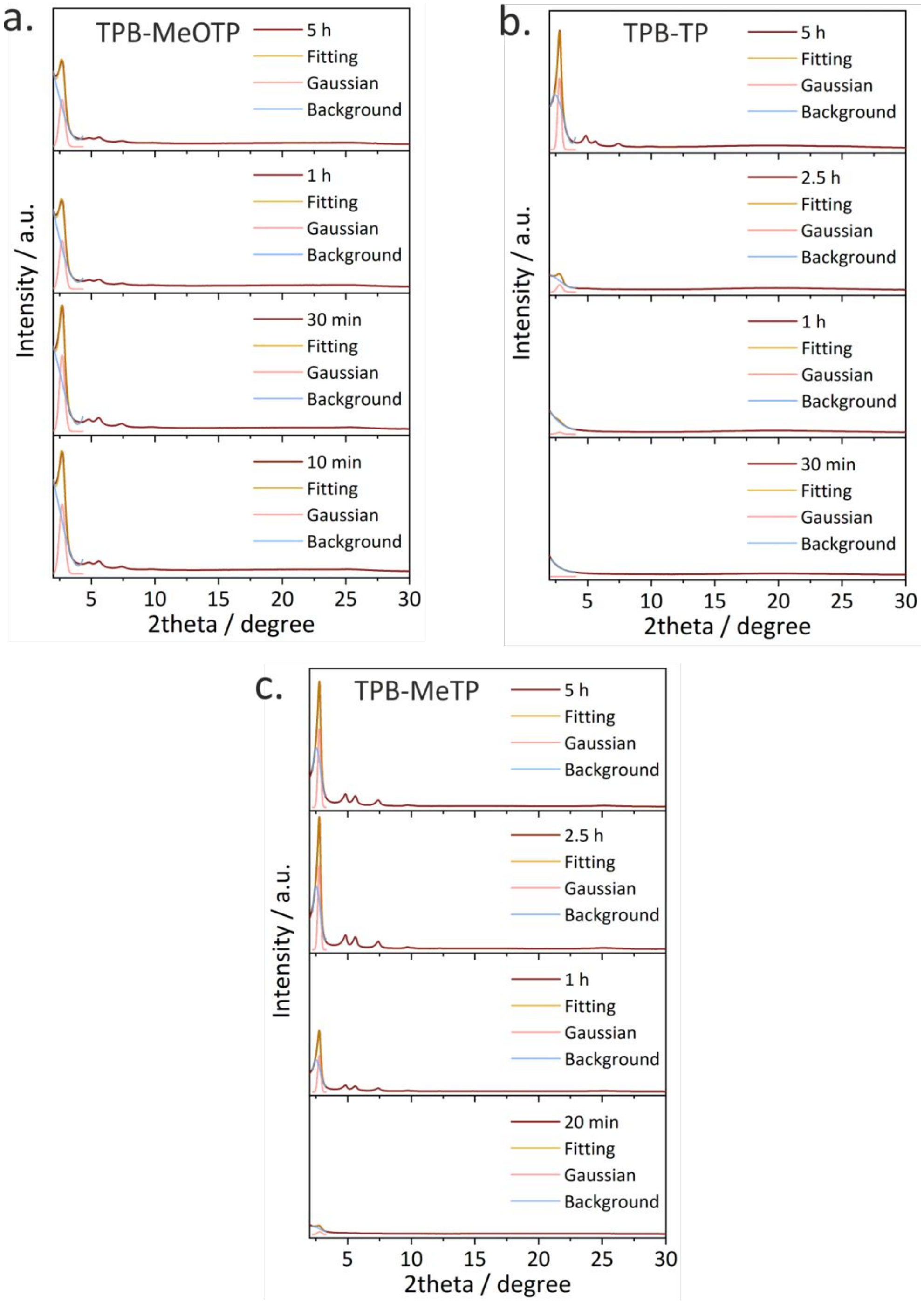

Supplementary Figure 18. Time dependent PXRD patterns of TPB-MeOTP, TPB-TP and TPB-MeTP. The FWHM values of 100 peaks were obtained by fitting the 100 peak with a Gaussian function and a cubic polynomial function for subtracting the background. The fitting result, Gaussian component and background component are displayed. FWHM value was calculated from the Gaussian component by FWHM=$2\sqrt{2\ln 2}\sigma$, where σ is the standard deviation. It is noticed that the 100 peak intensity of TPB-TP and TPB-MeTP shows a clear increasing trend with increasing reaction time. Instead, for TPB-MeOTP, no obvious intensity evolution trend is observed, and the minor intensity difference between samples is due to batch-to-batch variation.

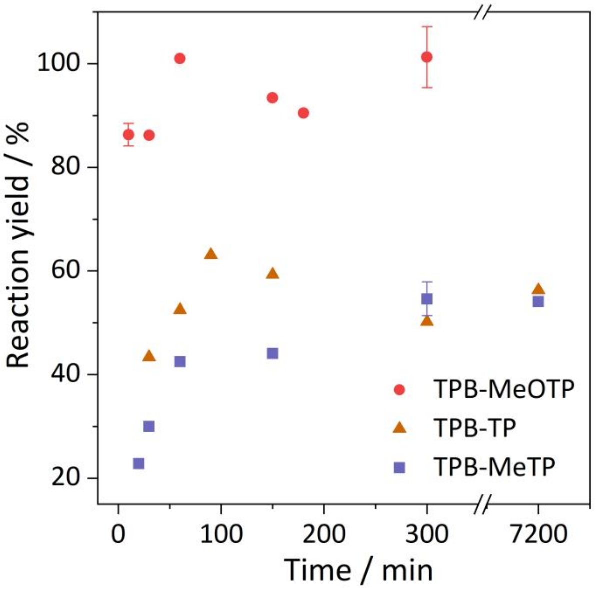

Supplementary Figure 19. Reaction yield of colloidal TPB-MeOTP, TPB-TP and TPB-MeTP with respect to time. The reaction yield statistics of TPB-MeOTP-10 min, TPB-MeOTP-300 min and TPB-MeTP-300 min is calculated from three, two and two independent batches, respectively. Synthesis details are shown in Supplementary Synthesis Method.

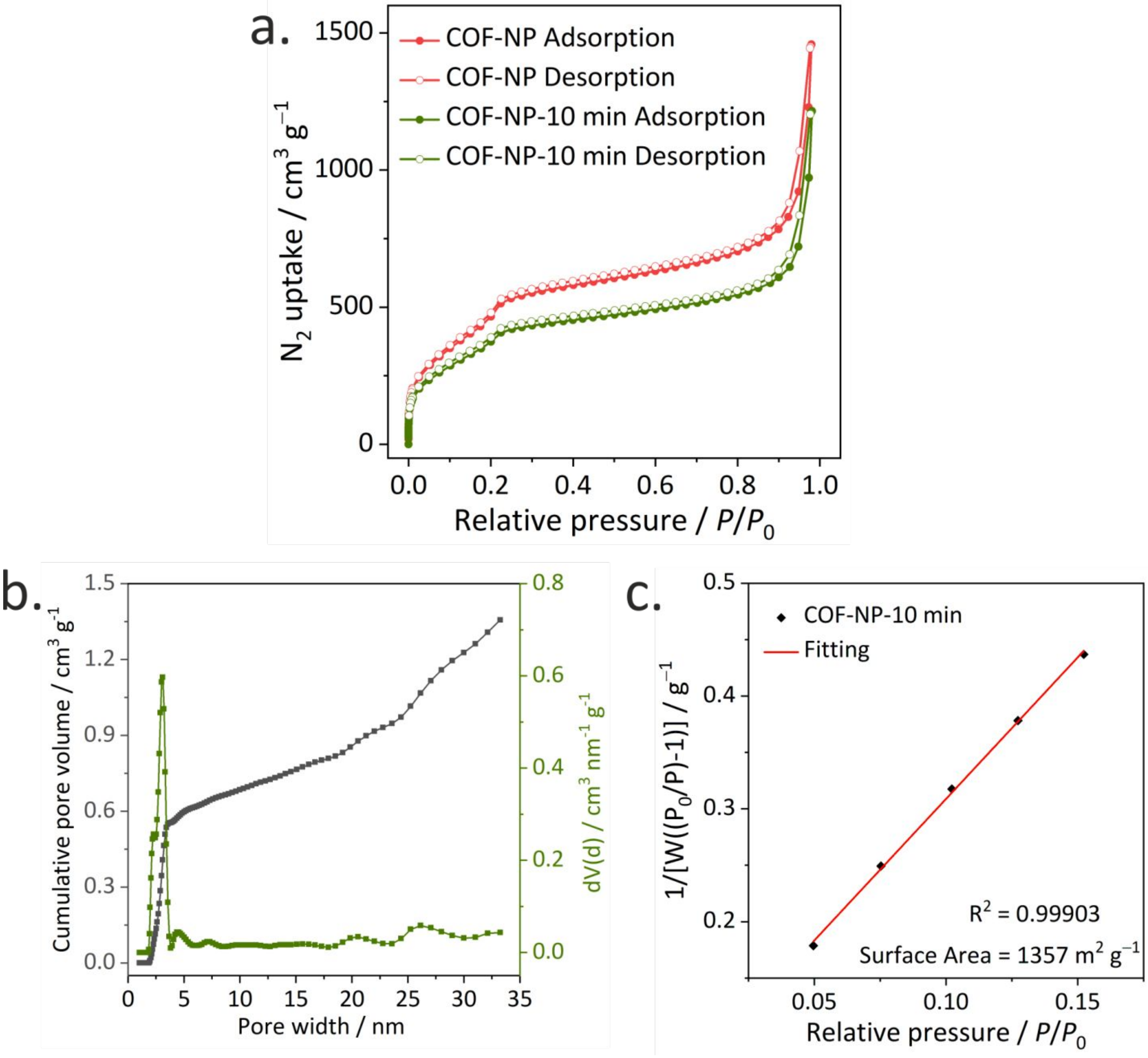

Supplementary Figure 20. Nitrogen adsorption characterization at 77 K for TPB-MeOTP-NP with 10 min reaction. a) N_2 adsorption (filled) and desorption (empty) isotherm profiles. The isotherm profiles of TPB-MeOTP-NP with 20 h reaction are plotted together for comparison. b) Pore size distribution. c) BET plot. COF-NP refers to TPB-MeOTP-NP.

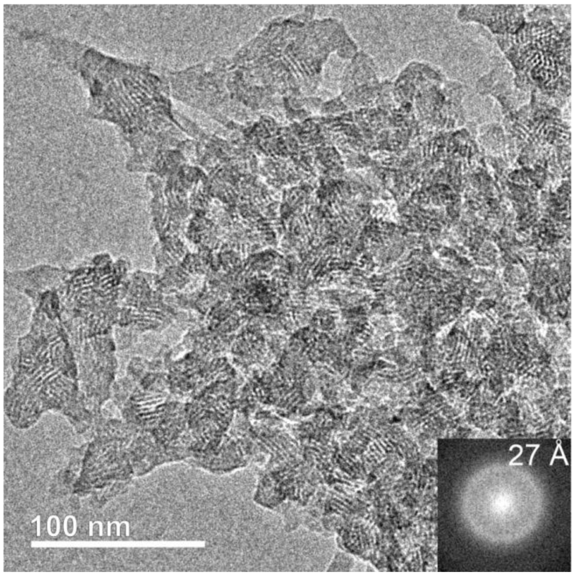

Supplementary Figure 21. TEM of TPB-MeOTP-NP with 10 min reaction time, obtained with a Philips CM30 ST (300kV, LaB_6 cathode). FFT image is shown in the inset graph.

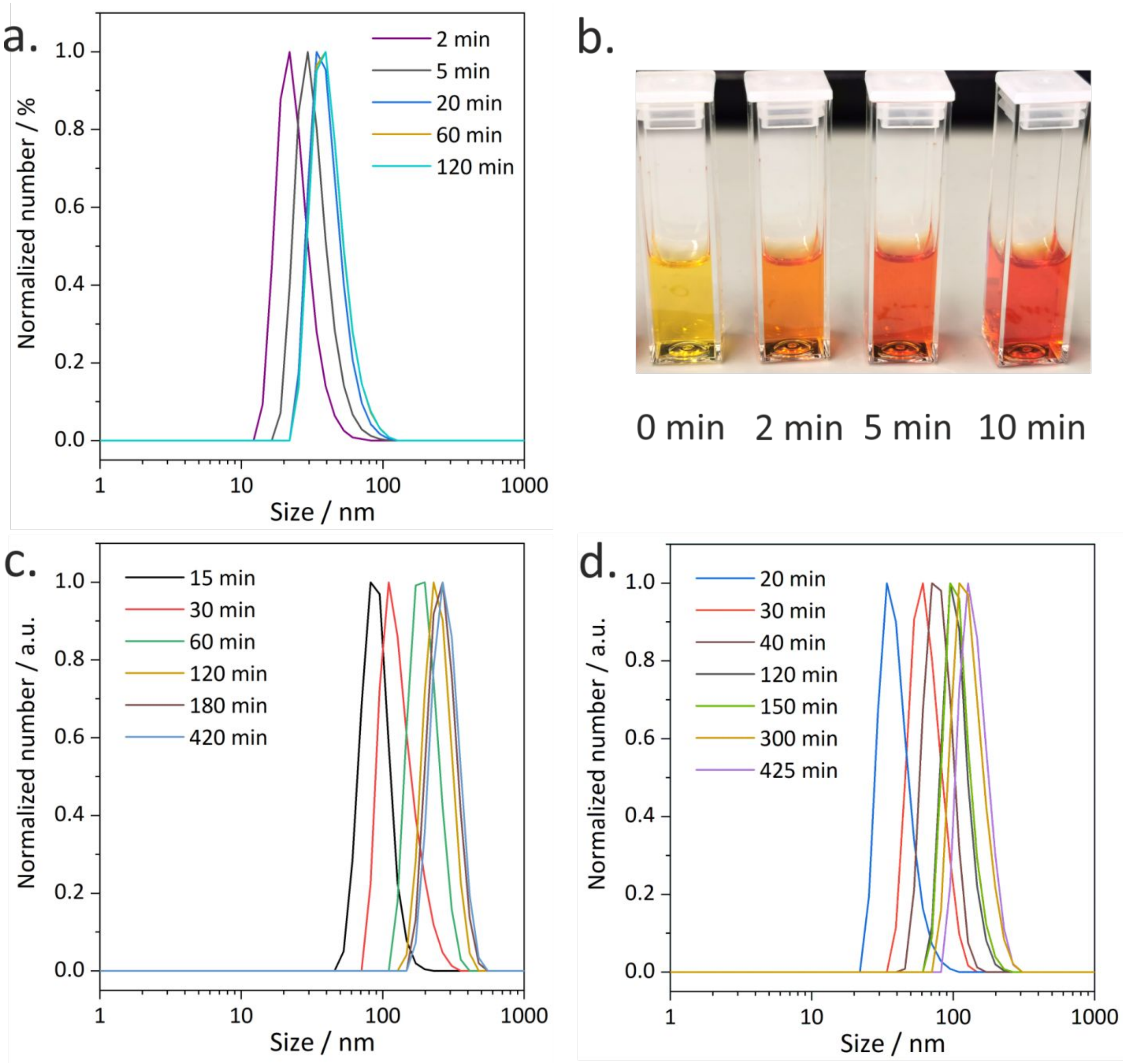

Supplementary Figure 22. DLS particle radius size distribution evolution of TPB-MeOTP (a), TPB-TP (c) and TPB-MeTP (d) with respect to time. (b) shows the image of diluted TPB-MeOTP colloids for DLS measurement at the specific time.

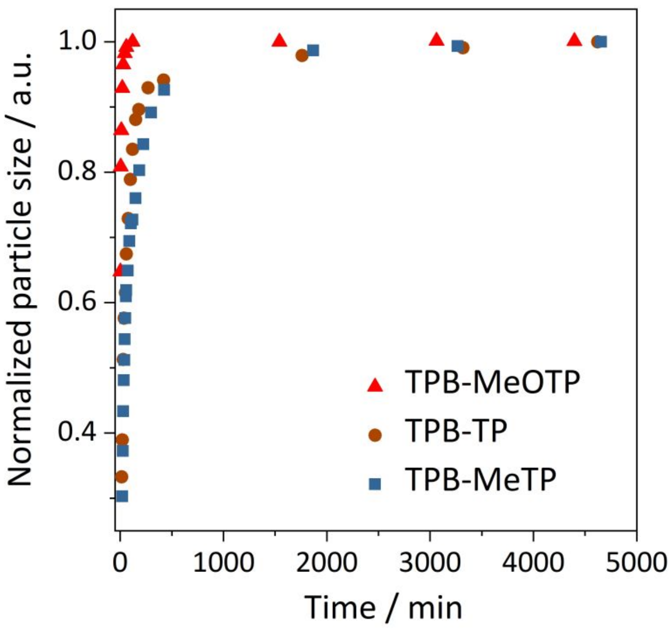

Supplementary Figure 23. Particle size (radius) variation of TPB-MeOTP, TPB-TP and TPB-MeTP as a function of time. The particle size is normalized with the size after 3 days.

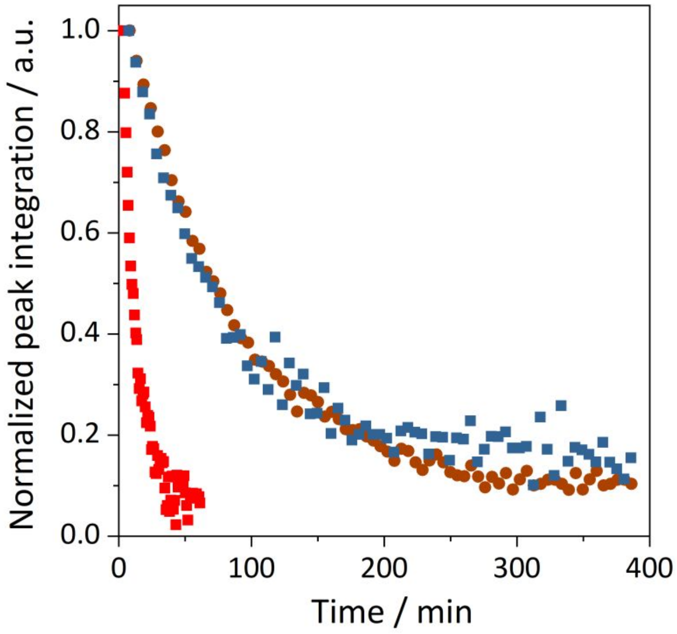

Supplementary Figure 24. Normalized aldehyde proton peak integration of *in-situ* ^{1}H-NMR measurements as a function of reaction time.

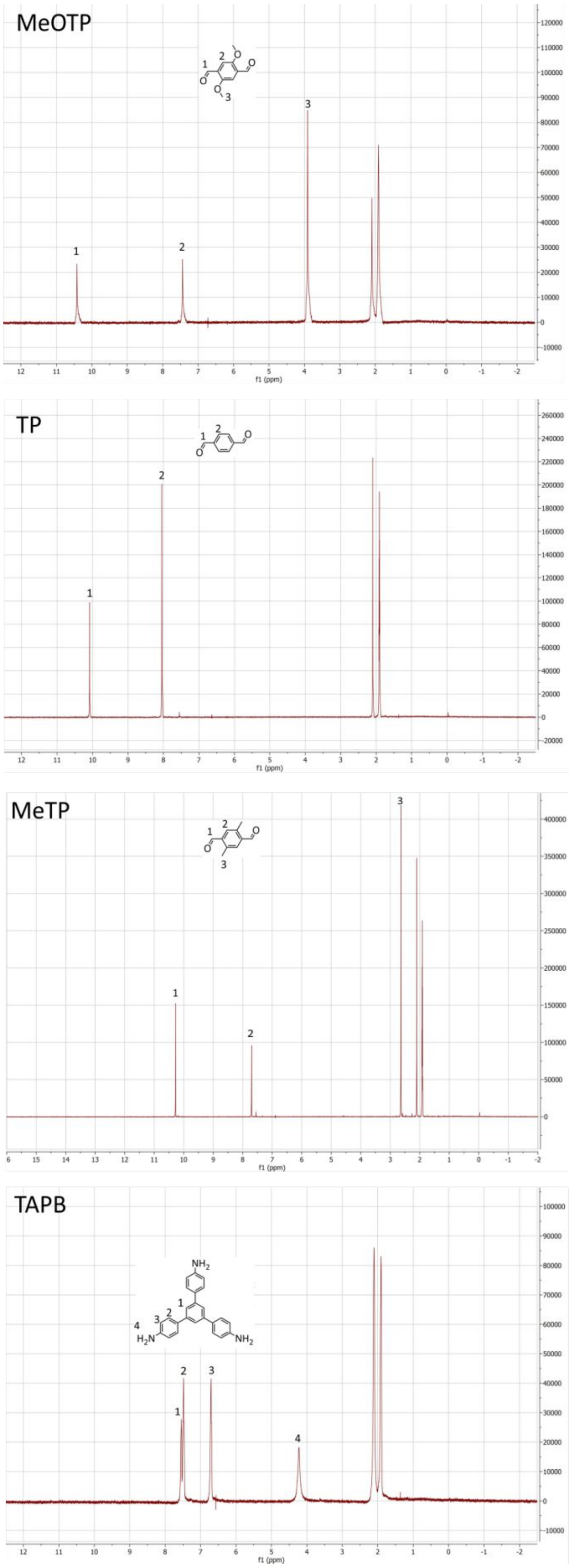

Supplementary Figure 25. ^{1}H-NMR of monomers in deuterated acetonitrile with 3.6% (v/v) of deuterated chloroform.

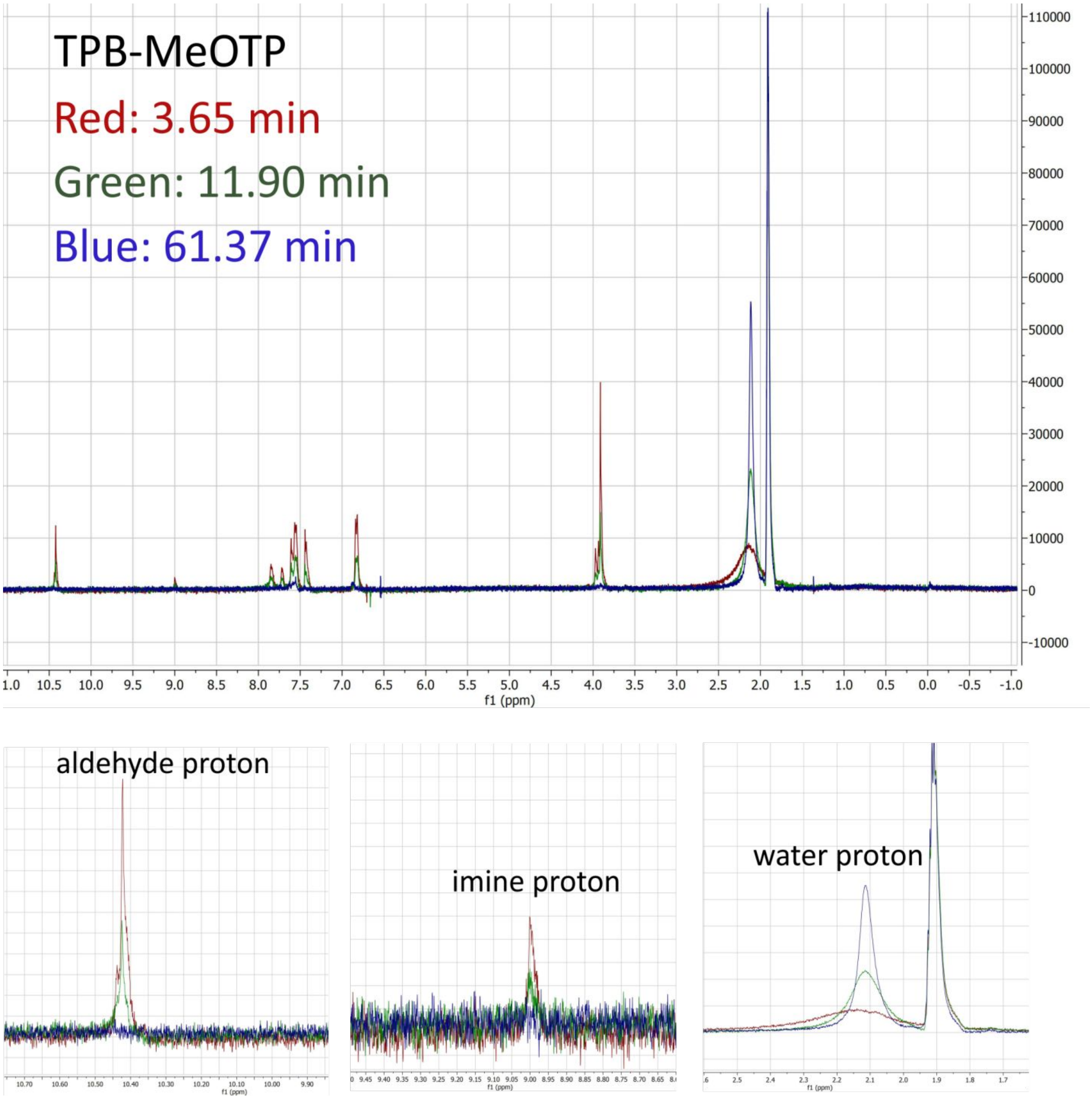

Supplementary Figure 26. Representative spectra of *in-situ* ^{1}H-NMR measurement for TPB-MeOTP colloid reaction. The regions of aldehyde proton, imine proton and water proton are shown below.

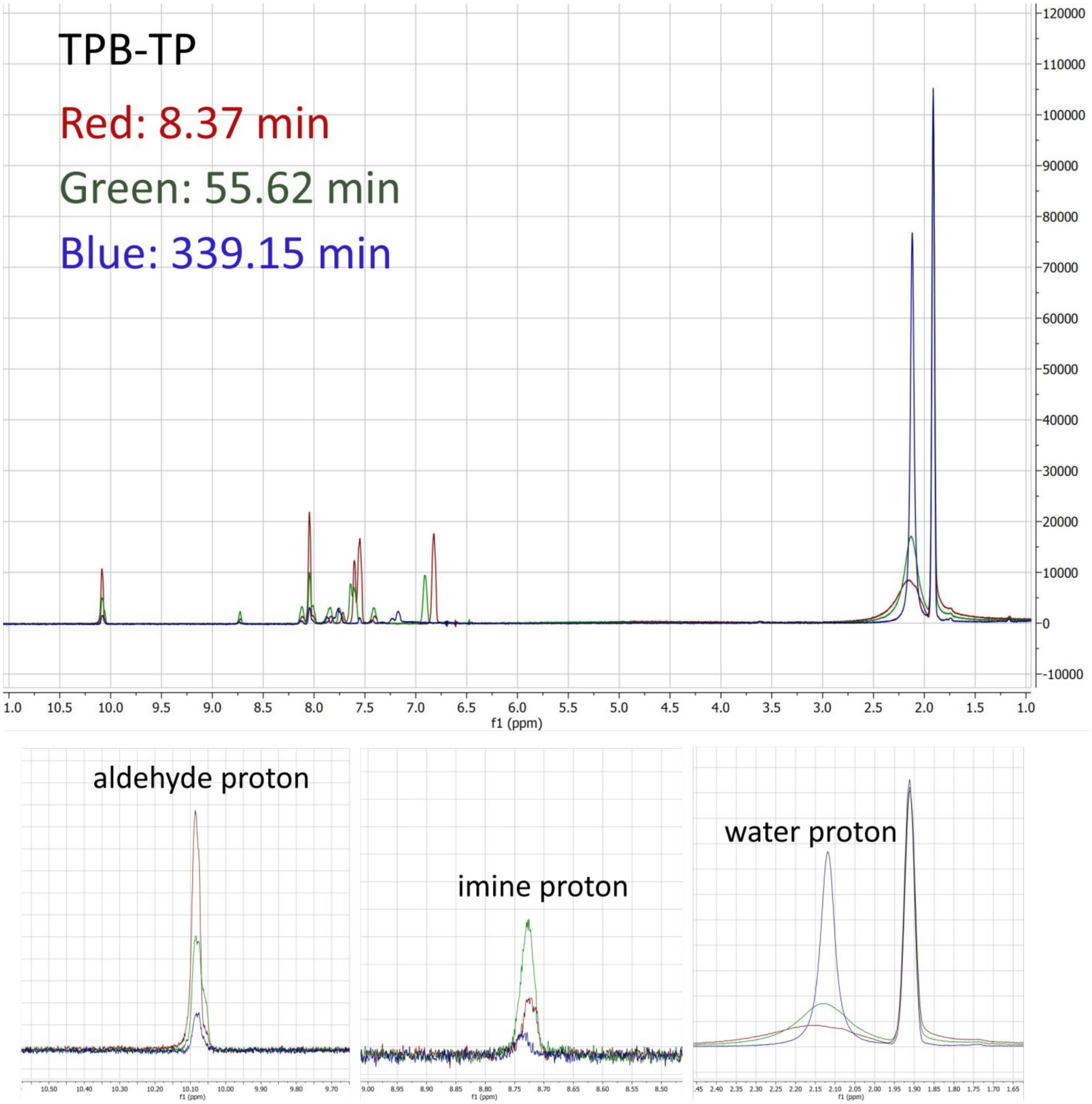

Supplementary Figure 27. Representative spectra of *in-situ* ^{1}H-NMR measurement for TPB-TP colloid reaction. The regions of aldehyde proton, imine proton and water proton are shown below.

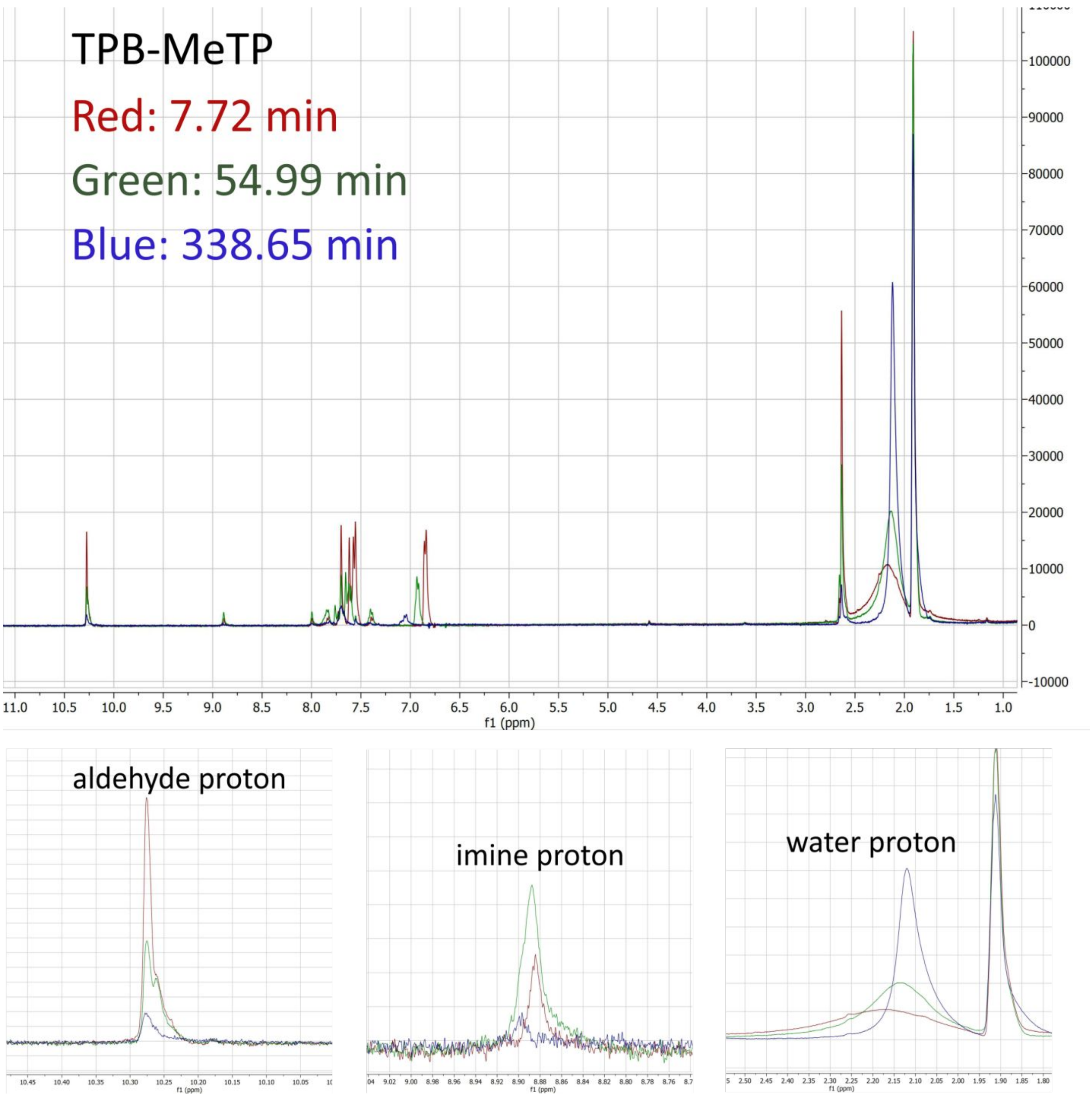

Supplementary Figure 28. Representative spectra of *in-situ* ^{1}H-NMR measurement for TPB-MeTP colloid reaction. The regions of aldehyde proton, imine proton and water proton are shown below.

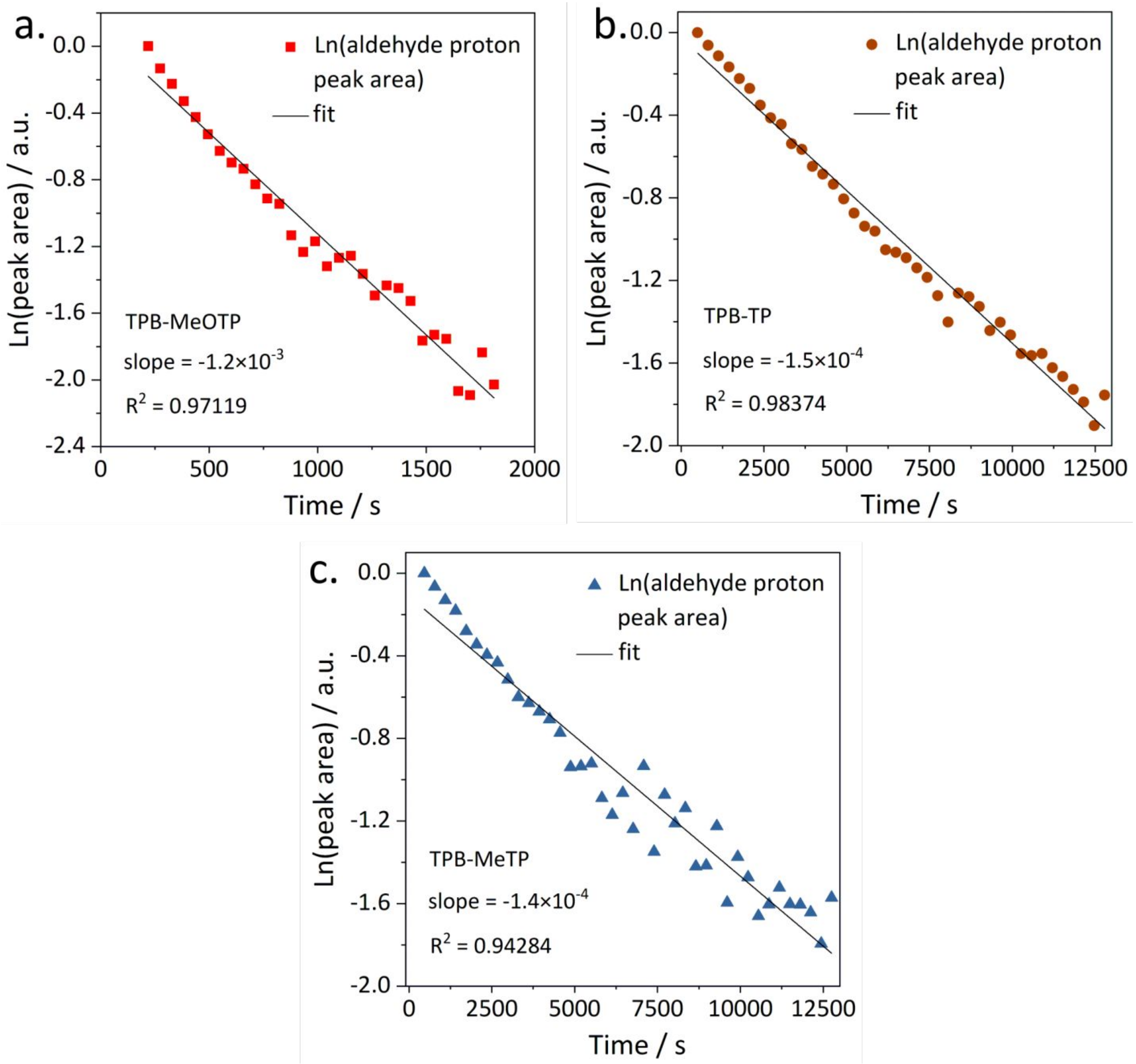

Supplementary Figure 29. The fitting of aldehyde proton integration, obtained in *in-situ* ^{1}H-NMR, by a first-order kinetic model, i.e. $I = I_0 e^{-kt}$. The natural logarithm of aldehyde proton peak area, ln(peak area), is plotted against the reaction time (t). The aldehyde consumption rate of each reaction is obtained by performing a linear fitting between ln(peak area) and reaction time (t).

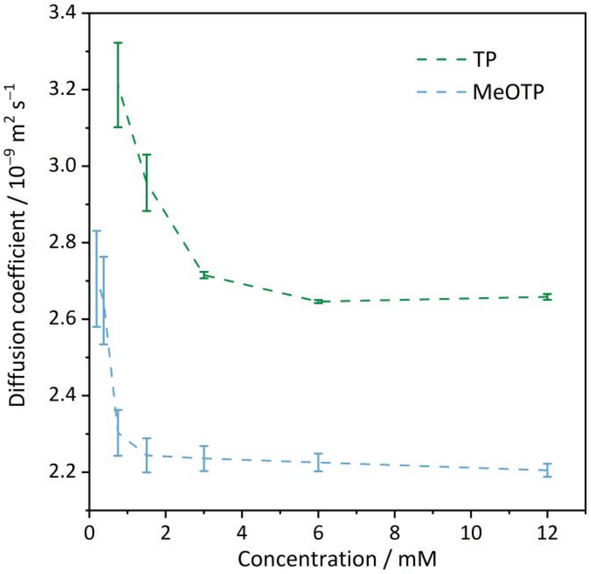

Supplementary Figure 30. Diffusion coefficients of TP and MeOTP at different concentrations in acetonitrile-d_3. The diffusion coefficient (*D*) value is correlated to the hydrodynamic volume of the diffusing species in the solution. The lower *D* in concentrated solutions is due to the self-assembly of molecules.[9] The data shows that both TP and MeOTP self-assemble at high concentration (12 mM). Nevertheless, the *D* of TP molecules starts to increase from ~6 mM, and in contrast, the *D* increase of MeOTP molecules occurs at a lower concentration (~1.5 mM). The results indicate that MeOTP has a stronger tendency to self-assemble in solution, compared to TP.

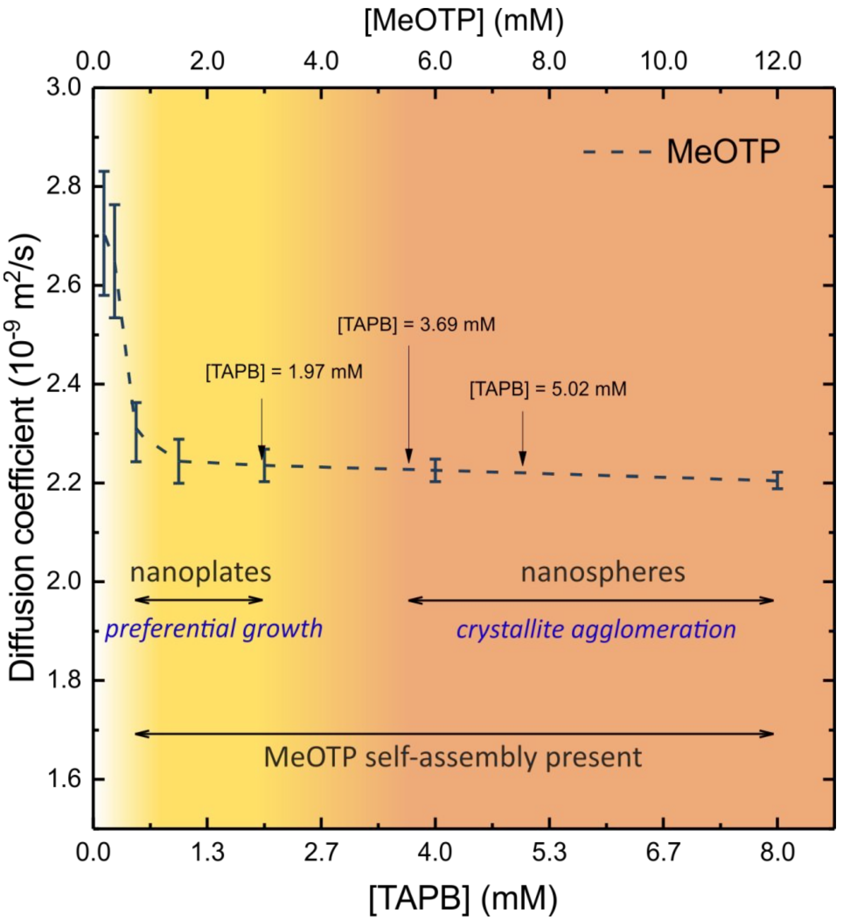

Supplementary Figure 31. Diffusion coefficients of MeOTP at different concentrations in acetonitrile-d_3. The concentration of TAPB in the corresponding reaction condition is shown as a reference. Nanoplates are achieved at low concentrations, where the self-assembly of MeOTP assists the particle growth along the interlayer interaction direction. In comparison, nanospheres are formed at high concentrations of MeOTP. This is correlated to higher concentrations, as the agglomeration of TPB-MeOTP crystallites could occur during the crystallite growth.

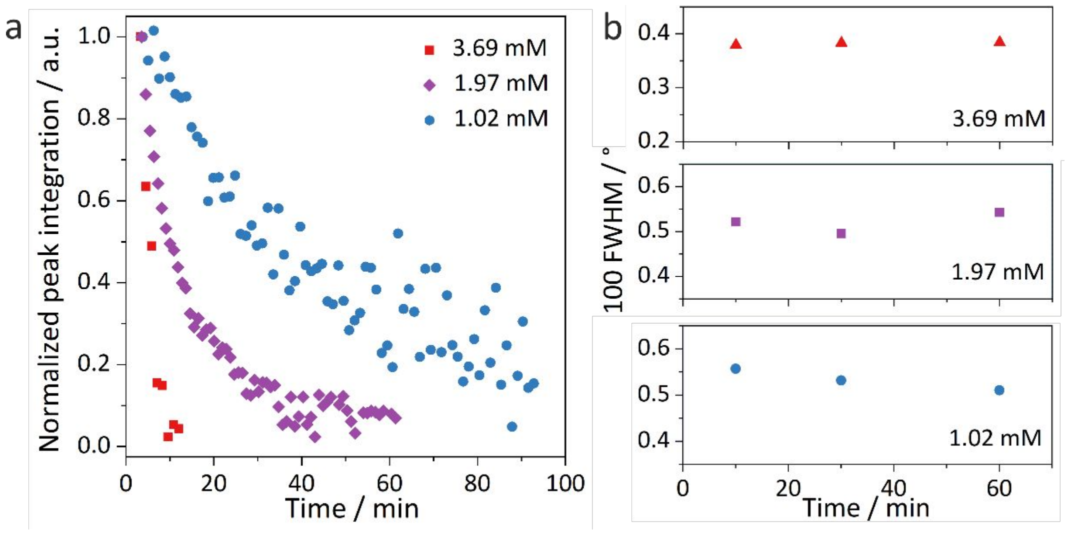

Supplementary Figure 32. a. Normalized aldehyde proton peak integration of in-situ ^{1}H-NMR measurements with [TAPB]=3.69 mM, 1.97 mM and 1.02 mM, respectively, as a function of reaction time. The ratio of amine groups and aldehyde groups is kept at 1:1. b. FWHM of 100 peak in the time dependent XRD patterns (10 min, 30 min, 60 min) with [TAPB]=3.69 mM, 1.97 mM and 1.02 mM. A higher [TAPB] results in a faster polymerization, as shown in in-situ ^{1}H-NMR measurements. The polymerization rate constant, k_{p}, was estimated by fitting the data with a first-order kinetic model ($I = I_0 e^{-kt}$), giving 6.9×10^{-3} s^{-1} for 3.69 mM, 1.2×10^{-3} s^{-1} for 1.97 mM, and 4.1×10^{-4} s^{-1} for 1.02 mM. Varying [TAPB] from 3.69 mM to 1.02 mM did not induce a visible change in crystallization rate on the time scale of tens of minutes. However, it is worth mentioning that we cannot exclude the possibility that varying [TAPB] might lead to a crystallization rate change at a shorter time scale (~tens of seconds), which is not practical to track with ex-situ techniques.

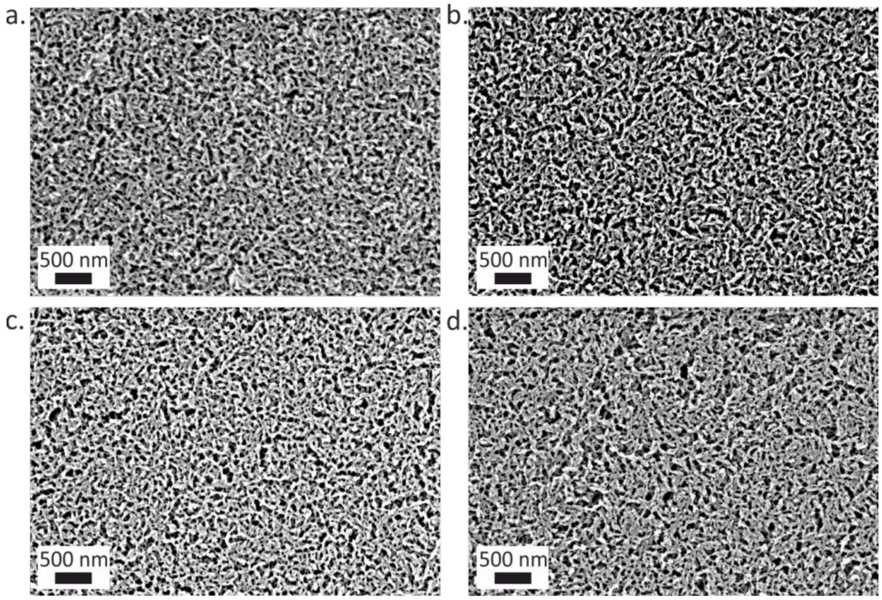

Supplementary Figure 33. Top-down SEM image of spin-coated TPB-MeOTP-NP films with 2× coating cycles (a), 4 coating cycles (b), 10 coating cycles (c) and 20 coating cycles (d). TPB-MeOTP-NP with the concentration of 4.5 mg mL^{-1} were spin-coated on FTO at 1500 rpm for 60 s. Multi coating cycles were performed with the same procedure. The resulting films were then post thermally annealed at 80 °C for 15 min in the air. The images were taken at 1.5 kV with an InLens detector. To remove the charging and achieve a high resolution, 3 nm of Ir was sputtered on the samples.

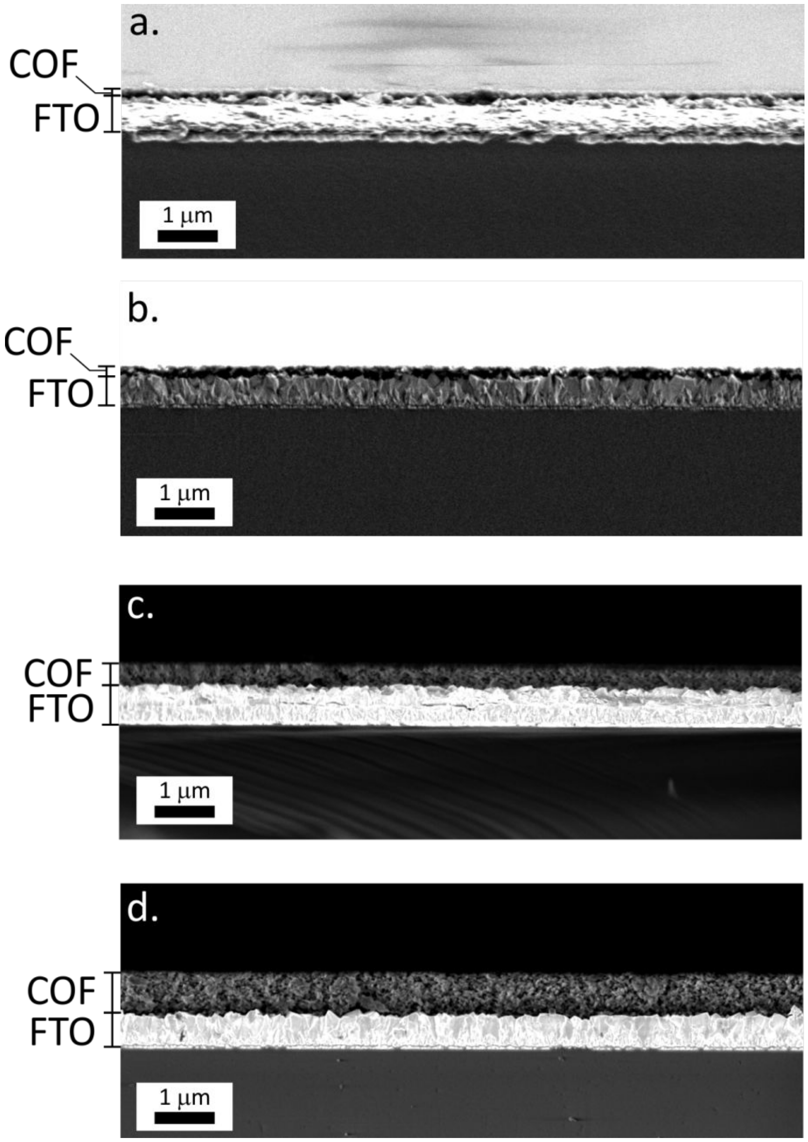

Supplementary Figure 34. Cross-sectional SEM images of FTO/TPB-MeOTP-NP films with 4× coating cycles (a), 6 coating cycles (b), 10 coating cycles (c) and 20 coating cycles (d). The TPB-MeOTP-NP film thickness is determined to be 120 nm, 160 nm, 360 nm, 660 nm, respectively. The images were taken at 1.5 kV with a SE2 detector (a, b) and an InLens detector (c, d). In these graphs, COF denotes TPB-MeOTP-NP.

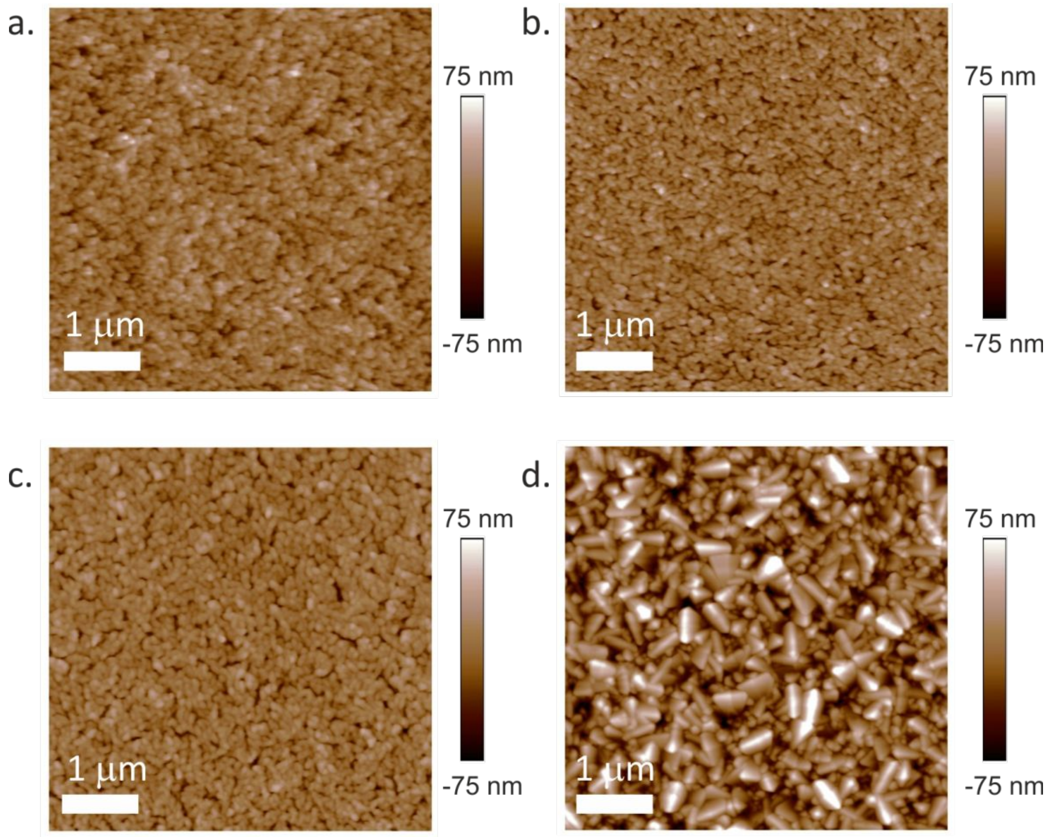

Supplementary Figure 35. AFM height images of FTO/TPB-MeOTP-NP films with 2 coating cycles (a), 4 coating cycles (b), 20 coating cycles (c) as well as a bare FTO (d). The root-mean-square (RMS) roughness is determined to be 14.6 nm (a), 14.5 nm (b), 13.1 nm (c) and 30.5 nm (d), respectively.

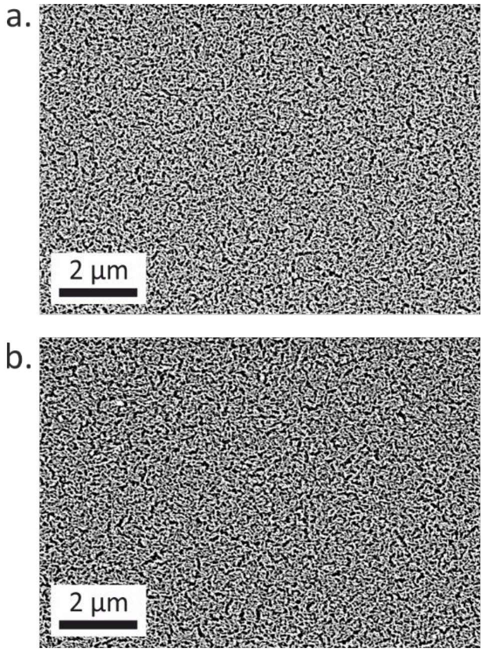

Supplementary Figure 36. Top-down SEM morphology of spin-coated TPB-MeOTP-NP on glass (a) and SiO_2/Si wafer (b). Since glass and SiO_2/Si are not conductive, 4.5 nm Ir was sputtered on the film surface to avoid the charging.

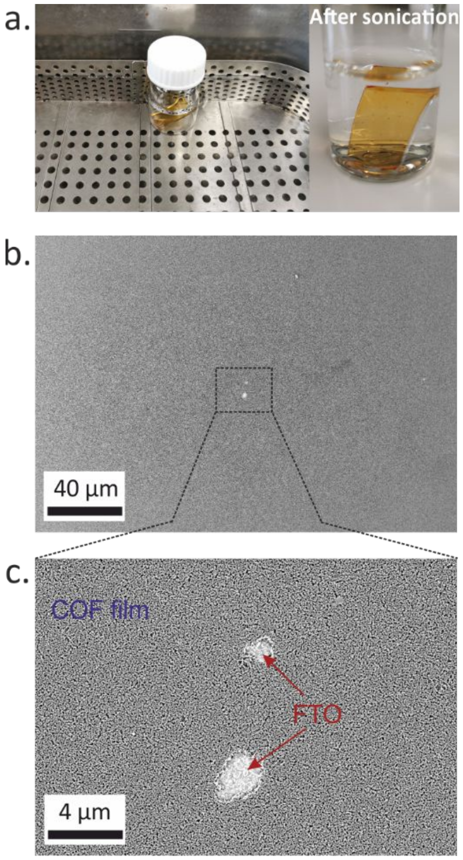

Supplementary Figure 37. Mechanical stability test of TPB-MeOTP films via sonicating the film in pH 4.2 phosphate electrolyte for 3 min, digital photographs (a) of the film during sonicating and after sonication, and SEM images (b and c) of the film after sonication.

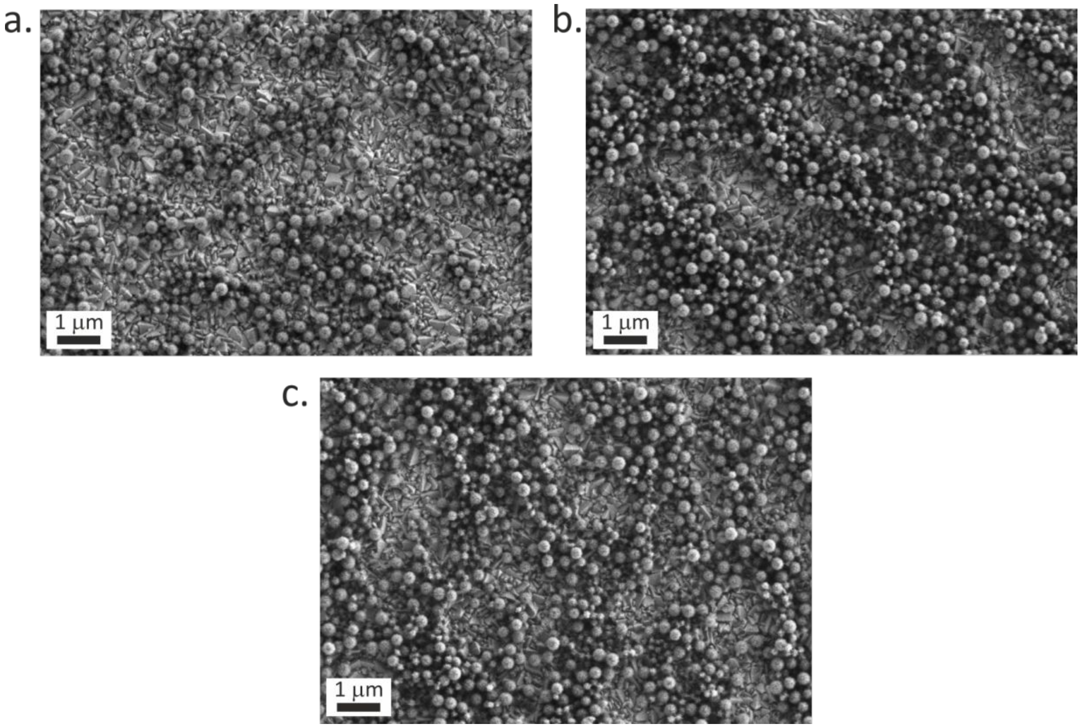

Supplementary Figure 38. Top-down SEM image of spin-coated TPB-MeOTP-NS films with 2 coating cycles (a), 4 coating cycles (b), and 6 coating cycles (c). TPB-MeOTP-NS with the concentration of 4.5 mg mL^{-1} were spin-coated on FTO at 1500 rpm for 60 s. Multi coating cycles were performed with the same procedure. The resulting films were then post thermally annealed at 80 °C for 15 min in the air. The images were taken at 1.5 kV with a SE2 detector.

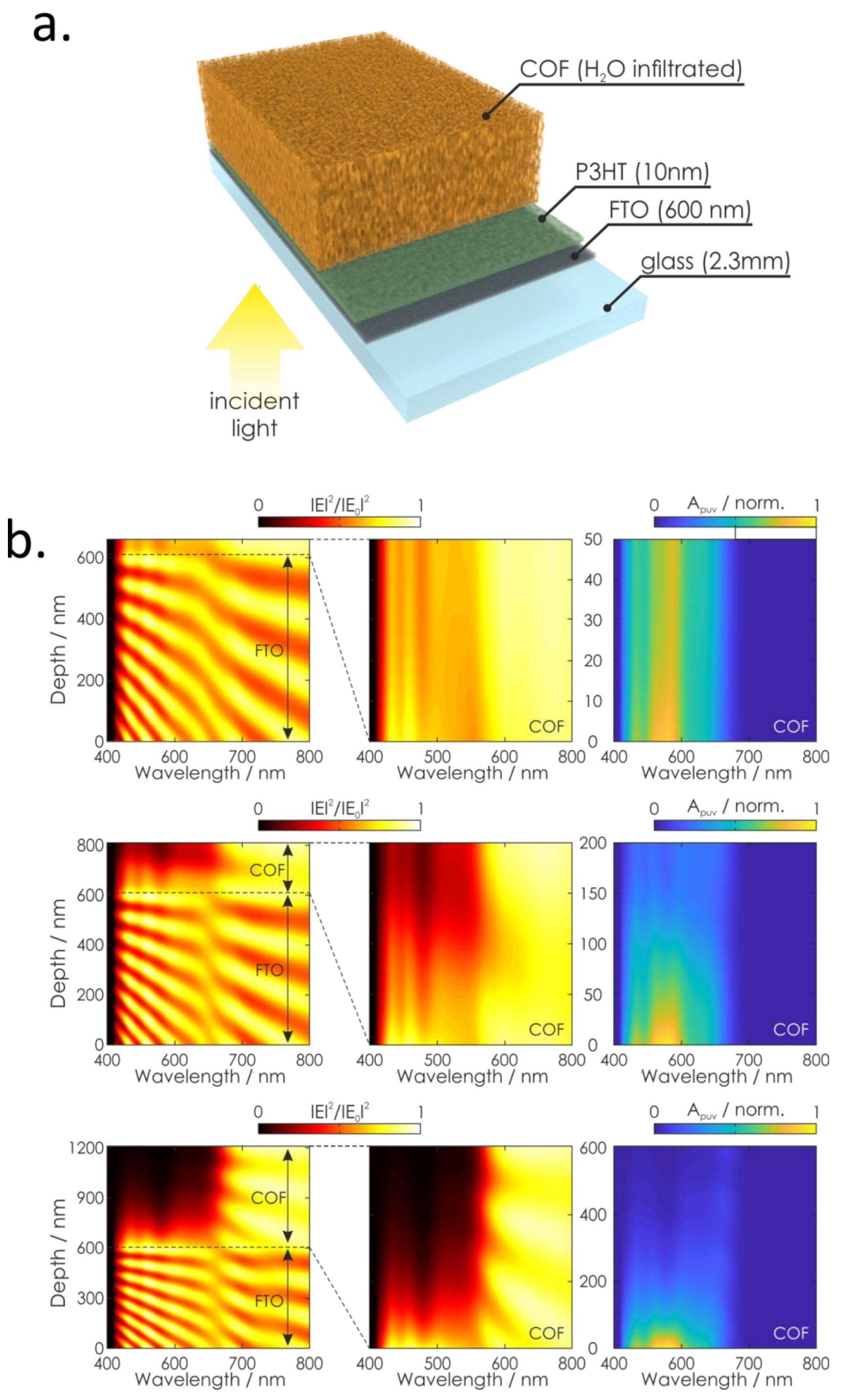

Supplementary Figure 39. Optical modeling for photoelectrochemical application. (a) Sample structure for the modeling. The system is illuminated by a plane wave injected from the glass substrate side. Calculations consider the system completely immersed in water. (b) Calculated spatial and spectral distribution of the normalized electric field intensity (left) and absorption map (right) across the section of COF layer (of 50, 200 and 600 nm) in the configuration shown in **Supplementary Fig. 39a**, assuming a plane wave propagating along the direction perpendicular to the layered surface. The modeling result indicates A_{puv} decrease rapidly with the film thickness above 150 nm, consistent with **Fig. 5g**.

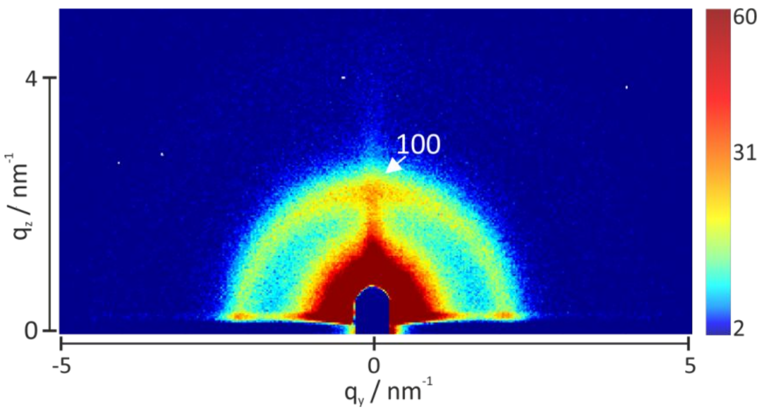

Supplementary Figure 40. GIWAXS 2D patterns of drop casted TPB-MeOTP-NP on Si wafer. A stronger reflection intensity is observed at out-of-plane direction (q_z), consistent with the spin-coating film (**Fig. 5h** in the main text).

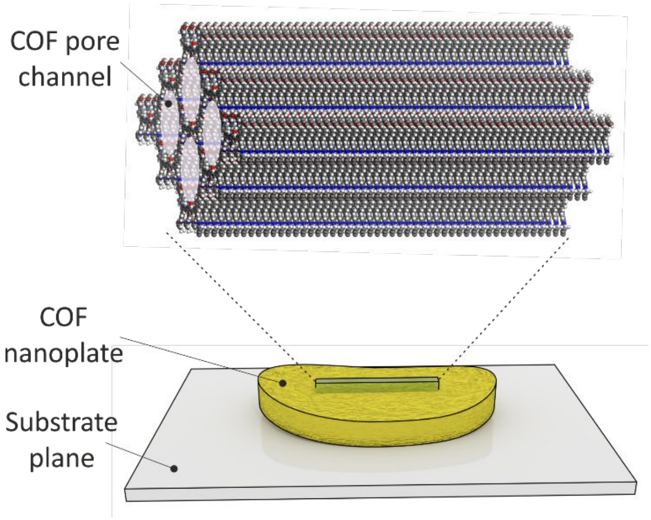

Supplementary Figure 41. Schematic illustration of the dominant crystallite orientation on the substrate. The COF pore channel of representative TPB-MeOTP-NPs is parallel to the substrate plane as suggested by GIWAXS measurements.

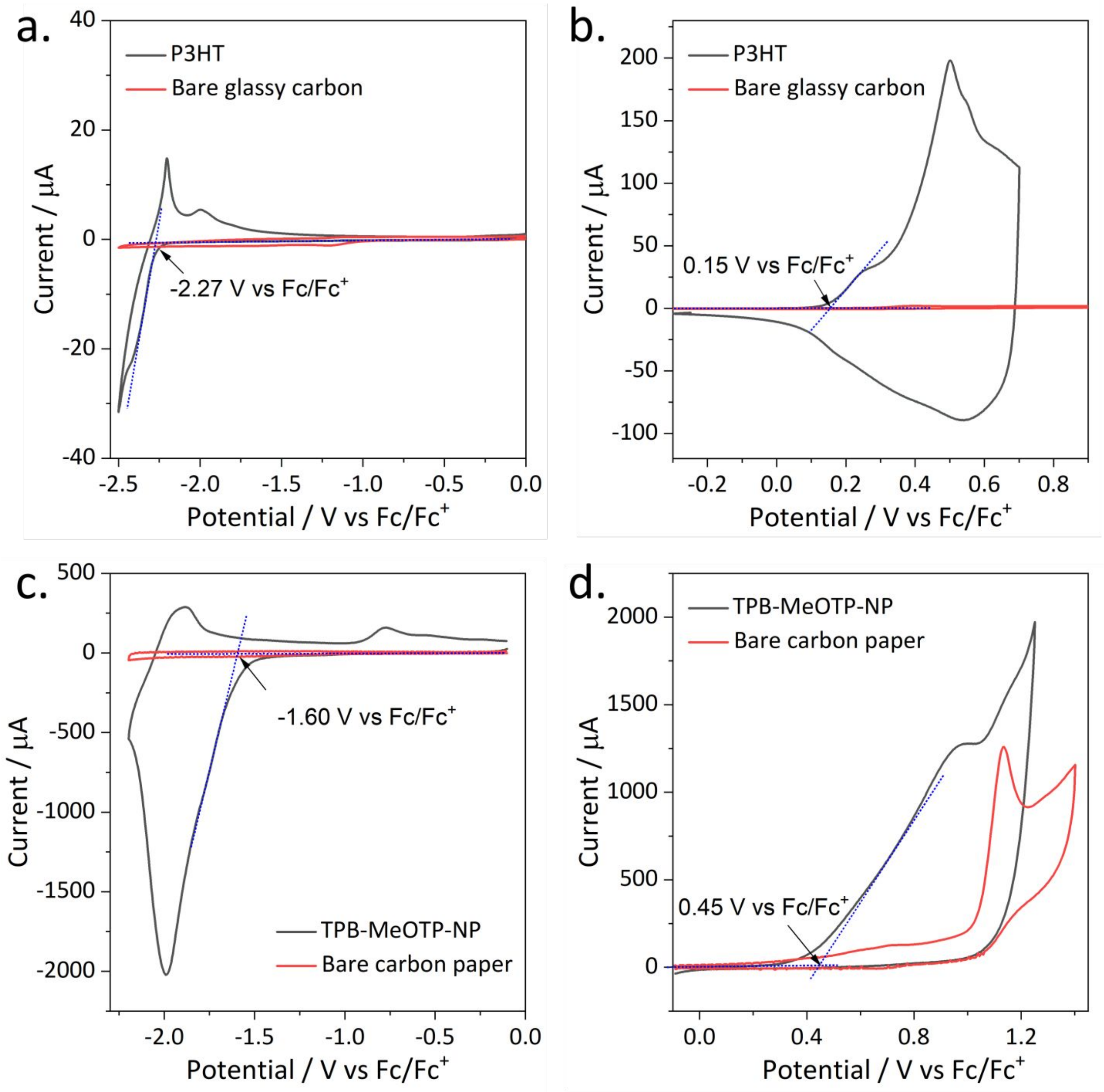

Supplementary Figure 42. Cyclic voltammograms of P3HT (a, b) and TPB-MeOTP-NP (c, d) with 0.1 M tetrabutylammonium hexafluorophosphate in anhydrous acetonitrile as supporting electrolyte. The measurements were performed in Ar atmosphere. The potential was calibrated against ferrocene/ferrocenium redox couple (−5.10 eV versus vacuum level). Glassy carbon was used for P3HT measurement, while for the TPB-MeOTP test, carbon paper (TGP-H-60, Alfa Aesar) with more electrode surface area was used as electrode. A comparative study among carbon paper, glassy carbon and FTO indicates that carbon paper results in a higher current response. Prior to the cyclic voltammetry test, the electrodes of TPB-MeOTP were washed with ethanol: water 50: 50 v/v to remove any remained solvent and $Sc(OTf)_3$, and dried under vacuum for 2 h. Cyclic voltammetry of bare glassy carbon or bare carbon paper was shown in the graphs as control test.

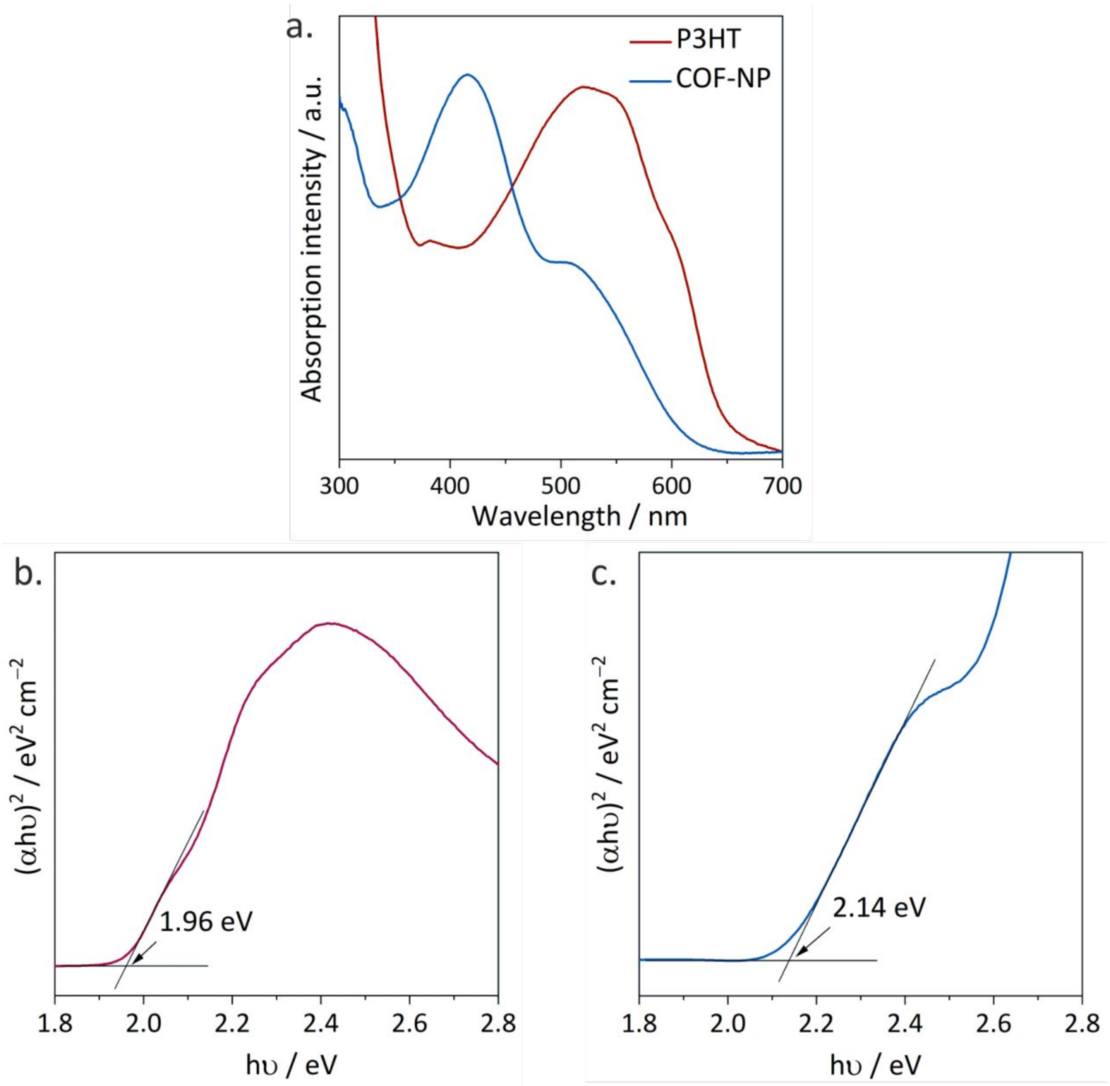

Supplementary Figure 43. a. UV-Vis absorption spectra of P3HT and TPB-MeOTP-NP films with normalized intensity. Tauc plot analysis of P3HT (b) and TPB-MeOTP-NP film (c), suggesting an indirect band gap of 1.96 eV and 2.14 eV, respectively.

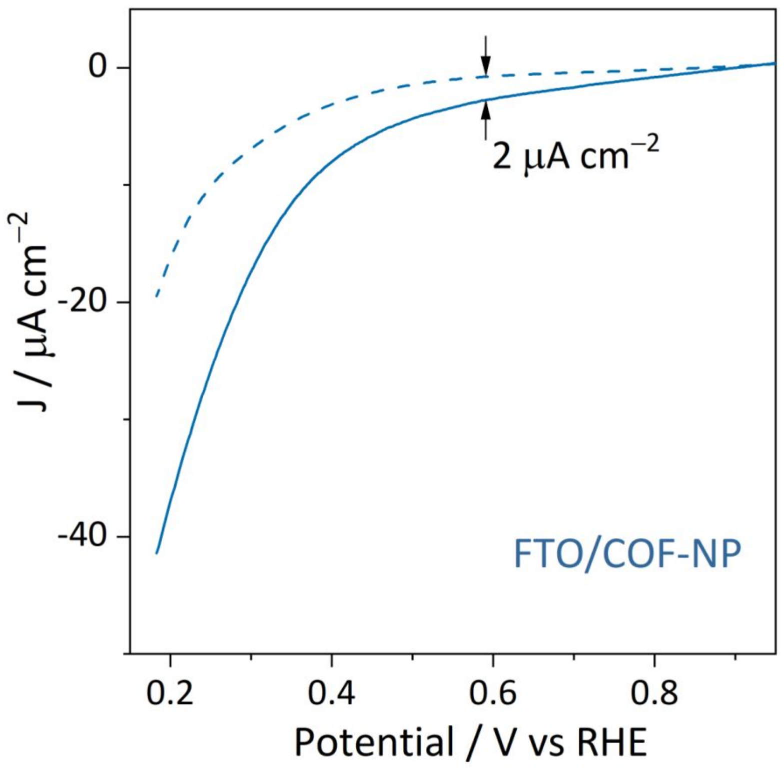

Supplementary Figure 44. LSV of FTO/TPB-MeOTP-NP (4× coatings) performed in 0.5 M Eu^{3+} aqueous electrolyte under illumination (solid line) and in the dark (dash line). The scan rate is 10 mV s^{-1}. COF-NP in the graph denotes TPB-MeOTP-NP.

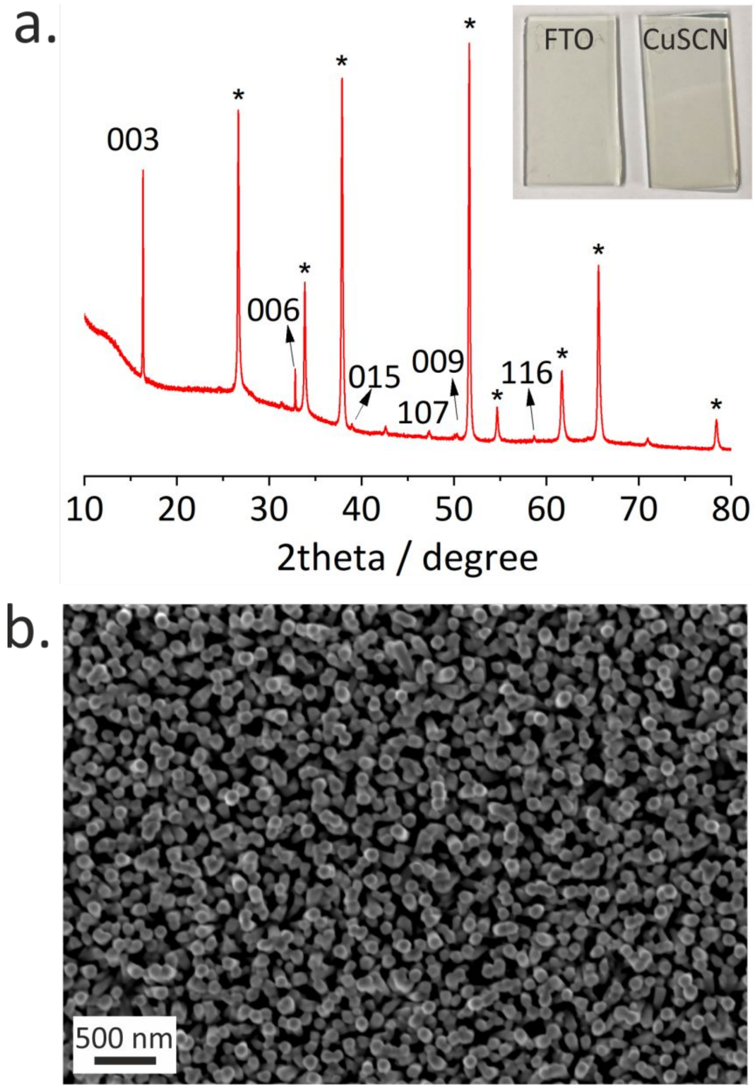

Supplementary Figure 45. XRD patterns (a) and top-down SEM image (b) of electrodeposited CuSCN on FTO substrate with 5 min electrodeposition duration at -0.3 V vs Ag/AgCl. Digital images of bare FTO and FTO/CuSCN are displayed in (a). The images were taken at 1.5 kV with an InLens detector. Asterisks indicate the peaks originated from the FTO substrate.

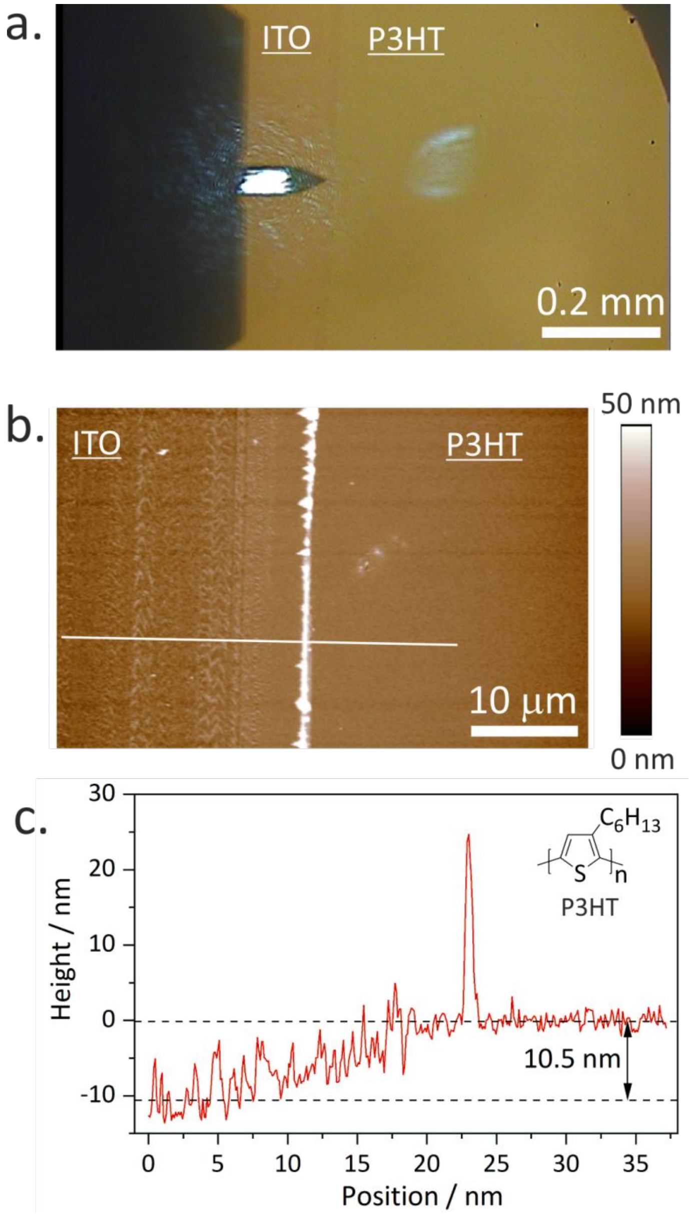

Supplementary Figure 46. AFM measurement for determining P3HT thickness. P3HT layer was prepared by spin coating P3HT solution in chloroform (1 mg mL^{-1}) on indium-doped tin oxide (ITO) substrate at 1500 rpm for 60 s, followed by a thermal annealing at 80 °C for 10 min in the air. To measure the film thickness, part of P3HT film was removed, as shown in the optical microscope image (a). (b) and (c) show the AFM height image and the height plot along the line in the height image, respectively. The molecular structure of P3HT is also shown in the inset of (c).

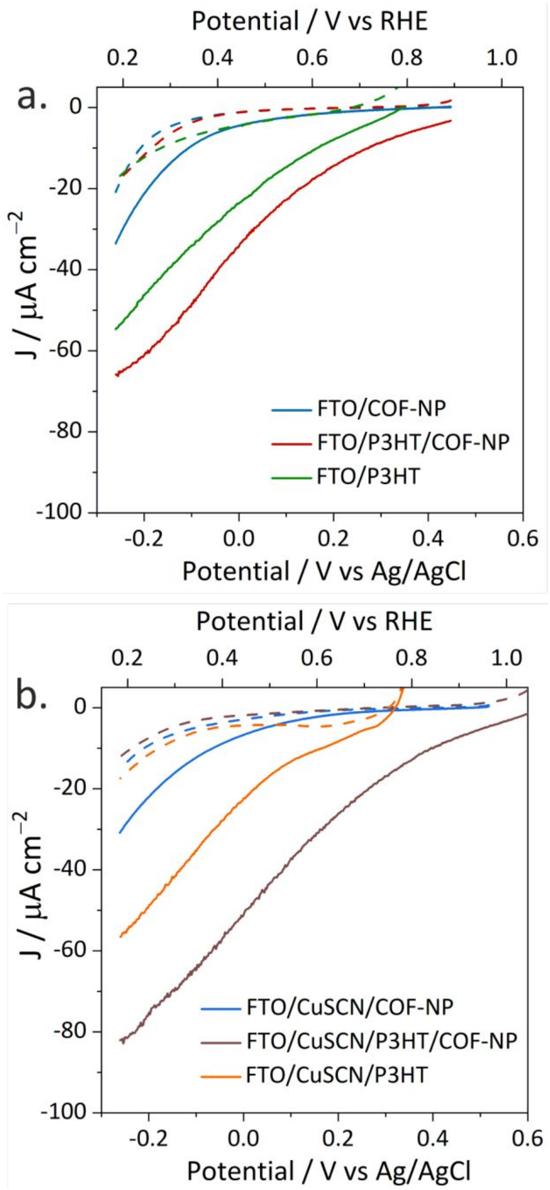

Supplementary Figure 47. PEC characterization of TPB-MeOTP-NP (4× coatings) photocathodes in 0.5 M Eu^{3+} aqueous electrolyte. The scan rate of the LSV curves is 10 mV s^{-1}. Various photocathode structures are compared to gain insights about the function of CuSCN nanowires and P3HT layer. 10 nm P3HT layer generates the J_{ph} of 53.0 µA cm^{-2} for FTO/P3HT and 54.8 µA cm^{-2} for FTO/CuSCN/P3HT under illumination at +0.20 V_{RHE}, as P3HT is a common photoactive donor polymer in organic solar cells. Nevertheless, P3HT layer exhibits anodic current at +0.79 V_{RHE} for FTO/ P3HT and +0.76 V_{RHE} for FTO/CuSCN/P3HT even under dark condition, very likely due to the oxidation of P3HT as the HOMO level locates at about +0.81 V_{RHE}. The P3HT/COF-NP heterojunction positively shifted the V_{on} in both cases with and without CuSCN nanowires. All the CA tests were collected at +0.7 V_{RHE} in this work, since the photocurrent contribution from P3HT is negligible at this potential, as indicated in Fig. 6d in the main text. Introducing CuSCN nanowires reduces the dark current density of FTO/P3HT/COF-NP (16.9 µA cm^{-2}) at +0.20 V_{RHE} to 10.9 µA cm^{-2}, and increases the J_{ph} from 65.2 µA cm^{-2} to 81.6 µA cm^{-2}, suggesting a reduced recombination for hole extraction.

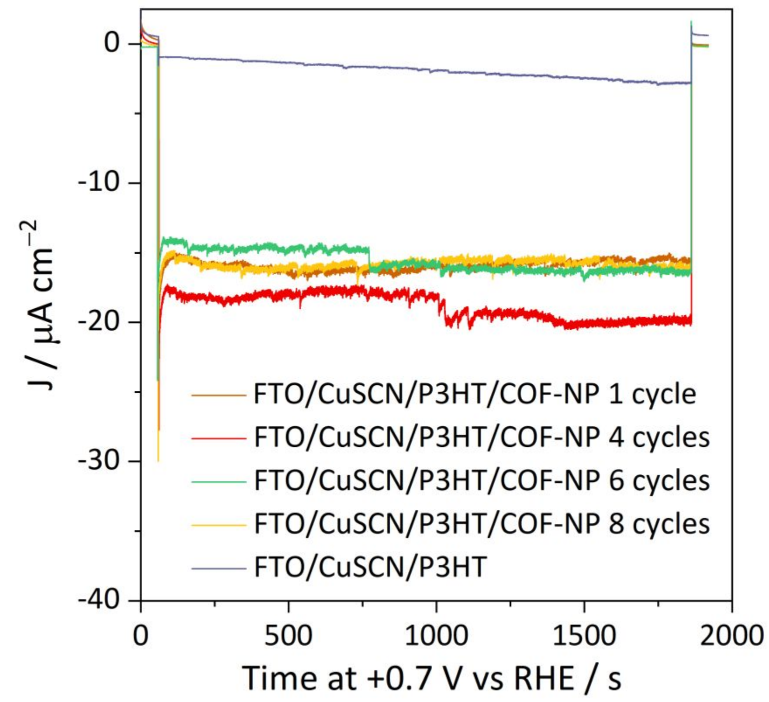

Supplementary Figure 48. PEC characterization of TPB-MeOTP-NP photocathodes with different TPB-MeOTP-NP coating cycles in 0.5 M Eu^{3+} aqueous electrolyte. COF-NP in the graph denotes TPB-MeOTP-NP.

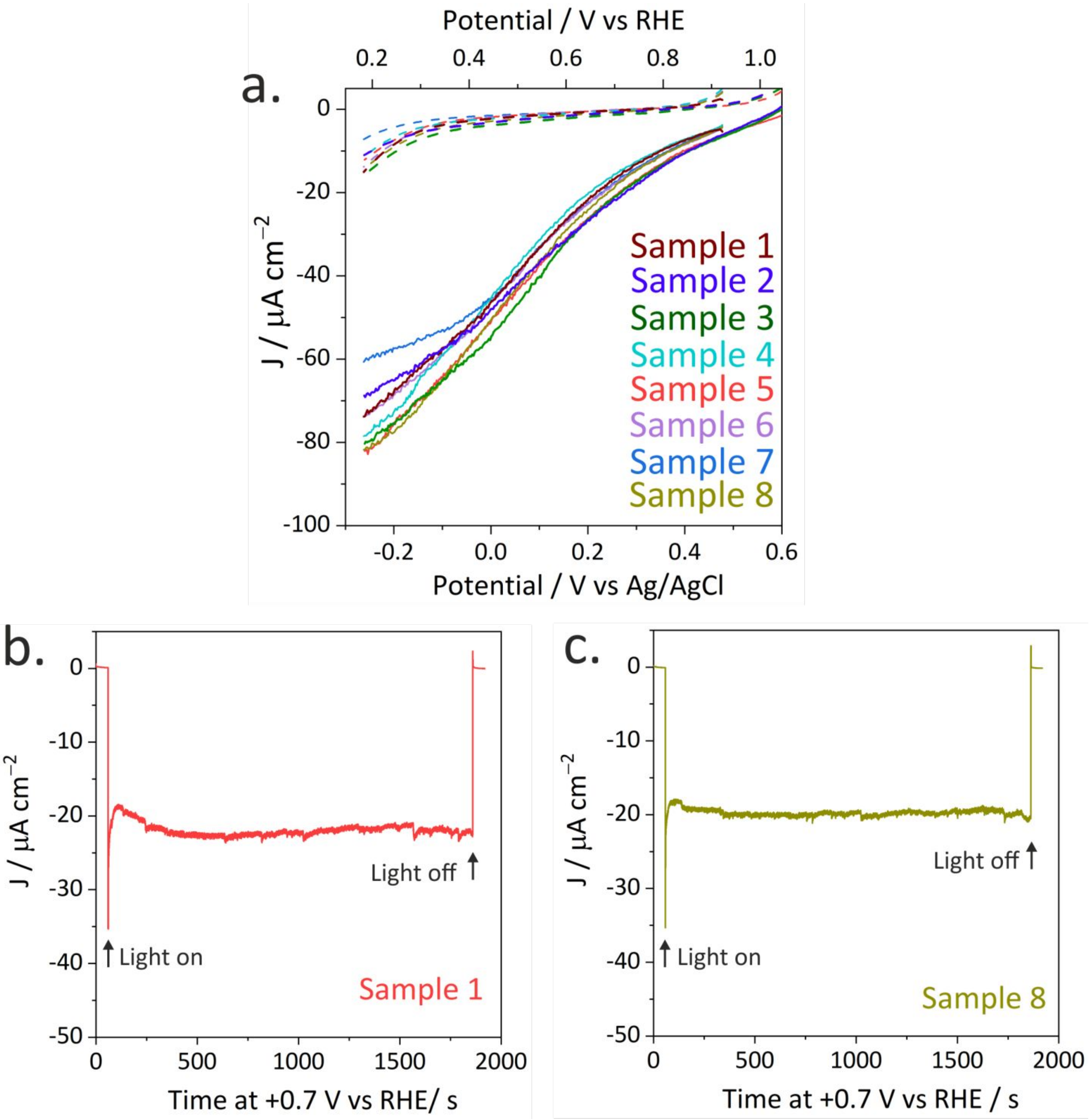

Supplementary Figure 49. PEC characterization of CuSCN/P3HT/TPB-MeOTP-NP (4× coatings) photocathodes in 0.5 M Eu^{3+} aqueous electrolyte. LSV curves (a) of eight different samples performed under illumination (solid lines) and in the dark (dashed lines), and CA curves (b) at +0.7 V_{RHE} of two additional samples besides the one displayed in Fig. 6d. The scan rate of LSV scans is 10 mV s^{-1}.

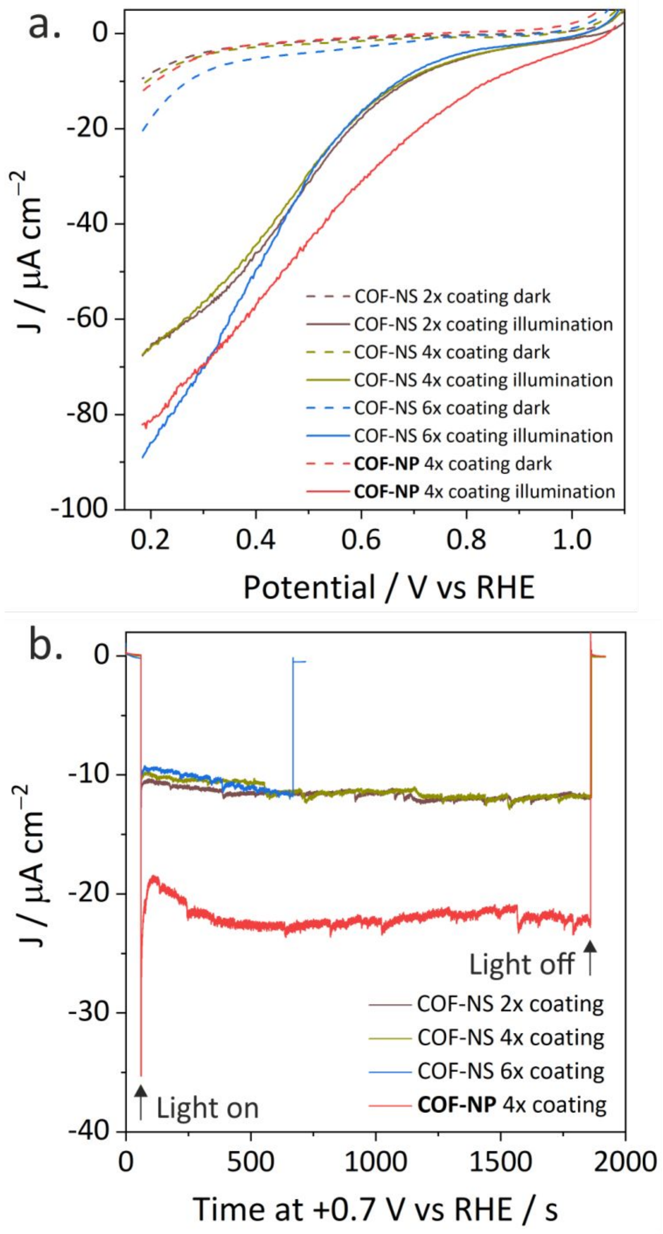

Supplementary Figure 50. PEC characterization of CuSCN/P3HT/TPB-MeOTP-NS (2×, 4× and 6× coatings) photocathodes in 0.5 M Eu^{3+} aqueous electrolyte. LSV curves (a) performed under illumination (solid line) and in the dark (dash line), and CA curves (b) at +0.7 V_{RHE}. Representative LSV and CA curves of CuSCN/P3HT/TPB-MeOTP-NS (4× coatings) photocathodes are shown for comparison. The scan rate of LSV scans is 10 mV s^{-1}. COF-NP and COF-NS in the graph denote TPB-MeOTP-NP and TPB-MeOTP-NS, respectively.

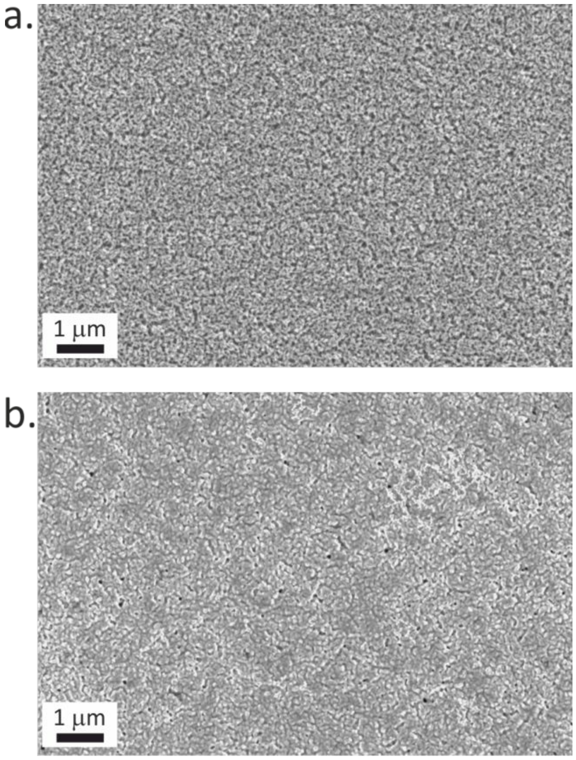

Supplementary Figure 51. Top-down SEM image of CuSCN/P3HT/TPB-MeOTP-NP (a) and CuSCN/P3HT/TPB-MeOTP-NP/Pt catalyst overlayer (b). The images were taken at 1.5 kV with an InLens detector.

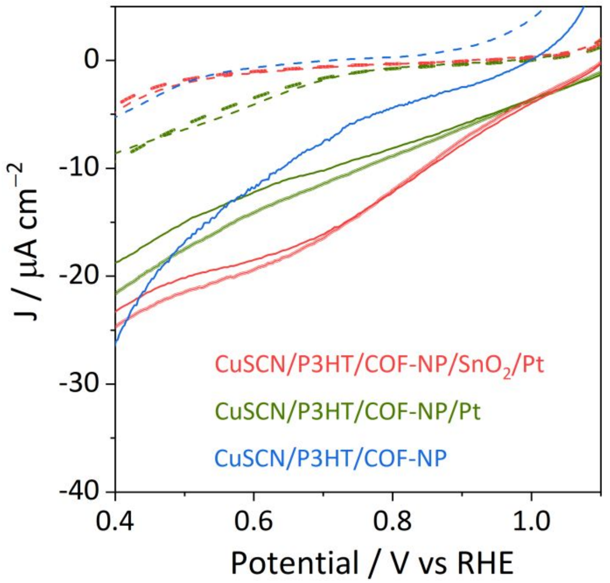

Supplementary Figure 52. LSV performed in 0.5 M NaH_2PO_4 aqueous electrolyte under illumination (solid line) and in the dark (dash line). Two different samples of CuSCN/P3HT/TPB-MeOTP-NP/SnO_2/Pt and CuSCN/P3HT/TPB-MeOTP-NP/Pt are shown. The scan rate is 10 mV s^{-1}. COF-NP in the graph denotes TPB-MeOTP-NP.

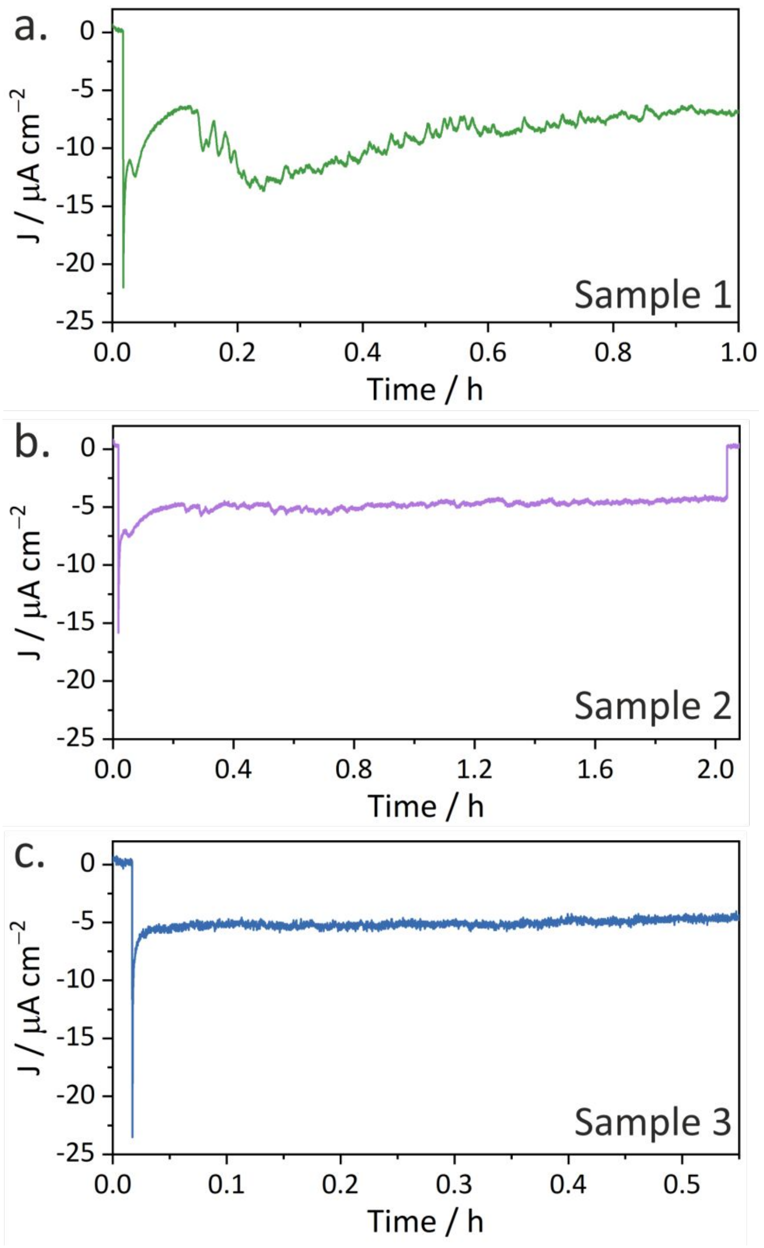

Supplementary Figure 53. CA curves at +0.7 V_{RHE} in 0.5 M NaH_2PO_4 aqueous electrolyte of three additional samples besides the one shown in Fig 6f.

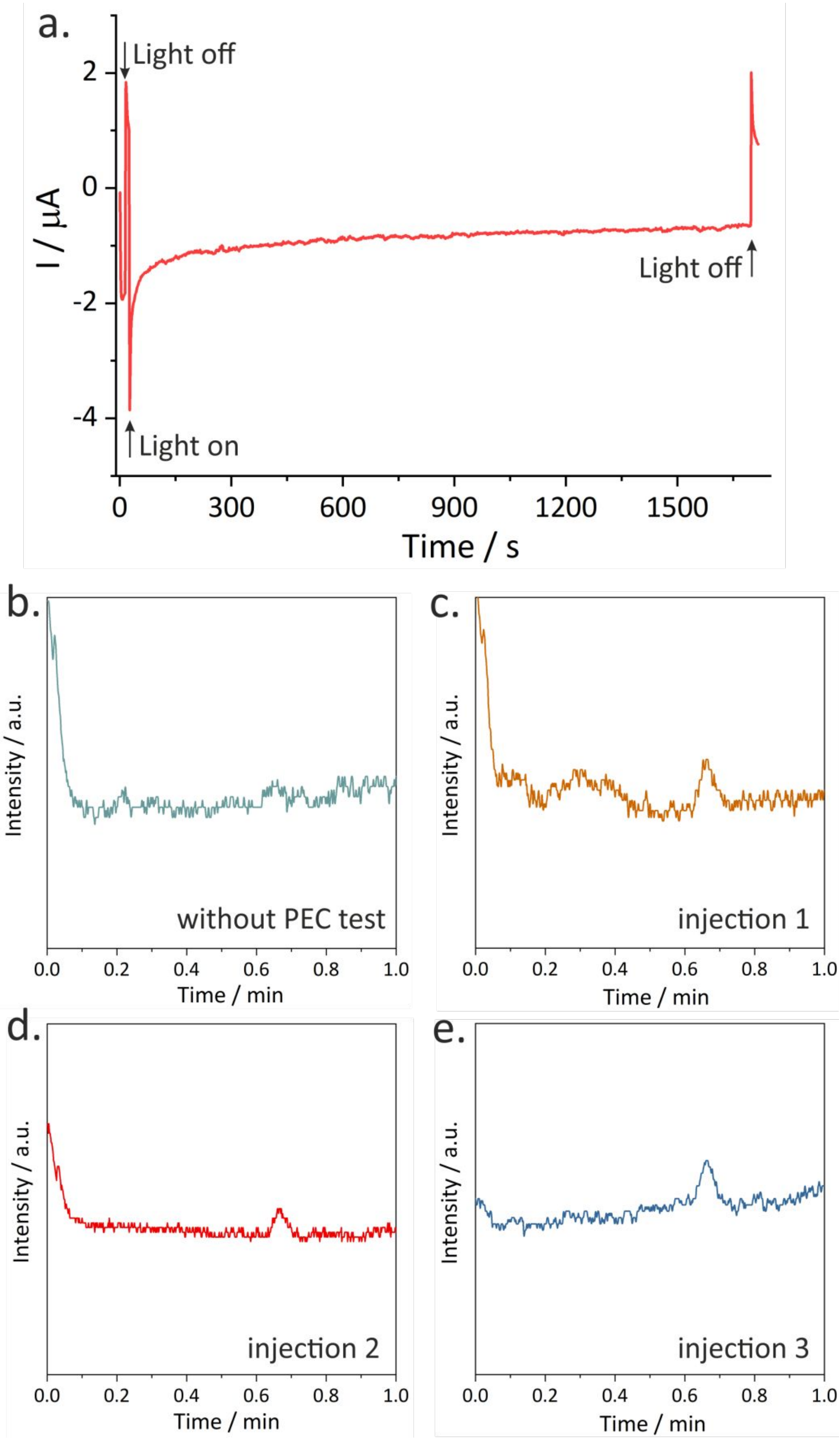

Supplementary Figure 54. H_2 detection of CuSCN/P3HT/TPB-MeOTP-NP/SnO_2/Pt photocathode with the working area of 0.6 cm^2. CA curve (a) at +0.7 V_{RHE}, and gas chromatograms acquired without PEC test (b), at 395 s (c), at 1045 s (d), and at 1695 s (e).

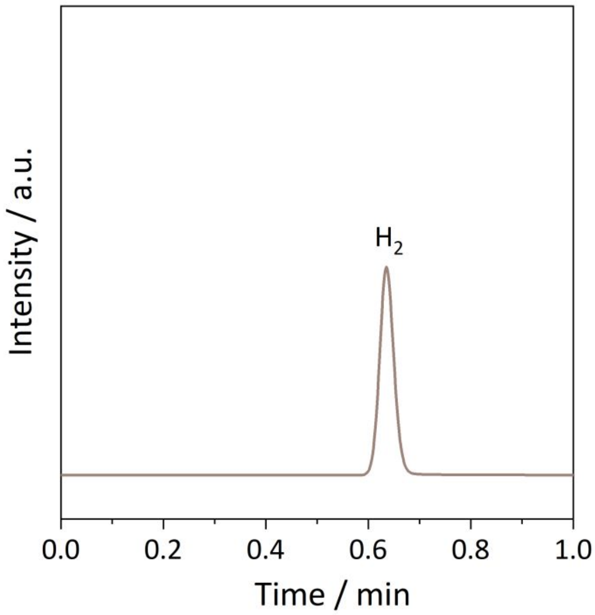

Supplementary Figure 55. Retention time of standard hydrogen calibration gas.

Supplementary Table 5. Comparison of reported COF photocathodes.

Publication	Photocathode structure	V_{on}[1]	J_{ph} at +0.7 V_{RHE}
**This work**	FTO/CuSCN/P3HT/TPB-MeOTP-NP/SnO_2/Pt	**+1.06 V_{RHE}**	**17 μA cm^{-2}**
Adv. Mater. Interfaces, 2021, 8, 2002191	FTO/TTA-TTB<br>FTO/TAPB-TTB	Below +0.6 V_{RHE}	-----
Adv. Mater. 2021, 33, 2006274	FTO/2D CCP-Th<br>FTO/2D CCP-BD<br>FTO/ 2D C=N COF-B	Below +0.6 V_{RHE}	-----
Chem. Mater. 2019, 31, 10008	FTO/ BDT-ETTA oriented<br>FTO/ BDT-ETTA EPD<br>FTO/ BDT-ETTA EPD/Pt	~+0.5 V_{RHE}	-----
Adv. Energy Mater. 2018, 8, 1703278	FTO/A-TENPY<br>FTO/A-TEBPY	~+0.35 V_{RHE}	-----
J. Am. Chem. Soc. 2018, 140, 2085	ITO/ BDT-ETTA/Pt	~+0.96 V_{RHE}	3~4 μA cm^{-2}

[1]V_{on} is defined as the potential to obtain a J_{ph} of 2 μA cm^{-2}.

5. Supplementary References

(1) Pan, L.; Liu, Y.; Yao, L.; Dan, R.; Sivula, K.; Grätzel, M.; Hagfeldt, A., Cu2O photocathodes with band-tail states assisted hole transport for standalone solar water splitting. *Nat. Commun.* **2020,** *11* (1), 318.
(2) Yang, D.; Yang, R.; Wang, K.; Wu, C.; Zhu, X.; Feng, J.; Ren, X.; Fang, G.; Priya, S.; Liu, S., High efficiency planar-type perovskite solar cells with negligible hysteresis using EDTA-complexed SnO2. *Nat. Commun.* **2018,** *9* (1), 3239.
(3) Li, Rebecca L.; Flanders, N. C.; Evans, A. M.; Ji, W.; Castano, I.; Chen, L. X.; Gianneschi, N. C.; Dichtel, W. R., Controlled growth of imine-linked two-dimensional covalent organic framework nanoparticles. *Chem. Sci.* **2019,** *10* (13), 3796-3801.
(4) Xu, H.; Gao, J.; Jiang, D., Stable, crystalline, porous, covalent organic frameworks as a platform for chiral organocatalysts. *Nat. Chem.* **2015,** *7* (11), 905-912.
(5) Forouhi, A. R.; Bloomer, I., Optical dispersion relations for amorphous semiconductors and amorphous dielectrics. *Phys. Rev. B* **1986,** *34* (10), 7018-7026.
(6) Forouhi, A. R.; Bloomer, I., Optical properties of crystalline semiconductors and dielectrics. *Phys. Rev. B* **1988,** *38* (3), 1865-1874.
(7) Anaya, M.; Lozano, G.; Calvo, M. E.; Zhang, W.; Johnston, M. B.; Snaith, H. J.; Míguez, H., Optical Description of Mesostructured Organic–Inorganic Halide Perovskite Solar Cells. *J. Phys. Chem. Lett.* **2014,** *6* (1), 48-53.
(8) Macchioni, A.; Ciancaleoni, G.; Zuccaccia, C.; Zuccaccia, D., Determining accurate molecular sizes in solution through NMR diffusion spectroscopy. *Chem. Soc. Rev.* **2008,** *37* (3), 479-489.
(9) Majer, G.; Zick, K., Accurate and absolute diffusion measurements of Rhodamine 6G in low-concentration aqueous solutions by the PGSE-WATERGATE sequence. *J. Chem. Phys.* **2015,** *142* (16).
(10) Majer, G.; Melchior, J. P., Characterization of the fluorescence correlation spectroscopy (FCS) standard Rhodamine 6G and calibration of its diffusion coefficient in aqueous solutions. *J. Chem. Phys.* **2014,** *140* (9).
(11) Tanner, J. E., Use of the Stimulated Echo in NMR Diffusion Studies. *J. Chem. Phys.* **1970,** *52* (5), 2523-2526.
